# Synthesis, Antibacterial and Antitubercular Activities of Some 5*H*-Thiazolo[3,2-*a*]pyrimidin-5-ones and Sulfonic Acid Derivatives

**DOI:** 10.3390/molecules200916419

**Published:** 2015-09-10

**Authors:** Dong Cai, Zhi-Hua Zhang, Yu Chen, Xin-Jia Yan, Liang-Jing Zou, Ya-Xin Wang, Xue-Qi Liu

**Affiliations:** 1 College of Basic Science, Liaoning Medical University, Jinzhou 121001, China; E-Mails: zouliangjing@yeah.net (L.-J.Z.); 18341653394@163.com (Y.-X.W.); araqi1128@163.com (X.-Q.L.); 2 School of Chemical and Environmental Engineering, Liaoning University of Technology, Jinzhou 121001, China; E-Mail: Bridge1026@163.com; 3 School of Life Science and Biopharmaceutics, Shenyang Pharmaceutical University, Shenyang 110016, China; E-Mail: gzweishengwu@126.com; 4 College of Pharmacy, Harbin University of Commerce Harbin, Harbin 150076, China; E-Mail: yanxinjia@yeah.net

**Keywords:** thiazolo[3,2-*a*]pyrimidine, sulfonation, antibacterial, antitubercular

## Abstract

A series of 5*H*-thiazolo[3,2-*a*]pyrimidin-5-ones were synthesized by the cyclization reactions of *S*-alkylated derivatives in concentrated H_2_SO_4_. Upon treatment of *S*-alkylated derivatives at different temperatures, intramolecular cyclization to 7-(substituted phenylamino)-5*H*-thiazolo[3,2-*a*]pyrimidin-5-ones or sulfonation of cyclized products to sulfonic acid derivatives occurred. The structures of the target compounds were confirmed by IR, ^1^H-NMR, ^13^C-NMR and HRMS studies. The compounds were evaluated for their preliminary *in vitro* antibacterial activity against some Gram-positive and Gram-negative bacteria and screened for antitubercular activity against *Mycobacterium tuberculosis* by the broth dilution assay method. Some compounds showed good antibacterial and antitubercular activities.

## 1. Introduction

The rapid development of bacterial drug resistance is growing into a global problem. Consequently, there is a pressing need to develop new antimicrobial drugs with potent activity in order to overcome the bacterial drug resistance. Electron-rich nitrogen heterocycles and sulfur compounds play an important role in diverse biological activities. Thiazolo[3,2-*a*]pyrimidine nucleus have been consistently regarded as structural analogs of biogenic purine bases and can be considered as potential purine antagonists [1,2]. These heterocyclic systems are the key chemical building blocks for numerous compounds that play important roles in the functioning of biologically active molecules. As one type of those heterocyclic rings, 5*H*-thiazolo[3,2-*a*]pyrimidin-5-ones are considered a promising class of bioactive heterocyclic compounds encompassing a diverse range of biological activities such as anti-inflammatory [3,4], antihypertensive [5], antifungal [6], antibiofilm [7], antibacterial [7], antiviral [8], antioxidant [9], antitumor [10,11], anti-HIV [12], calcium channel blocking [13], antitubercular [14] , glutamate receptor antagonistic[15], 5-HT2a receptor antagonistic [16] and group II metabotropic glutamate receptor antagonist activities [15]. Those compounds have also been reported as inhibitors of CDC25B phosphatase [17], Bcl-2 family proteins [17], and acetylcholinesterase enzymes [18].

The sulfonic acid group represents a key structural motif in both synthetic and medicinal chemistry. The phosphate functional group can be replaced by sulfonic acid moieties via bioisosteric replacement. These features are functionally interchangeable due to their ability to adopt a negative charge at biological pH values [19]. Many compounds containing sulfonic acid groups are well known as antibacterial [20,21,22,23], antifungal [24,25,26,27,28] and antitubercular agents [29]. Additionally, compounds containing sulfonic acid groups are used as dyes [30], and in metal arenesulfonate complexes [31].

**Figure 1:**
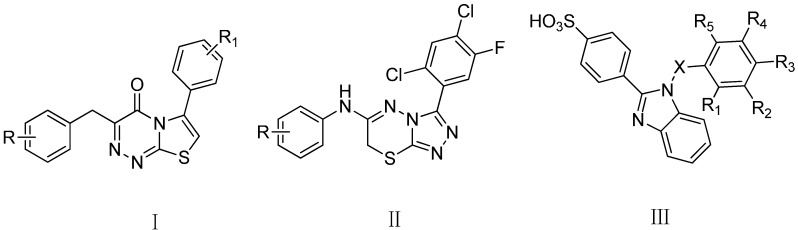
Model compounds with pharmacological activities.

It has been found that some 5*H*-thiazolo[3,2-*a*]pyrimidin-5-one structural analogues possess potent antimicrobial activity. A literature survey revealed that different halogen-substitution positions on the phenyl ring of thiazolotriazinones (**I**, Figure 1) result in a wide range of important pharmacological properties [32]. Moreover, aminotriazolothiadiazines (**II**, Figure 1) showed very good antibacterial and antifungal activities at 6.25 mg/mL concentrations [33]. On the other hand, it was reported that the introduction of a sulfonic acid group might augment the antimicrobial activity [34]. Numerous 4-(1*H*-benzoimidazol-2-yl)-benzenesulfonic acids (**III**, Figure 1) were found to be the most effective antibacterial and antifungal compounds. In addition, the role of electron-withdrawing nitro groups in increasing the antimicrobial activity was noted. 

Encouraged by the enormous pharmacological importance of 5*H*-thiazolo[3,2-*a*]pyrimidin-5-ones and the sulfonic acid motif, we focused on applying a scaffold hopping approach and developing a novel series of substituted 5*H*-thiazolo[3,2-*a*]pyrimidin-5-ones and their sulfonic acid derivatives. These compounds were subsequently evaluated for their *in vitro* antibacterial and antitubercular activity.

## 2. Results and Discussion

### 2.1. Chemistry

**Scheme 1:**
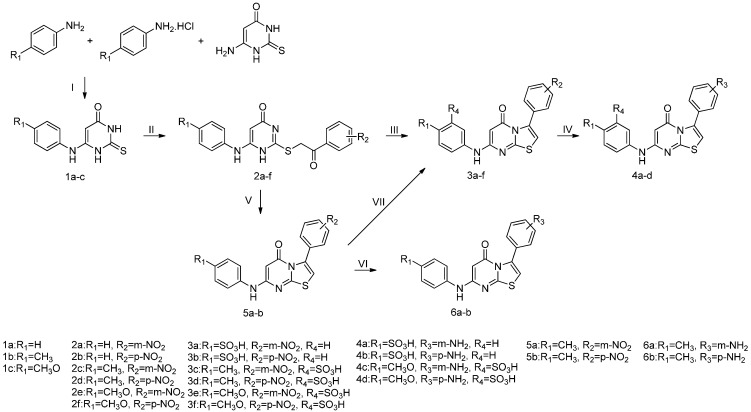
Synthesis of 5*H*-thiazolo[3,2-*a*]pyrimidin-5-one derivatives.

The synthesis of the new 5*H*-thiazolo[3,2-*a*]pyrimidin-5-ones and their derivatives containing a sulfonic acid moiety is summarized in Scheme 1.

*Reagents and Conditions*: **I**, 170 °C, reflux; **II**, K_2_CO_3_, substituted phenacyl halides, DMF; **III**, concentrated H_2_SO_4_, 20 °C or 80 °C; **IV**, Fe, NH_4_Cl, 2:1 ethanol/water, 80 °C; **V**, concentrated H_2_SO_4_, 20 °C; **VI**, Fe, NH_4_Cl, 2:1 ethanol/water, 80 °C; **VII**, concentrated H_2_SO_4_, 80 °C.

**Scheme 2:**
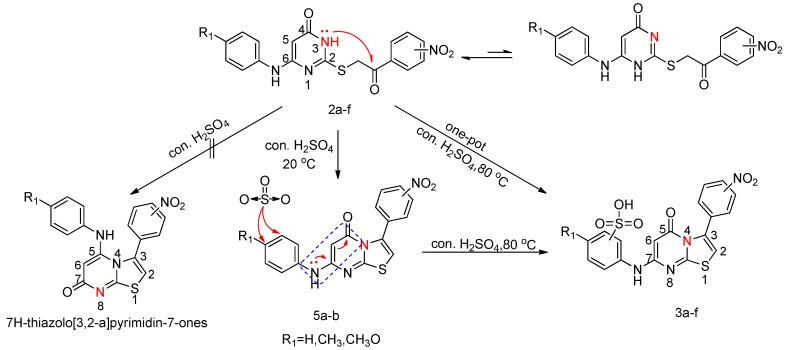
A plausible mechanism for this selective cyclization

The 6-substituted anilino-2-thiouracil starting materials **1a**–**c** were synthesized according to known procedures based on the reactions of 6-amino-2-thiouracils with substituted anilines in the presence of aniline hydrochloride at high temperature [35,36,37]. Thus, the obtained thiopyrimidines **1a**–**c** were nearly quantatitatively *S*-alkylated with the appropriate substituted phenacyl halides in the presence of anhydrous potassium carbonate [36,38]. The products **2a**–**f** could be used in subsequent reactions without further purification. The *S*-alkylated derivatives are proved to exist in solution largely in the lactam form as indicated from spectroscopic studies. The tautomeric hydrogen was found to favour N3 rather than N1 [39,40,41,42,43] (Scheme 2).

Inspection of the 400-MHz ^1^H-NMR spectra of the *S*-alkylated derivatives revealed an interesting phenomenon. The ^1^H-NMR spectrum (DMSO-*d*_6_) of compound **2a** showed the characteristic singlet at δ 5.02 ppm for the methenyl protons and at δ 5.46 ppm for the 5-H proton of pyrimidine, the 3-nitrophenyl protons appeared between δ 8.59–8.61, 8.51–8.53, 7.33–7.35 ppm, the phenyl (anilino) protons between δ 7.42–7.45, 6.99–7.03, 6.83–6.86 ppm and the NH protons (at the pyrimidine C-6 atom) around δ 8.73 ppm. The signal was observed at δ 11.96 due to the N3-H proton. Moreover, the compound **2a** seem to exist only partially in the cyclic form, The spectrum of compound **2a** had a pair of doublets centered at δ 3.78 and 3.67 ppm comprising an AB system (*J* = 12.3 Hz) [12] and two singlets at δ 5.85, 5.24 ppm for OH, but an AB quartet did not fully account for two methylene protons. In most cases tautomerism in these compounds is clearly solvent-dependent [40].

**Scheme 3:**
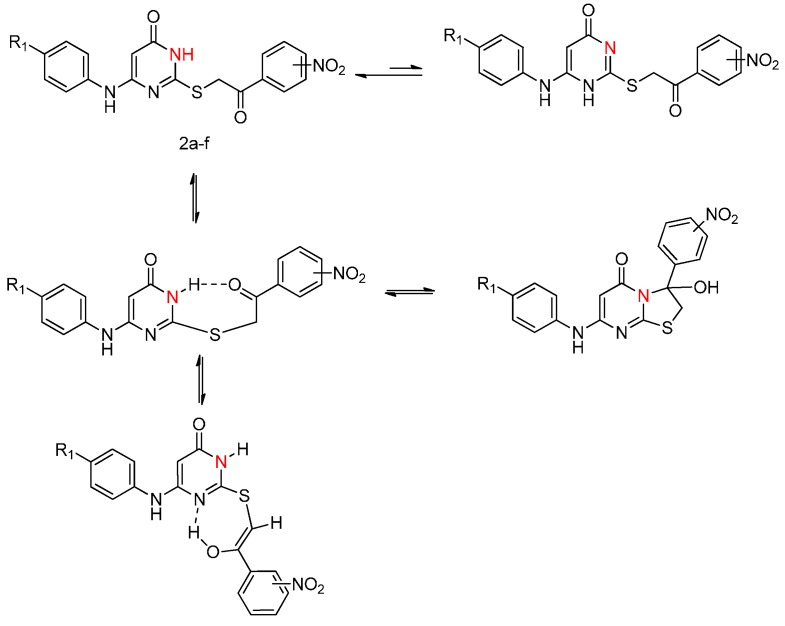
Possible interconversion of *S*-alkylated derivatives.

A similar phenomenon is also observed in other ^1^H-NMR spectra of *S*-alkylated derivatives (see Supporting Information). We interpret this effect as being due to the presence of thermally interconvertible *cis*-*trans* geometric isomers and their keto-enol tautomerism [44,45] (Scheme 3).

Cyclization of *S*-alkylated derivatives **2a**–**f** in concentrated H_2_SO_4_ represents an interesting case; mixtures of products (5*H*-thiazolo[3,2-*a*]pyrimidin-5-ones and sulfonic acid derivatives) were obtained which varied in relative amounts depending upon the reaction conditions (temperature and substituent groups).

Cyclization of **2c** and **2d** in concentrated H_2_SO_4_ at room temperature afforded 5*H*-thiazolo[3,2-*a*]pyrimidin-5-ones **5a** and **5b**, respectively, in good yields. The progress of the reaction was monitored by TLC. As can be seen, formation of aromatic sulfonic acids increased markedly with increasing reaction temperature. For example, only a negligible amount of the aromatic sulfonic acid was formed at 20 °C after 72 h. Upon raising the reaction temperature in increments, increasing amounts of the aromatic sulfonic acid were observed in the reaction mixtures by TLC. When the temperature was raised to 80 °C, the reaction with heating for 24 h gives these heteroaromatic sulfonic acid derivatives as the only products in high yield, which is attributable to the relatively weak electron-donating ability and relatively large steric hindrance of the methyl group.

In contrast, when compounds **2a**–**b**, **2e**–**f** were stirred for 72 h at 20 °C, they were converted completely into the monosulfonic acids of thienopyrimidines (TLC monitoring). Attempts to reduce the reaction times by further increasing the temperature from 20 °C to 80 °C are effective. This one-pot procedure shortened the total reaction time from 72 to 24 h.

Theoretically, the intramolecular cyclization of *S*-alkylated derivatives may afford the two possible isomeric products: 5*H*-thiazolo[3,2-*a*]pyrimidin-5-ones and 7*H*-thiazolo[3,2-*a*]pyrimidin-7-ones. Formation of these isomers may be explained on the basis of nucleophilicity differences of N1 and N3 position of *S*-alkylated derivatives. However, in practice cyclization of *S*-alkylated derivatives **2a**–**f** were found to afford only the corresponding 5*H*-thiazolo[3,2-*a*]pyrimidin-5-ones.

The regioselectivity of the cyclization step, maybe due to a difference in the electron density at the N1 and N3 positions of 3,4-dihydropyrimidine-2(1*H*)-thione. The higher electron density of the N3 atom resulted in exclusive cyclization at this position [46] (Scheme 2). In addition, theoretical computations also reveal that the regioisomers 5-ones resulting from the N3 intramolecular cyclization are more stable and form the major regioisomer [47]. Moreover, 5*H*-thiazolo[3,2-*a*]pyrimidin-5-ones were formed as a result of intramolecular cyclization through nucleophilic attack of the pyrimidine N3, onto the phenacyl carbonyl carbon. The selective C2-N3 annulation is due to the steric repulsions between the aryl group at position 6 of the keto sulfides and carbonyl group [40].

Compounds **3a**–**f** were obtained by one-pot cyclization and sulfonation of **2a**–**f***.* These conditions were favorable for the introduction of one sulfonic acid group and avoided undesirable oversulfonation. When compounds **2a**–**b** undergo this one-pot procedure, sulfonation occurs solely at the electronically favored positions which are *para* to the amino groups to give the compounds **3a**–**b**. For steric reasons, sulfonation of the phenyl ring did not afforded *ortho*-sulfonic acids. Thus, for compounds **2c**–**f**, two substituents already present of the phenyl ring have a direct effect the introduction of the sulfonic acid group, as there are only two possible sulfonate isomers that can be formed. *N*-phenyl substitution of products 5*H*-thiazolo[3,2-*a*]pyrimidin-5-ones could lead to an amino conjugation effect of the cyclic α,β-unsaturated ketone of 5*H*-thiazolo[3,2-*a*]pyrimidin-5-ones (Scheme 2), which simultaneously decreases the conjugation on the phenyl ring. When a third substituent is introduced into the phenyl ring of 5*H*-thiazolo[3,2-*a*]pyrimidin-5-ones, both NH and R_1_ (electron-donating groups) exert an influence, but the group R_1_ whose influence predominates directs the sulfonic acid group to the place it will occupy. We find that compounds with the new substituent in the *ortho-*positions relative to R_1_ are obtained exclusively. Another explanation of this phenomenon might be some steric influence of the 5*H*-thiazolo[3,2-*a*]pyrimidin-5-one nucleus. Similar conclusions regarding the regioselectivity of sulfonation of the phenyl ring have also been reported in the literature [48,49,50,51,52].

### 2.2. Biological Assays

All of the synthesized compounds were evaluated *in vitro* using a broth micro dilution method to obtain their minimum inhibitory concentration (MIC) values against two Gram-positive bacterial strains: *Staphylococcus aureus* (*S. aureus*), *Bacillus subtilis* (*B. subtilis*); two Gram-negative bacterial strains: *Escherichia coli* (*E. coli*), *Pseudomonas aeruginosa* (*P. aeruginosa*) and *Mycobacterium smegmatis (M. smegmatis).* The MIC values of these compounds were presented in Table 1. 

**Table 1:** *In vitro* antibacterial and antitubercular activity.

Compd. No.	Antibacterial Activity MIC (μg/mL)				Antitubercular Activity MIC (μg/mL)
	*S. aureus*	*B. subtilis*	*E. coli*	*P. aeruginosa*	*M. smegmatis*
**3a**	200	400	50	100	-
**3b**	100	200	100	200	-
**3c**	200	200	100	100	-
**3d**	200	400	100	100	-
**3e**	400	800	200	200	-
**3f**	400	800	100	200	-
**4a**	400	800	400	400	100
**4b**	400	800	200	400	100
**4c**	800	-	400	-	50
**4d**	-	-	400	-	50
**5a**	100	100	100	100	-
**5b**	50	100	50	50	-
**6a**	100	100	100	400	800
**6b**	200	200	200	400	-
**CIP**	25	100	25	50	/
**RIP**	/	/	/	/	25

“-”Indicates bacteria is resistant to the compounds at >800 µg/mL. MIC (µg/mL) = lowest concentration to completely inhibit bacterial growth. Reference drugs: CIP, Ciprofloxacin; RIP, Rifampicinn.

Examination of the antibacterial screening data reveals that all the tested compounds display significant antibacterial activity against Gram-negative bacteria and moderate activity against Gram-positive bacteria. In general, compounds having nitro substituents displayed significant inhibitory activity, that was only slightly affected by the nitro substituent being located on the 3- or 4-position of the phenyl group. In addition, compounds without sulfonic group had better antibacterial than the corresponding compounds with sulfonic acid groups, which could be seen from compounds **5a**, **5b** that possess the highest antibacterial activities. From first examination of the antitubercular activity results, it appears that compounds **4a**–**d**, containing an amino group, show better activity against *M. smegmatis* and compounds **4c**, **4d** showed the highest activity (MIC 50 µg /mL). This may be due to the influence of the methoxy substituent.

## 3. Experimental Section 

### 3.1. General Information

Melting points were determined in open capillary tubes with a WRS-1B melting point apparatus (Shanghai Shenguang Instrnment Co., Ltd, Shanghai, China) and are uncorrected. IR spectra (KBr) were recorded on a FTIR920 spectrophotometer (Tianjin Tuopu Instrument Co., Ltd., Tianjin, China). The ^1^H- and ^13^C-NMR spectra were obtained from a solution in DMSO-*d*_6_ with TMS as internal standard using a 400/101 MHz (^1^H-/^13^C-) spectrometer (Agilent Technologies, Santa Clara, CA, USA). Mass spectra were acquired from an Agilent 6200 Series TOF and 6500 Series Q-TOF LC/MS System B.05.01. (B5125, Agilent Technologies, Santa Clara, CA, USA).

### 3.2. Synthesis

#### 3.2.1. General Procedure for the Synthesis of **1a**–**c**

A mixture of 6-amino-2-thiouracil (50 mmol), an appropriate aniline (100 mmol) together with anilinium chloride (75–100 mmol) was heated at 175 °C for 7–12 h. The warm mixture was diluted with 65% ethanol (200 mL) and cooled. The precipitate was filtered and washed with cold ethanol, which was dissolved in hot 5% NaOH solution, and the filtrate was neutralized with 10% HCl to get more pure product. The solid deposited was filtered, washed with water, dried, and crystallized from a large volume of CH_3_OH to yield the title compounds.

*6-(Phenylamino)-2-thioxo-2,3-dihydropyrimidin-4(1H)-one* (**1a**): White solid; Yield, 79.3%; m.p.: 266.5–267.8 °C (lit. [36,53] 287–288 and 281–283 °C); HRMS (*m*/*z*): calcd. for C_10_H_9_N_3_OS (neutral M + H) 220.0545, found 220.0553.

*2-Thioxo-6-(p-tolylamino)-2,3-dihydropyrimidin-4(1H)-one* (**1b**): White solid; Yield, 91.8%; m.p.: 256.6–257.1 °C (lit. [35] 293–295 °C, decomp., from DMF–H_2_O); HRMS (*m*/*z*): calcd. for C_11_H_11_N_3_OS (neutral M + H) 234.0701, found 234.0719.

*6-((4-Methoxyphenyl)amino)-2-thioxo-2,3-dihydropyrimidin-4(1H)-one* (**1c**): White solid; Yield, 94.1%; m.p.: 269.7–270.1 °C (lit. [35] 284–286 °C, decomp., from DMF–H_2_O); HRMS (*m*/*z*): calcd. for C_11_H_11_N_3_O_2_S (neutral M + H) 250.0650, found 250.0661.

#### 3.2.2. General Procedure for the Synthesis of Compounds **2a**–**f**

Anhydrous potassium carbonate (10 mmol) and substituted phenacyl halides (10 mmol) were added in succession to a suspension of 6-substituted-2-thiouracil **1** (12 mmol) in dry *N,N*-dimethylformamide (10 mL). After stirring for 3 h at room temperature, the mixture was quenched with water (100 mL) and filtered. The residues were purified by crystallization to give compounds **2a**–**f**.

*2-((2-(3-Nitrophenyl)-2-oxoethyl)thio)-6-(phenylamino)pyrimidin-4(3H)-one* (**2a**): Yellow solid; Yield, 80.3%; m.p.: 222.3–223.0 °C; HRMS (*m*/*z*): calcd. for C_18_H_14_N_4_O_4_S (neutral M + H) 383.0814, found 383.0837.

*2-((2-(4-Nitrophenyl)-2-oxoethyl)thio)-6-(phenylamino)pyrimidin-4(3H)-one* (**2b**): Yellow solid; Yield, 82.8%; m.p.: 218.3–219.5 °C; HRMS (*m*/*z*): calcd. for C_18_H_14_N_4_O_4_S (neutral M + H) 383.0814, found 383.0837.

*2-((2-(3-Nitrophenyl)-2-oxoethyl)thio)-6-(p-tolylamino)pyrimidin-4(3H)-one* (**2c**): Yellow solid; Yield, 83.2%; m.p.: 222.1–224.8 °C; HRMS (*m*/*z*): calcd. for C_19_H_16_N_4_O_4_S (neutral M+H) 397.0971, found 397.0997.

*2-((2-(4-Nitrophenyl)-2-oxoethyl)thio)-6-(p-tolylamino)pyrimidin-4(3H)-one* (**2d**): Yellow solid; Yield, 83.9%; M.p.: 227.6–228.6 °C; HRMS (m/z): calcd for C_19_H_16_N_4_O_4_S (neutral M+H) 397.0971, found 397.0986.

*6-((4-Methoxyphenyl)amino)-2-((2-(3-nitrophenyl)-2-oxoethyl)thio)pyrimidin-4(3H)-one* (**2e**): Yellow solid; Yield, 79.6%; m.p.: 217.1–217.3 °C; HRMS (*m*/*z*): calcd. for C_19_H_16_N_4_O_5_S (neutral M + H) 413.0920, found 413.0951.

*6-((4-Methoxyphenyl)amino)-2-((2-(4-nitrophenyl)-2-oxoethyl)thio)pyrimidin-4(3H)-one* (**2f**): Yellow solid; Yield, 78.5%; m.p.: 215.8–217.7 °C; HRMS (*m*/*z*): calcd. for C_19_H_16_N_4_O_5_S (neutral M + H) 413.0920, found 413.0948.

#### 3.2.3. General Procedure for the Synthesis of Compounds **3a**–**f**

*S*-alkylated derivatives **2** (1 mmol) were carefully dissolved in concentrated sulfuric acid (7.5 mL) and heated in an oil bath at 80 °C for 24 h. After cooling, The reaction mixture was carefully poured into ethyl acetate (about 50 mL), to form a precipitate which was collected, washed with ethyl acetate and dried. The crude product was recrystallized from ethyl acetate to give **3a**–**f**.

*4-((3-(3-Nitrophenyl)-5-oxo-5H-thiazolo[3,2-a]pyrimidin-7-yl)amino)benzenesulfonic acid* (**3a**): Yellow solid; Yield, 91.7%; m.p.: (decomp.) 257.6 °C; IR (ν_max_/cm^−1^): 3398, 3099, 1570, 1520, 1345, 1192, 1129, 1043, 821, 734; ^1^H-NMR δ 9.50 (s, 1H), 8.33–8.22 (m, 2H), 7.90 (dt, *J* = 7.8, 1.4 Hz, 1H), 7.67 (t, *J* = 7.8 Hz, 1H), 7.62–7.54 (m, 2H), 7.47–7.33 (m, 3H), 5.38 (d, *J* = 1.2 Hz, 1H); ^13^C-NMR δ 164.73, 159.54, 158.98, 146.90, 143.13, 140.14, 136.07, 135.48, 134.13, 128.87, 126.82, 124.42, 123.39, 119.89, 111.26, 82.66; HRMS (*m*/*z*): calcd. for C_18_H_12_N_4_O_6_S_2_ (neutral M + H) 445.0277, found 445.0310.

*4-((3-(4-Nitrophenyl)-5-oxo-5H-thiazolo[3,2-a]pyrimidin-7-yl)amino)benzenesulfonic acid* (**3b**): Yellow solid; Yield, 92.4%; m.p.: (decomp.) 210.0 °C; IR (ν_max_/cm^−1^): 3442, 3284, 3109, 1671, 1568, 1520, 1350, 1232, 1134, 1031, 1000, 836, 750; ^1^H-NMR δ 9.50 (s, 1H), 8.31–8.13 (m, 2H), 7.76–7.67 (m, 2H), 7.60–7.53 (m, 2H), 7.48–7.32 (m, 3H), 5.38 (s, 1H); ^13^C-NMR δ 164.73, 159.36, 158.98, 147.33, 143.29, 140.02, 139.02, 135.68, 130.74, 126.81, 122.49, 119.93, 111.75, 82.52; HRMS (*m*/*z*): calcd. for C_18_H_12_N_4_O_6_S_2_ (neutral M + H) 445.0277, found 445.0313.

*2-Methyl-5-((3-(3-nitrophenyl)-5-oxo-5H-thiazolo[3,2-a]pyrimidin-7-yl)amino)benzenesulfonic acid* (**3c**): Yellow solid; Yield, 90.6%; m.p.: (decomp.) 244.5–249.8 °C; IR (ν_max_/cm^−1^): 3430, 3267, 3089, 1642, 1588, 1527, 1347, 1218, 1162, 1086, 1019, 810; ^1^H-NMR δ 9.36 (s, 1H), 8.30–8.27 (m, 2H), 7.90 (dt, *J* = 7.8, 1.4 Hz, 1H), 7.68 (q, *J* = 7.6 Hz, 1H), 7.63–7.44 (m, 2H), 7.37 (s, 1H), 7.19 (dd, *J* = 8.2, 2.1 Hz, 1H), 5.44 (s, 1H), 2.29 (s, 3H); ^13^C-NMR δ 164.42, 159.74, 158.13, 146.91, 137.31, 136.05, 135.39, 134.05, 133.54, 131.69, 130.50, 128.86, 128.09, 124.37, 123.37, 120.84, 111.17, 81.98, 20.82; HRMS (*m*/*z*): calcd. for C_19_H_14_N_4_O_6_S_2_ (neutral M + H) 459.0433, found 458.9985.

*2-Methyl-5-((3-(4-nitrophenyl)-5-oxo-5H-thiazolo[3,2-a]pyrimidin-7-yl)amino)benzenesulfonic acid* (**3d**): Yellow solid; Yield, 88.9%; m.p.: (decomp.) 263.7 °C; IR (ν_max_/cm^−1^): 3366, 3110, 2956, 1653, 1601, 1515, 1345, 1176, 1091, 1026, 822; ^1^H-NMR δ 9.37 (s, 1H), 8.32 (d, *J* = 8.6 Hz, 1H), 8.22 (d, *J* = 8.4 Hz, 2H), 7.71 (d, *J* = 8.4 Hz, 2H), 7.55 (d, *J* = 6.1 Hz, 1H), 7.38 (s, 1H), 7.20 (d, *J* = 8.4 Hz, 1H), 5.45 (s, 1H), 2.29 (s, 3H); ^13^C-NMR δ 164.44, 159.60, 158.13, 147.31, 138.93, 135.60, 133.51, 131.73, 130.71, 128.10, 126.76, 124.61, 122.50, 120.83, 111.72, 81.91, 21.45; HRMS (*m*/*z*): calcd. for C_19_H_14_N_4_O_6_S_2_ (neutral M + H) 459.0433, found 459.0463.

*2-Methoxy-5-((3-(3-nitrophenyl)-5-oxo-5H-thiazolo[3,2-a]pyrimidin-7-yl)amino)benzenesulfonic acid* (**3e**): Yellow solid; Yield, 78.5%; m.p.: (decomp.) 290.3–293.4 °C; IR (ν_max_/cm^−1^): 3398, 3096, 1673, 1618, 1570, 1526, 1496, 1448, 1347, 1194, 1091, 1034, 812; ^1^H-NMR δ 9.24 (s, 1H), 8.32–8.21 (m, 2H), 7.89 (dd, *J* = 7.8, 1.4 Hz, 1H), 7.72–7.61 (m, 2H), 7.44–7.36 (m, 1H), 7.33 (d, *J* = 1.2 Hz, 1H), 6.99 (dd, *J* = 8.8, 1.2 Hz, 1H), 5.17 (d, *J* = 1.2 Hz, 1H), 3.77 (d, *J* = 1.3 Hz, 3H); ^13^C-NMR δ 164.68, 159.87, 159.55, 153.12, 146.93, 136.38, 136.13, 135.50, 134.25, 131.19, 128.86, 124.43, 124.34, 123.37, 123.26, 112.92, 110.70, 80.90, 56.25; HRMS (*m*/*z*): calcd. for C_19_H_14_N_4_O_7_S_2_ (neutral M + H) 475.0382, found 475.0422.

*2-Methoxy-5-((3-(4-nitrophenyl)-5-oxo-5H-thiazolo[3,2-a]pyrimidin-7-yl)amino)benzenesulfonic acid* (**3f**): Yellow solid; Yield, 80.4%; m.p.: (decomp.) 272.1 °C; IR (ν_max_/cm^−1^): 3373, 3088, 2947, 1709, 1632, 1606, 1570, 1490, 1233, 1200, 1088, 1023, 853; ^1^H-NMR δ 9.25 (s, 1H), 8.21 (d, *J* = 8.4 Hz, 2H), 7.70 (d, *J* = 8.9 Hz, 3H), 7.39 (d, *J* = 10.5 Hz, 1H), 7.34 (d, *J* = 0.7 Hz, 1H), 6.99 (d, *J* = 8.7 Hz, 1H), 5.17 (s, 1H), 3.75 (s, 3H); ^13^C-NMR δ 164.69, 159.86, 159.41, 153.25, 147.31, 139.12, 136.25, 135.71, 130.74, 126.95, 125.35, 124.73, 123.32, 113.00, 111.26, 80.78, 56.16; HRMS (*m*/*z*): calcd. for C_19_H_14_N_4_O_7_S_2_ (neutral M + H) 475.0382, found 475.0424.

#### 3.2.4. General Procedure for the Synthesis of Compounds **4a**–**d**

A suspension of nitro compounds **3a**–**f** (3.53 mmol) in ethanol (60 mL) and water (30 mL) was treated with ammonium chloride (3.53 mmol) and iron powder (17.65 mmol). After being stirred at 80 °C for 2 h, the mixture was diluted with ethanol (40 mL) and filtered through diatomaceous earth (Celite^®^) while hot. The filtrant was washed with hot ethanol, and the filtrate was concentrated. The crude product was purified by column chromatography on silica gel using petroleum CH_2_Cl_2_/CH_3_OH as eluent to afford the pure products.

*4-((3-(3-Aminophenyl)-5-oxo-5H-thiazolo[3,2-a]pyrimidin-7-yl)amino)benzenesulfonic acid* (**4a**): Gray solid; Yield, 62.7%; m.p: (decomp.) 257.6 °C; IR (ν_max_/cm^−1^): 3457, 3263, 3093, 2998, 1669, 1592, 1568, 1498, 1446, 1391, 1321, 1187, 1121, 1030, 818, 710; ^1^H-NMR δ 9.42 (s, 1H), 7.60–7.54 (m, 2H), 7.40 (d, *J* = 8.2 Hz, 2H), 7.07–6.97 (m, 2H), 6.65–6.54 (m, 3H), 5.50 (s, 2H), 5.35 (s, 1H); ^13^C-NMR δ 164.95, 159.21, 158.72, 147.10, 142.91, 140.29, 138.80, 133.27, 128.06, 126.78, 119.78, 117.85, 115.48, 114.68, 108.44, 82.72; HRMS (*m*/*z*): calcd. for C_18_H_14_N_4_O_4_S_2_ (neutral M + H) 415.0535, found 415.0524.

*4-((3-(4-Aminophenyl)-5-oxo-5H-thiazolo[3,2-a]pyrimidin-7-yl)amino)benzenesulfonic acid* (**4b**): Gray solid; Yield, 65.8%; m.p.: (decomp.) 246.8 °C; IR (ν_max_/cm^−1^): 3397, 3100, 1676, 1625, 1568, 1506, 1453, 1328, 1270, 1189, 1124, 1038, 830, 712; ^1^H-NMR δ 9.40 (s, 1H), 7.58 (d, *J* = 7.9 Hz, 2H), 7.40 (d, *J* = 7.6 Hz, 2H), 7.05 (d, *J* = 7.9 Hz, 2H), 6.91 (s, 1H), 6.52 (d, *J* = 8.0 Hz, 2H), 5.44 (s, 2H), 5.35 (s, 1H); ^13^C-NMR δ 165.04, 159.62, 158.56, 149.26, 142.70, 140.42, 139.50, 130.46, 126.81, 119.87, 119.69, 112.56, 106.51, 82.88; HRMS (*m*/*z*): calcd. for C_18_H_14_N_4_O_4_S_2_ (neutral M + H) 415.0535, found 415.0545.

*5-((3-(3-Aminophenyl)-5-oxo-5H-thiazolo[3,2-a]pyrimidin-7-yl)amino)-2-methoxybenzenesulfonic acid* (**4c**): Yellow solid; Yield, 58.9%; m.p.: (decomp.) 181.5 °C; IR (ν_max_/cm^−1^): 3368, 3279, 1661, 1585, 1500, 1436, 1192, 1083, 1028, 815, 700; ^1^H-NMR δ 9.11 (s, 1H), 7.66 (s, 1H), 7.34 (d, *J* = 8.3 Hz, 1H), 6.96 (d, *J* = 6.9 Hz, 3H), 6.53 (q, *J* = 8.4, 7.1 Hz, 3H), 5.14 (s, 2H), 5.11 (s, 1H), 3.73 (s, 3H); ^13^C-NMR δ 164.88, 159.62, 159.26, 153.19, 147.89, 138.90, 133.27, 131.18, 128.02, 124.42, 123.32, 117.12, 115.06, 114.22, 112.95, 107.72, 80.80, 56.16; HRMS (*m*/*z*): calcd. for C_19_H_16_N_4_O_5_S_2_ (neutral M + H) 445.0640, found 445.0627.

*5-((3-(4-Aminophenyl)-5-oxo-5H-thiazolo[3,2-a]pyrimidin-7-yl)amino)-2-methoxybenzenesulfonic acid* (**4d**): Yellow solid; Yield, 56.8%; m.p.: (decomp.) 288.6 °C; IR (ν_max_/cm^−1^): 3344, 3030, 2971, 1686, 1618, 1567, 1489, 1440, 1333, 1274, 1194, 1087, 877, 818; ^1^H-NMR δ 9.12 (s, 1H), 7.67 (d, *J* = 2.8 Hz, 1H), 7.39 (dd, *J* = 8.7, 2.8 Hz, 1H), 7.01 (dd, *J* = 24.2, 8.5 Hz, 3H), 6.85 (s, 1H), 6.52 (d, *J* = 8.3 Hz, 2H), 5.43 (s, 2H), 5.14 (s, 1H), 3.76 (s, 3H); ^13^C-NMR δ 164.99, 159.64, 159.46, 152.94, 149.03, 139.45, 136.18, 131.37, 130.42, 124.35, 123.06, 120.13, 112.90, 112.67, 105.97, 81.05, 56.23; HRMS (*m*/*z*): calcd. for C_19_H_16_N_4_O_5_S_2_ (neutral M + H) 445.0640, found 445.0646.

#### 3.2.5. General Procedure for the Synthesis of Compounds **5a**–**b**

*S*-alkylated derivatives **2** (1 mmol) were carefully dissolved in concentrated sulfuric acid (7.5 mL) and stirred at 20 °C for 72 h. After cooling, it was carefully poured into water (about 50 mL), precipitation which was collected, washed with cold water and dried. The crude product was recrystallized from ethyl acetate to give products.

*3-(4-Nitrophenyl)-7-(p-tolylamino)-5H-thiazolo[3,2-a]pyrimidin-5-one* (**5a**): Yellow solid; Yield, 94.5%; m.p.:143.2–144.9 °C; IR (ν_max_/cm^−1^): 3389, 3107, 1662, 1609, 1568, 1518, 1343, 1134, 1022, 807, 736; ^1^H-NMR δ 9.31 (s, 1H), 8.32–8.22 (m, 2H), 7.89 (dq, *J* = 7.6, 1.4 Hz, 1H), 7.67 (td, *J* = 7.9, 1.4 Hz, 1H), 7.35 (d, *J* = 1.6 Hz, 1H), 7.34–7.29 (m, 2H), 7.17 (d, *J* = 7.9 Hz, 2H), 5.28 (d, *J* = 1.5 Hz, 1H), 2.29 (s, 3H); ^13^C-NMR δ 164.65, 159.52, 146.89, 142.41, 137.22, 136.03, 135.48, 134.16, 132.71, 129.87, 128.86, 124.40, 123.36, 121.68, 110.87, 81.61, 20.90; HRMS (*m*/*z*): calcd. for C_19_H_14_N_4_O_3_S (neutral M + H) 379.0865, found 379.0892.

*3-(4-Nitrophenyl)-7-(p-tolylamino)-5H-thiazolo[3,2-a]pyrimidin-5-one* (**5b**): Yellow solid; Yield, 96.4%; m.p.: 143.8–145.1 °C; IR (ν_max_/cm^−1^): 3297, 3105, 2923, 1664, 1604, 1515, 1345, 1197, 1091, 820; ^1^H-NMR δ 9.33 (s, 1H), 8.22 (d, *J* = 8.4 Hz, 2H), 7.69 (d, *J* = 8.2 Hz, 2H), 7.36 (s, 1H), 7.31 (d, *J* = 8.0 Hz, 2H), 7.17 (d, *J* = 8.0 Hz, 2H), 5.28 (s, 1H), 2.29 (s, 3H); ^13^C-NMR δ 164.66, 159.39, 159.37, 147.31, 139.04, 137.18, 135.68, 132.76, 130.71, 129.87, 122.49, 121.71, 111.42, 81.52, 20.90; HRMS (*m*/*z*): calcd. for C_19_H_14_N_4_O_3_S (neutral M + H) 379.0865, found 379.0897.

#### 3.2.6. General Procedure for the Synthesis of Compounds **6a**–**b**

A suspension of nitro compound **5a**–**b** (3.53 mmol) in ethanol (60 mL) and water (30 mL) was treated with ammonium chloride (3.53 mmol) and iron powder (17.65 mmol). After being stirred at 80 °C for 2 h, the mixture was diluted with ethanol (40 mL) and filtered through diatomaceous earth (Celite^®^) while hot. The filtrant was washed with hot ethanol, and the filtrate was concentrated, partitioned between water and ethyl acetate and the aqueous phase was extracted three times with ethyl acetate. The combined extracts were washed with brine and dried (Na_2_SO_4_), filtered and concentrated to provide the products. 

*3-(3-Aminophenyl)-7-(p-tolylamino)-5H-thiazolo[3,2-a]pyrimidin-5-one* (**6a**): Yellow solid; Yield, 96.5%; m.p. 220.9–221.7 °C; IR (ν_max_/cm^−1^): 3541, 3384, 3111, 3025, 1664, 1615, 1570, 1515, 1400, 1324, 1271, 1190, 1126, 1020, 810, 738; ^1^H-NMR δ 9.22 (s, 1H), 7.30 (d, *J* = 8.0 Hz, 2H), 7.16 (d, *J* = 8.1 Hz, 2H), 7.05–6.94 (m, 2H), 6.63–6.49 (m, 3H), 5.28 (s, 2H), 5.25 (d, *J* = 1.2 Hz, 1H), 2.28 (s, 3H); ^13^C-NMR δ 164.66, 159.52, 159.40, 146.90, 137.23, 136.03, 135.48, 134.16, 132.72, 129.87, 128.86, 124.41, 123.37, 121.69, 110.87, 81.61, 20.90; HRMS (*m*/*z*): calcd. for C_19_H_16_N_4_OS (neutral M + H) 349.1123, found 349.1112.

*3-(4-Aminophenyl)-7-(p-tolylamino)-5H-thiazolo[3,2-a]pyrimidin-5-one* (**6b**): Brown solid; Yield, 78%; m.p.: 241.2–242.8 °C; IR (ν_max_/cm^−1^): 3469, 3378, 3107, 3021, 1659, 1613, 1563, 1510, 1395, 1323, 1268, 1184, 1122, 1017, 819; ^1^H-NMR δ 9.19 (s, 1H), 7.30 (d, *J* = 8.0 Hz, 2H), 7.15 (d, *J* = 8.1 Hz, 2H), 7.08–7.01 (m, 2H), 6.87 (d, *J* = 1.2 Hz, 1H), 6.57–6.45 (m, 2H), 5.34 (s, 2H), 5.25 (d, *J* = 1.2 Hz, 1H), 2.28 (s, 3H); ^13^C-NMR δ 164.98, 159.62, 159.02, 149.31, 139.51, 137.41, 132.44, 130.44, 129.82, 121.53, 119.89, 112.51, 106.09, 81.80, 20.89; HRMS (*m*/*z*): calcd. for C_19_H_16_N_4_OS (neutral M + H) 349.1123, found 349.1128.

### 3.3. Bioassays

The standard strains were the obtained from National Center for Medical Culture Collection and China General Microbiological Culture Collection Center. The antibacterial and antitubercular activity of the synthesized compounds was performed by broth micro dilution method against the following standard bacterial strains: *Escherichia coli* [CMCC (B) 44102], *Pseudomonas aeruginosa* [CMCC (B) 10104], *Staphylococcus aureus* [CMCC (B) 26003], *Bacillus subtilis* [CMCC (B) 63501] and *M. smegmatis* [CGMCC 1.2621].

The antibacterial and antitubercular activities of the synthesized compounds were tested by the broth micro dilution method. The 2-fold diluted compounds in Mueller Hinton broth were dispensed into 96-well microtiter plates (200 μL/well), and then an aliquot of 5 × 10^5^ colony forming units (cfu)/mL of bacterial culture was added to each well (200 μL/well) to final concentrations in a range of 1–800 μg/mL. After incubating at 37 °C for 24 h, the lowest concentration without any colony growth was recorded as the MIC value. The tested compounds and reference drugs were dissolved in MeOH to get a solution and MeOH showed no inhibition zones. The resulting values were compared with the value for a reference control (ciprofloxacin in a range of 3.125–200 μg/mL was used as a reference for antibacterial activity, and rifampicin in a range of 3.125–100 μg/mL was used as a reference for antitubercular activity) under the same conditions. 

## 4. Conclusions 

In summary, a series of 7-(substituted phenylamino)-5*H*-thiazolo[3,2-*a*]pyrimidin-5-ones and sulfonated cyclized products were designed, synthesized and evaluated for antibacterial and antitubercular activities in this study. An efficient synthetic method led to 5*H*-thiazolo[3,2-*a*]pyrimidin-5-ones or the corresponding sulfonic acid derivatives at different temperatures in high yield and purity. During our extensive literature survey it was found that N3 of substituted pyrimidines was the cyclization site when *S*-alkylated derivatives was utilized to give 5*H*-thiazolo[3,2-*a*]pyrimidin-5-ones. Our results reveal that compounds having nitro substituents displayed significant antibacterial inhibitory activity, while compounds containing the amino group, show better activity against *M. smegmatis*. Further structural modification could be performed to improve the bioactivity. We believe that these compounds can be developed into potential class of antimicrobial and antitubercular agents.

## Supplementary Material

Supplementary materials can be accessed at: http://www.mdpi.com/1420-3049/20/09/16419/s1.
