# Supplementary material for: Synthesis, Antibacterial and Antitubercular Activities of Some 5H-Thiazolo[3,2-a]pyrimidin-5-ones and Sulfonic Acid Derivatives
Source: Molecules. 2015 Sep 10;20(9):16419–34. doi: 10.3390/molecules200916419 (PMC6332143; doi:10.3390/molecules200916419)
Supplement: Supplementary file 1 [file molecules-20-16419-s001.pdf]

Supporting Information

Contents:

NMR Spectra **S1–S17**

HRMS Spectra **S18–S46**

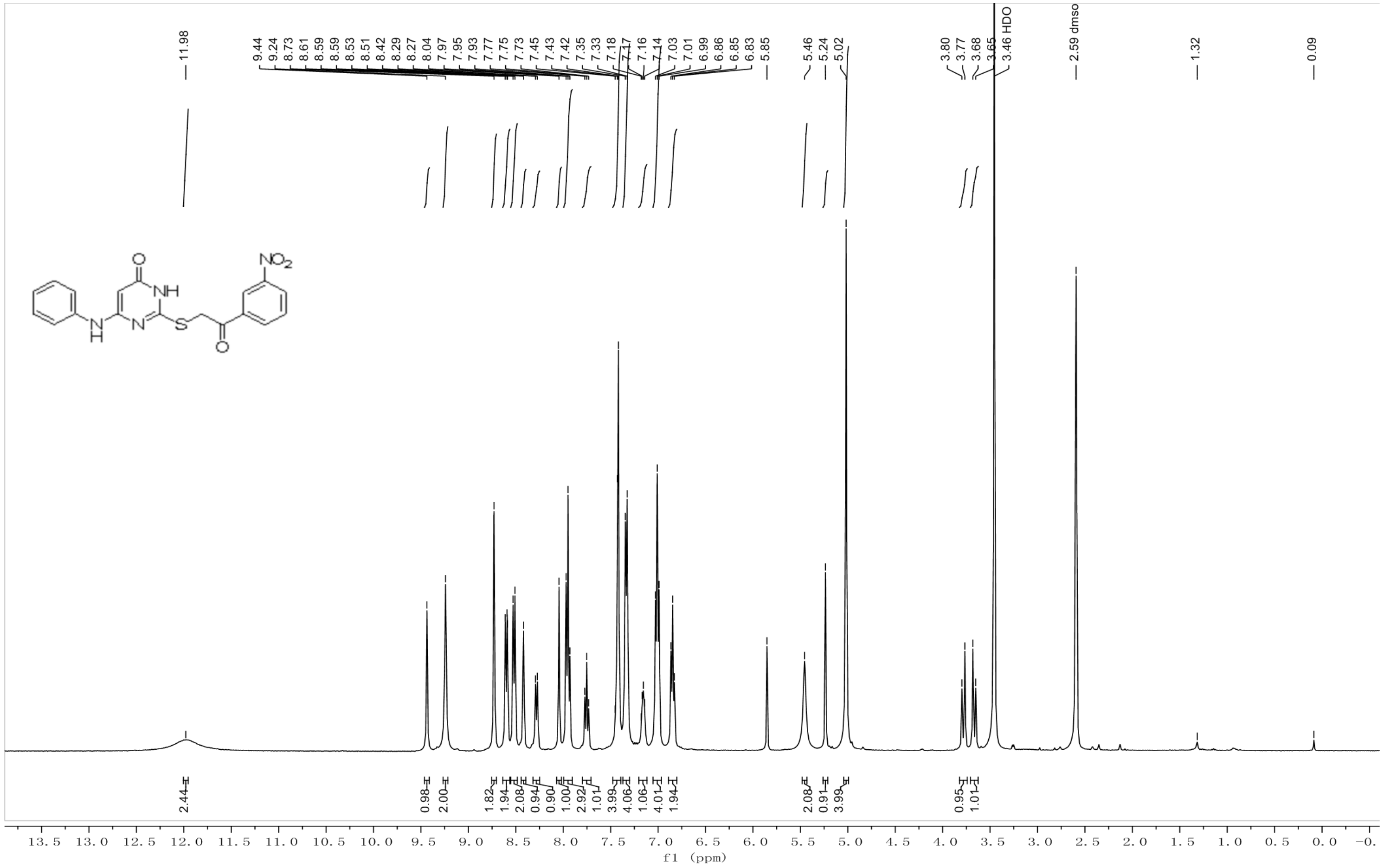

Figure S1. The NMR spectra of **2a**: 2-((2-(3-Nitrophenyl)-2-oxoethyl)thio)-6-(phenylamino)pyrimidin-4(3*H*)-one.

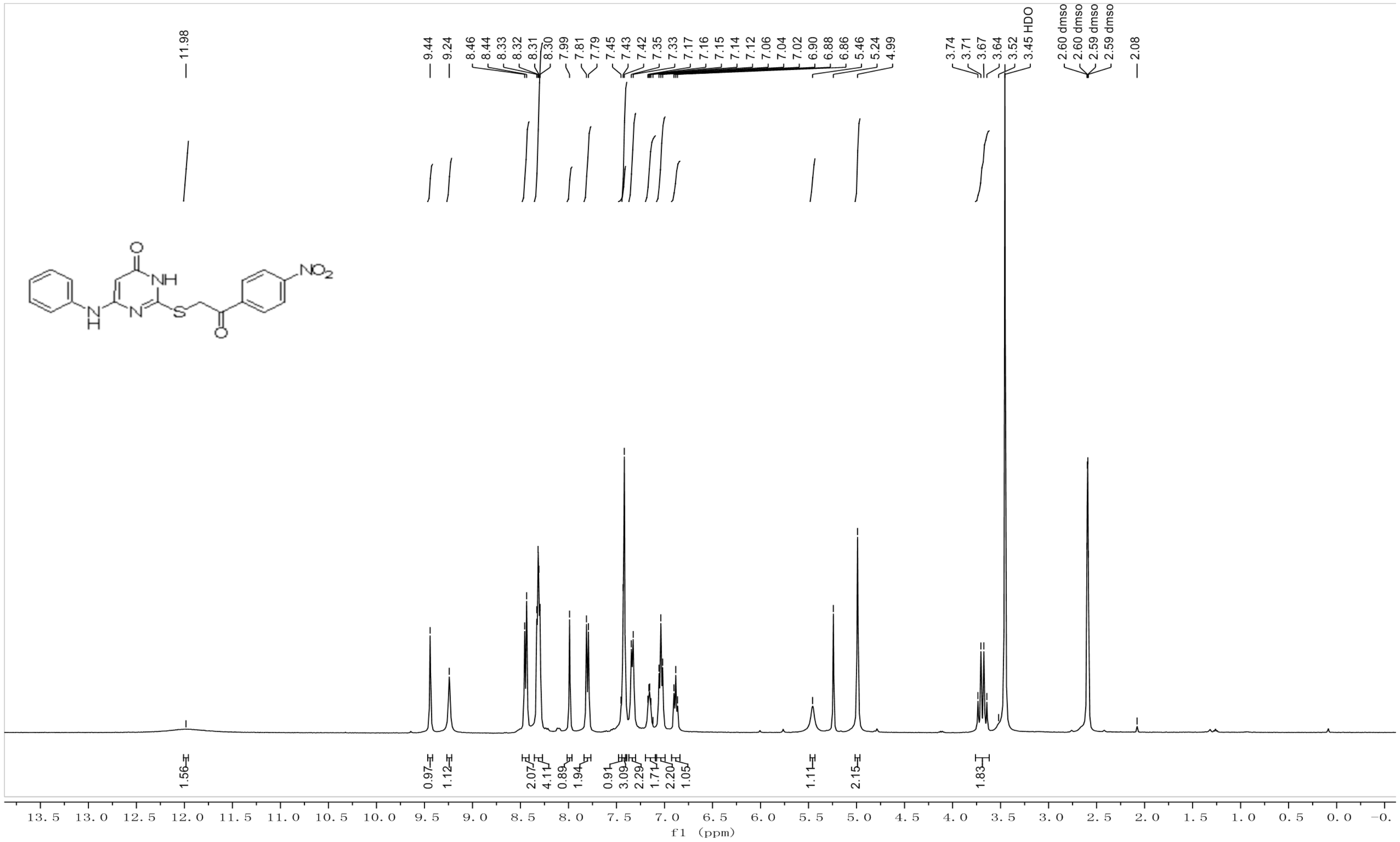

Figure S2. The NMR spectra of **2b**: 2-((2-(4-Nitrophenyl)-2-oxoethyl)thio)-6-(phenylamino)pyrimidin-4(3*H*)-one.

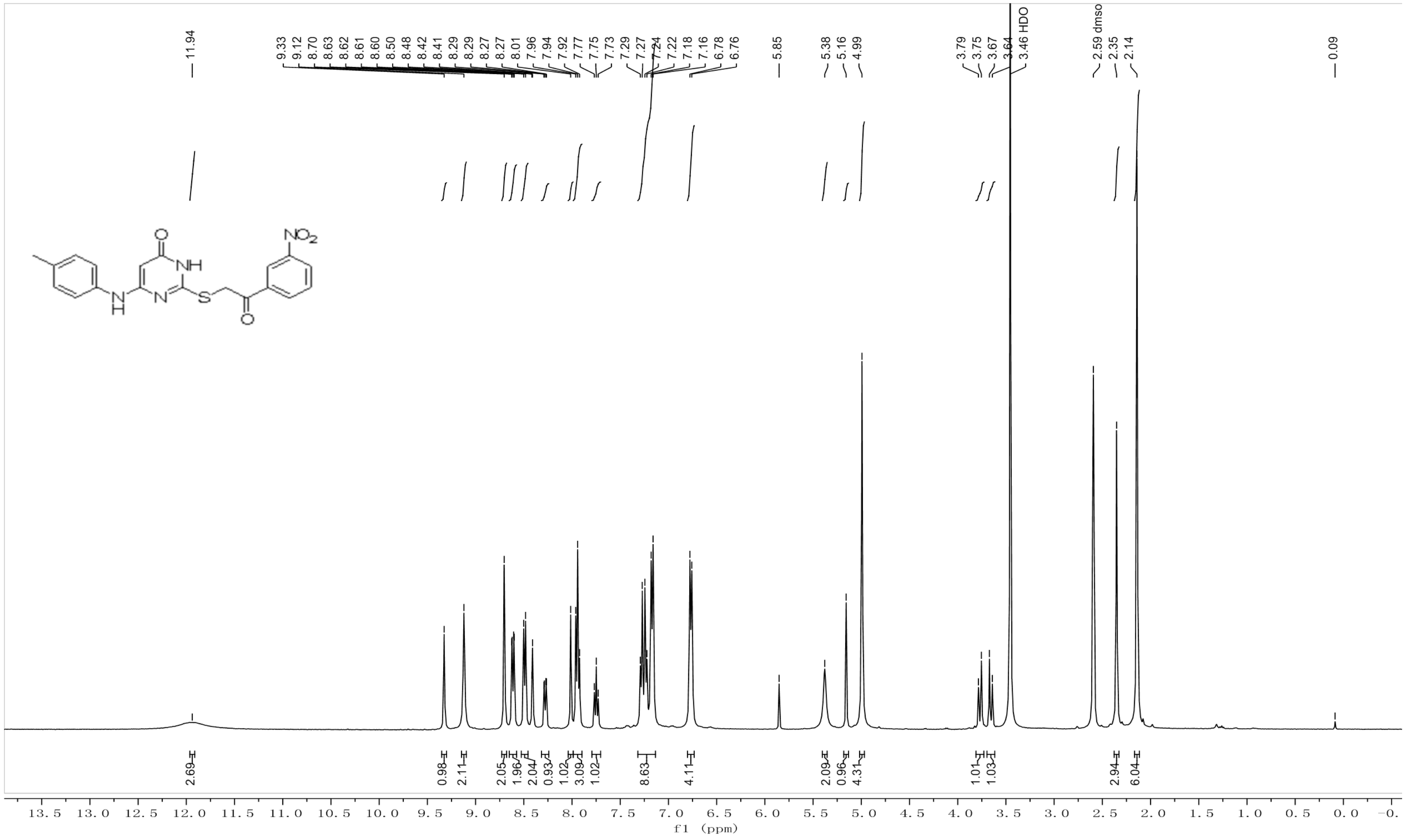

Figure S3. The NMR spectra of **2c**: 2-((2-(3-Nitrophenyl)-2-oxoethyl)thio)-6-(*p*-tolylamino)pyrimidin-4(3*H*)-one.

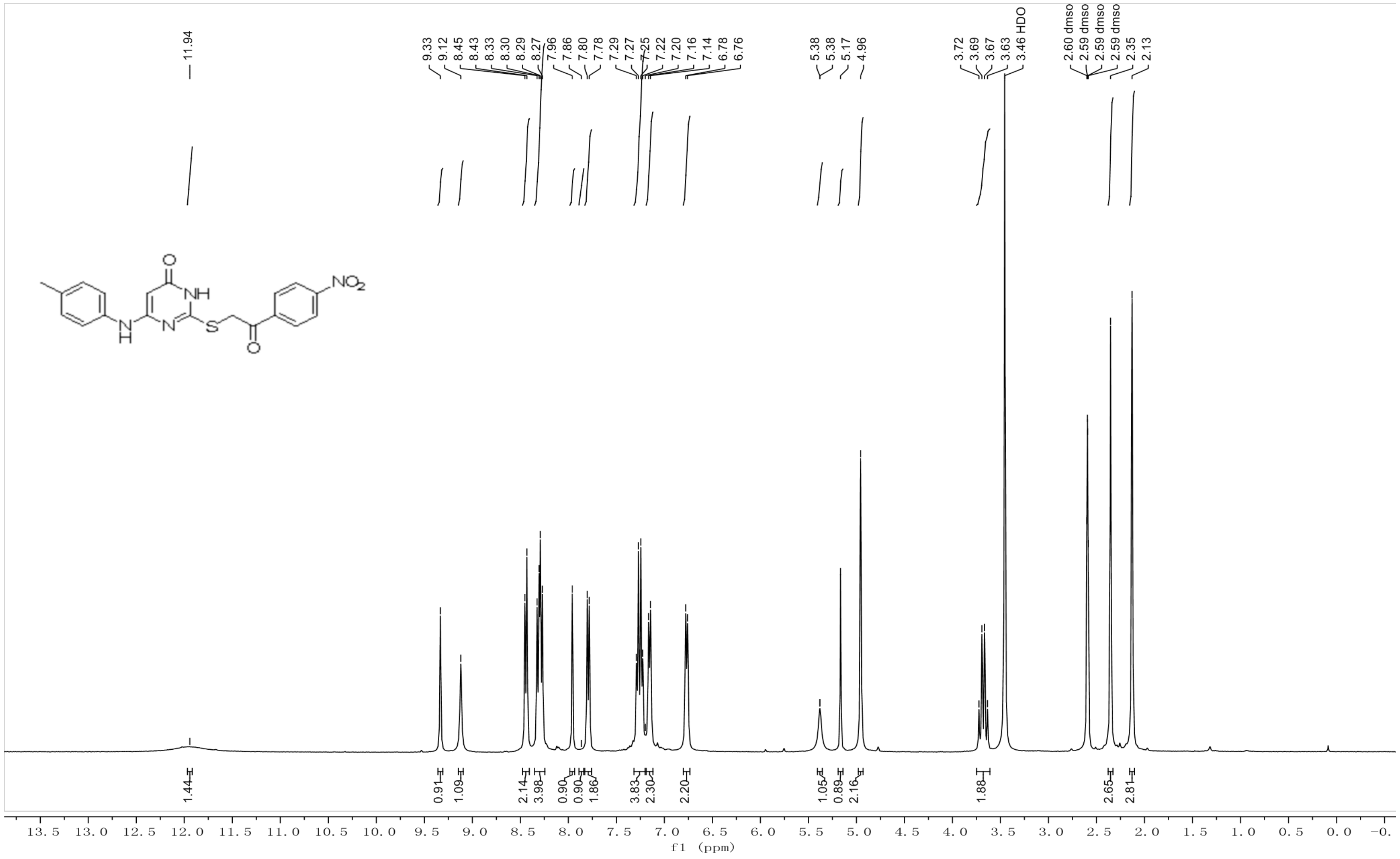

Figure S4. The NMR spectra of **2d**: 2-((2-(4-Nitrophenyl)-2-oxoethyl)thio)-6-(*p*-tolylamino)pyrimidin-4(3*H*)-one.

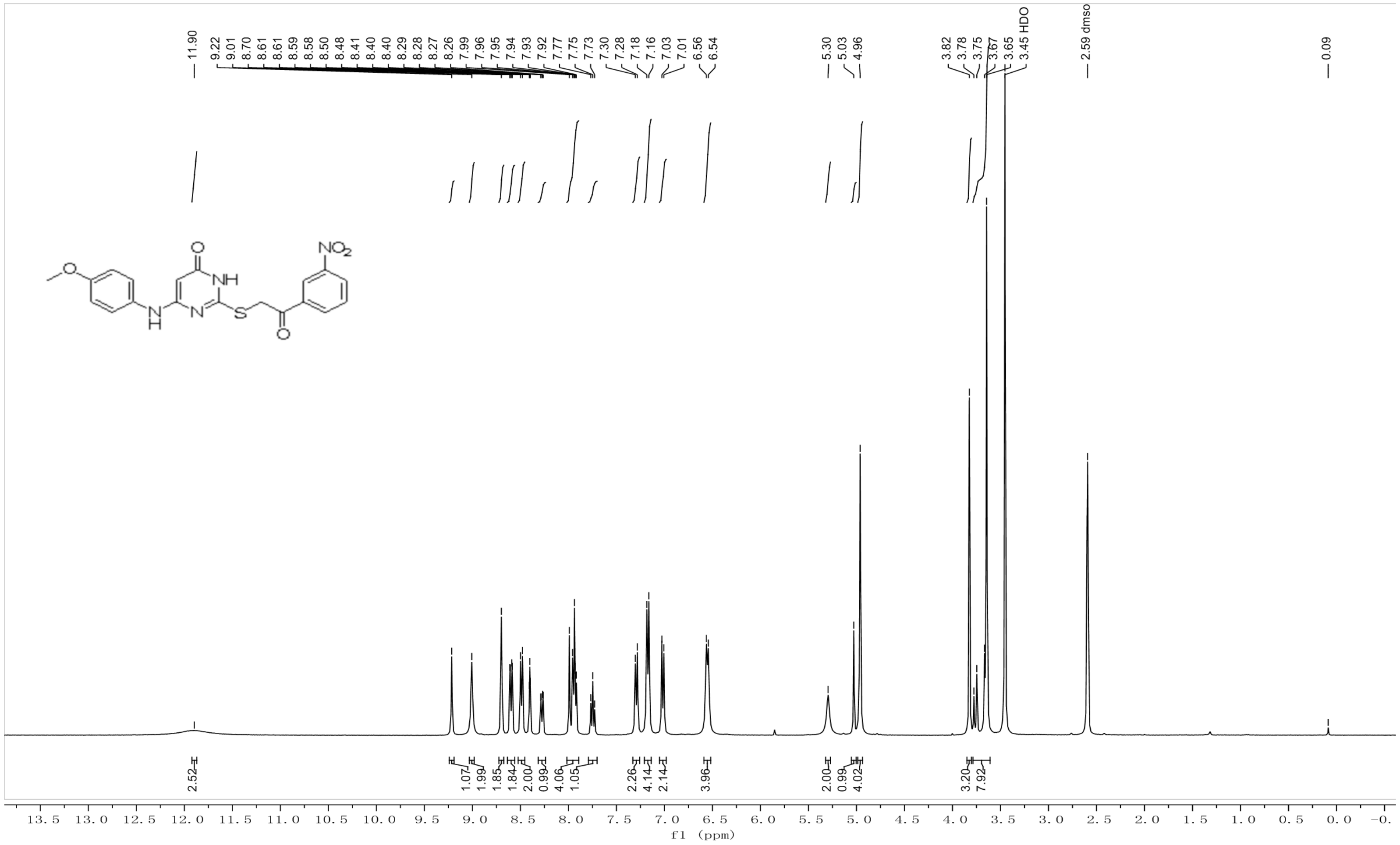

Figure S5. The NMR spectra of **2e**: 6-((4-Methoxyphenyl)amino)-2-((2-(3-nitrophenyl)-2-oxoethyl)thio)pyrimidin-4(3*H*)-one.

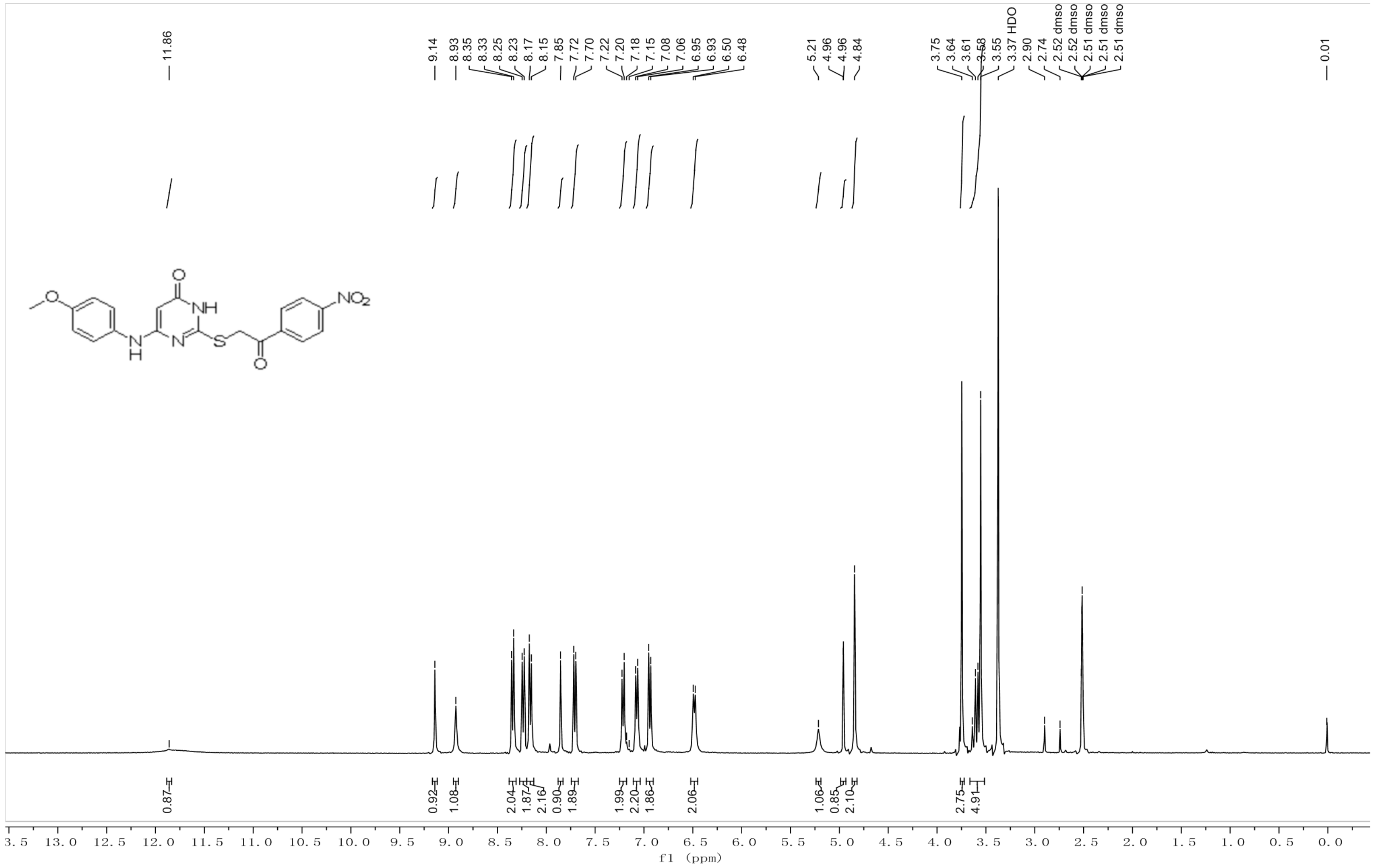

Figure S6. The NMR spectra of **2f**: 6-((4-Methoxyphenyl)amino)-2-((2-(4-nitrophenyl)-2-oxoethyl)thio)pyrimidin-4(3*H*)-one.

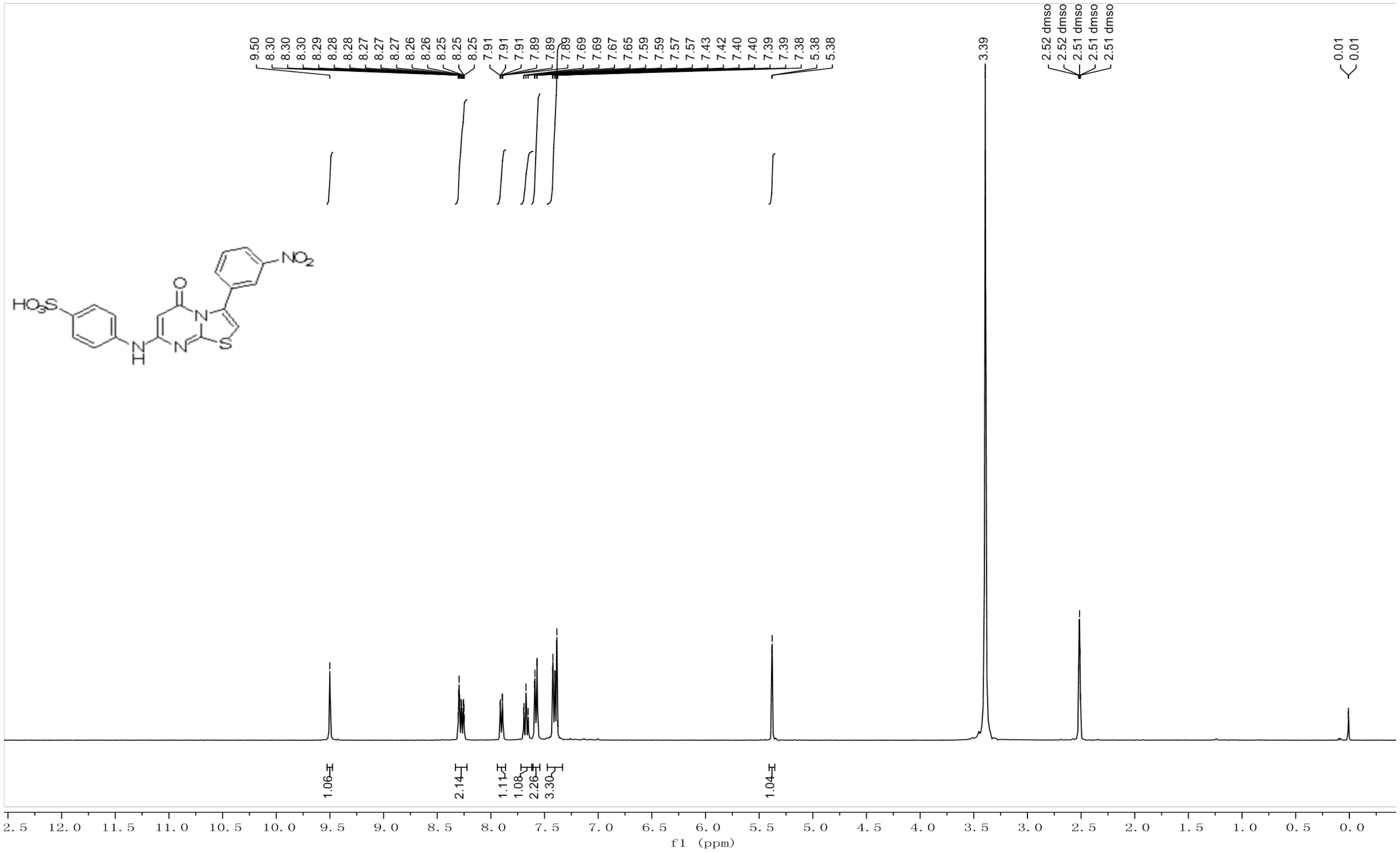

Figure S7. The ^{1}H-NMR spectra of **3a**: 4-((3-(3-Nitrophenyl)-5-oxo-5*H*-thiazolo[3,2-*a*]pyrimidin-7-yl)amino)benzenesulfonic acid.

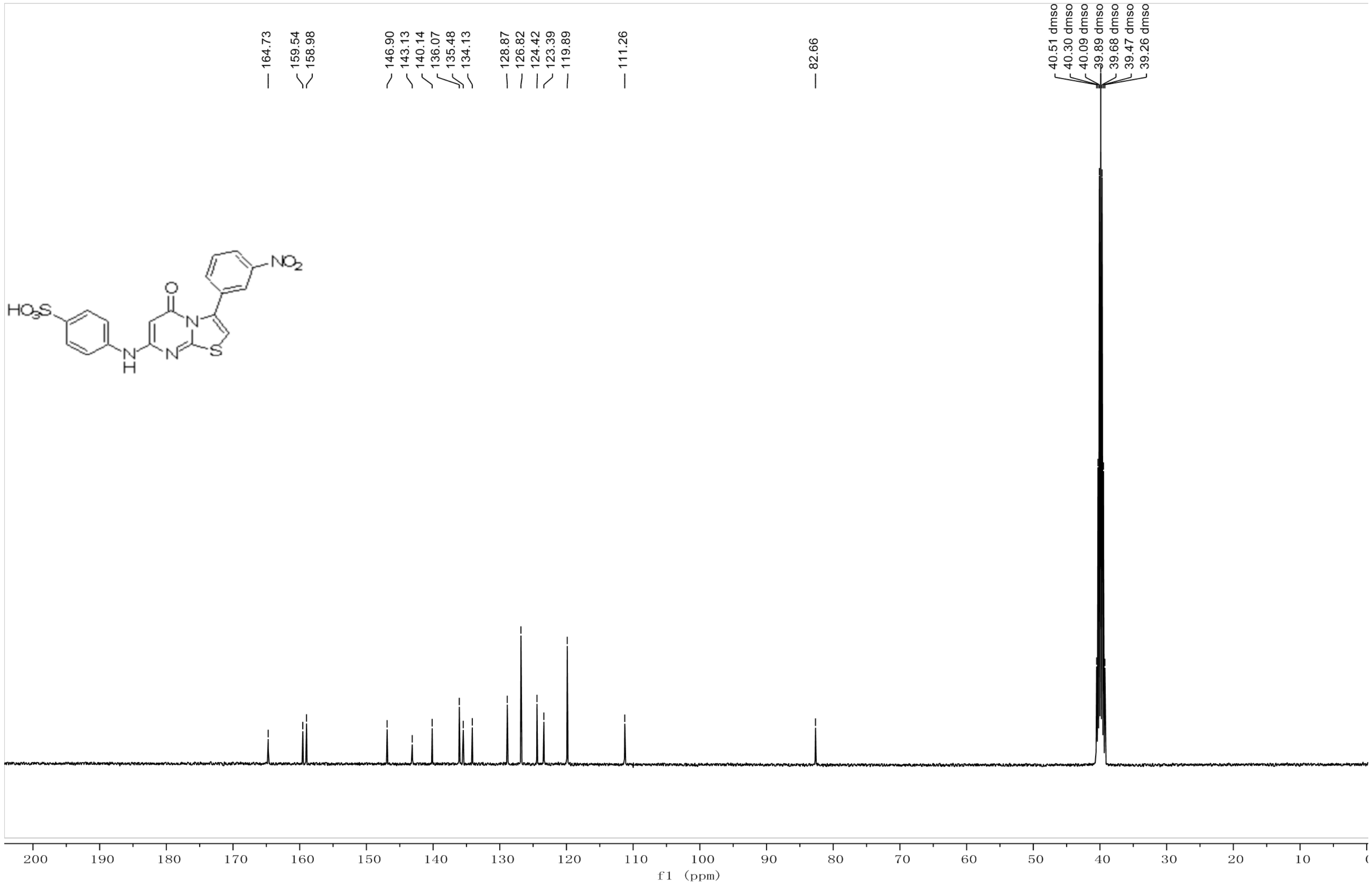

Figure S8. The ^{13}C-NMR spectra of **3a**: 4-((3-(3-Nitrophenyl)-5-oxo-5*H*-thiazolo[3,2-*a*]pyrimidin-7-yl)amino)benzenesulfonic acid.

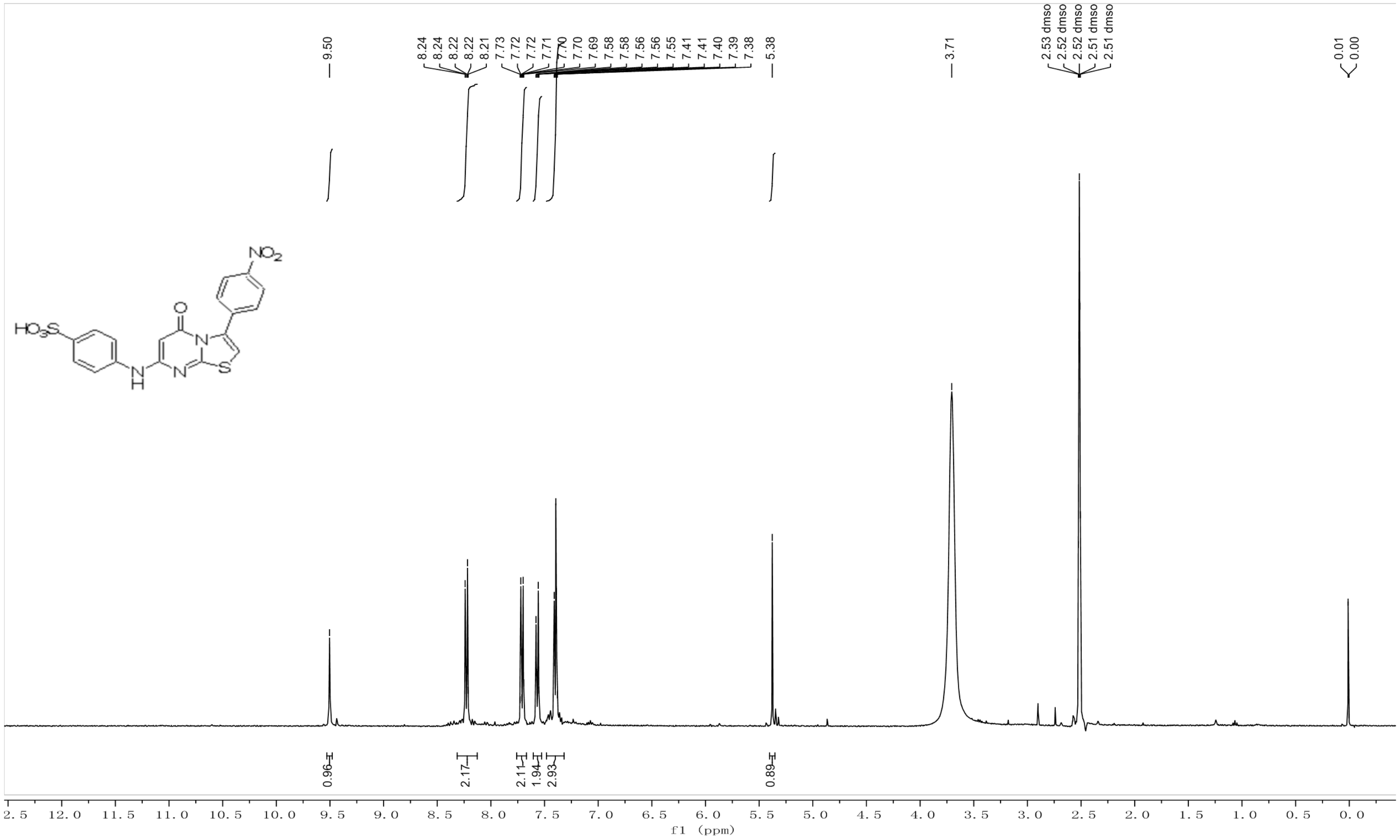

Figure S9. The ^{1}H-NMR spectra of **3b**: 4-((3-(4-Nitrophenyl)-5-oxo-5*H*-thiazolo[3,2-*a*]pyrimidin-7-yl)amino)benzenesulfonic acid.

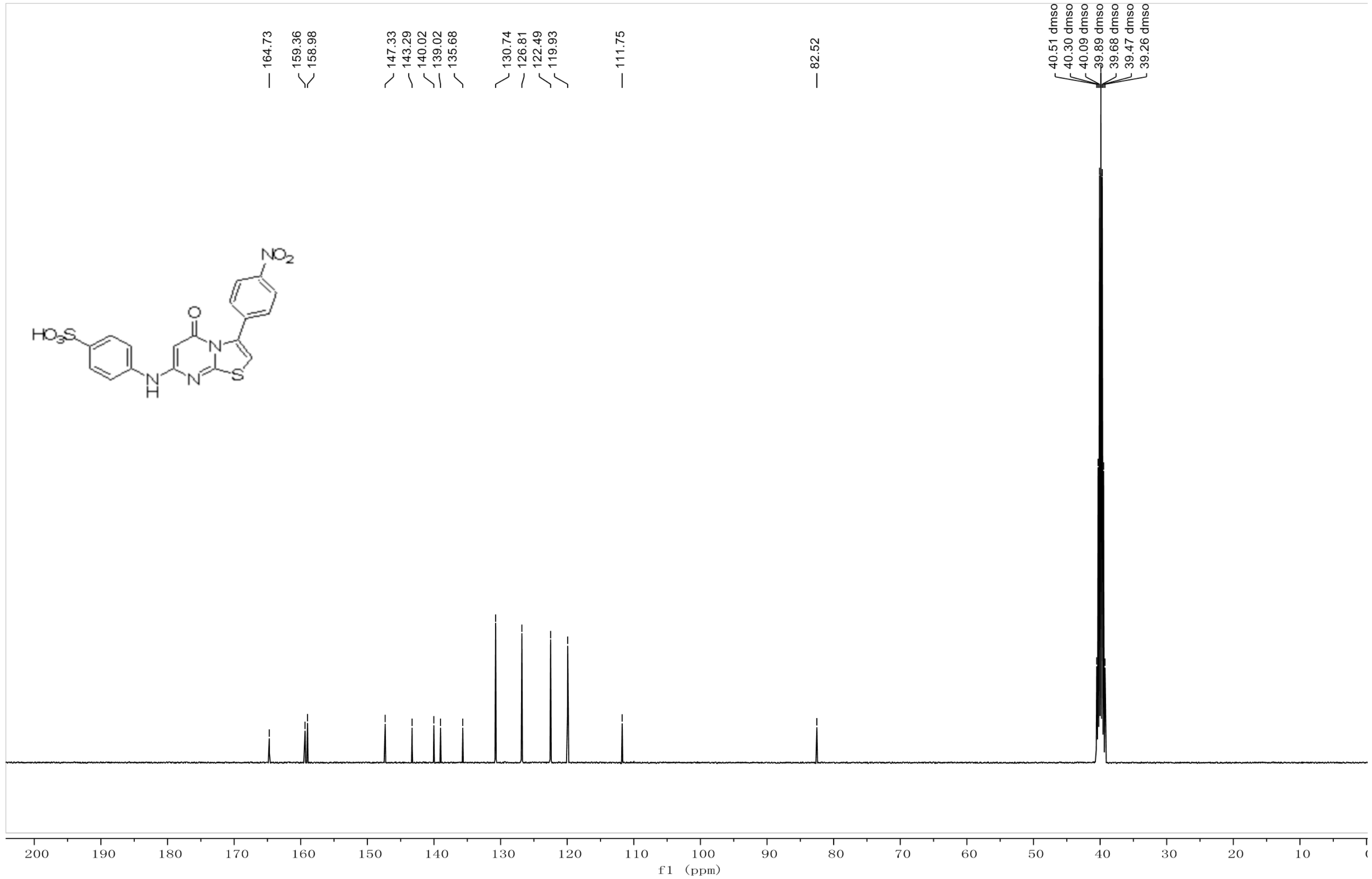

Figure S10. The ^{13}C-NMR spectra of **3b**: 4-((3-(4-Nitrophenyl)-5-oxo-5*H*-thiazolo[3,2-*a*]pyrimidin-7-yl)amino)benzenesulfonic acid.

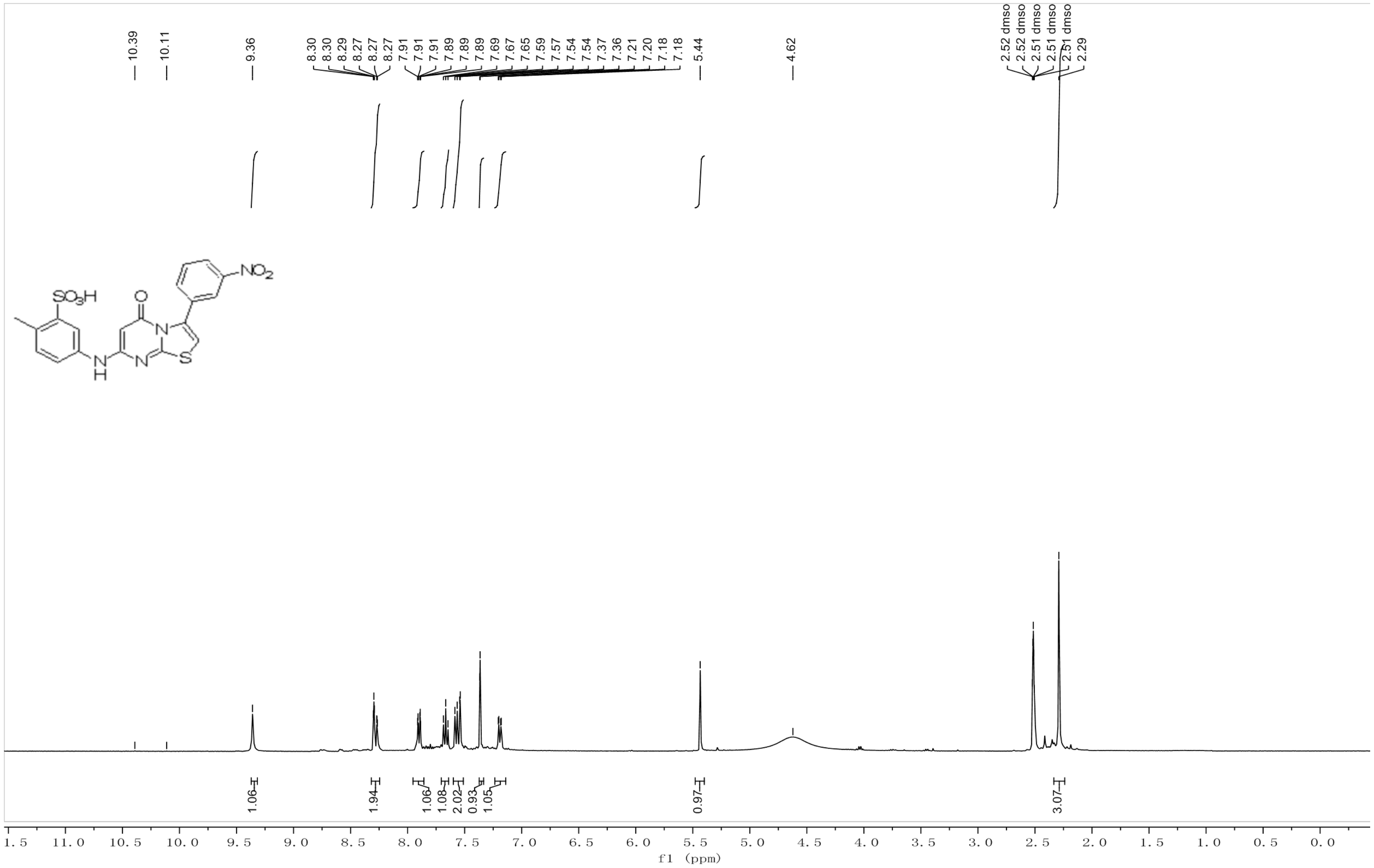

Figure S11. The ^{1}H-NMR spectra of **3c**: 2-Methyl-5-((3-(3-nitrophenyl)-5-oxo-5*H*-thiazolo[3,2-*a*]pyrimidin-7-yl)amino)benzenesulfonic acid.

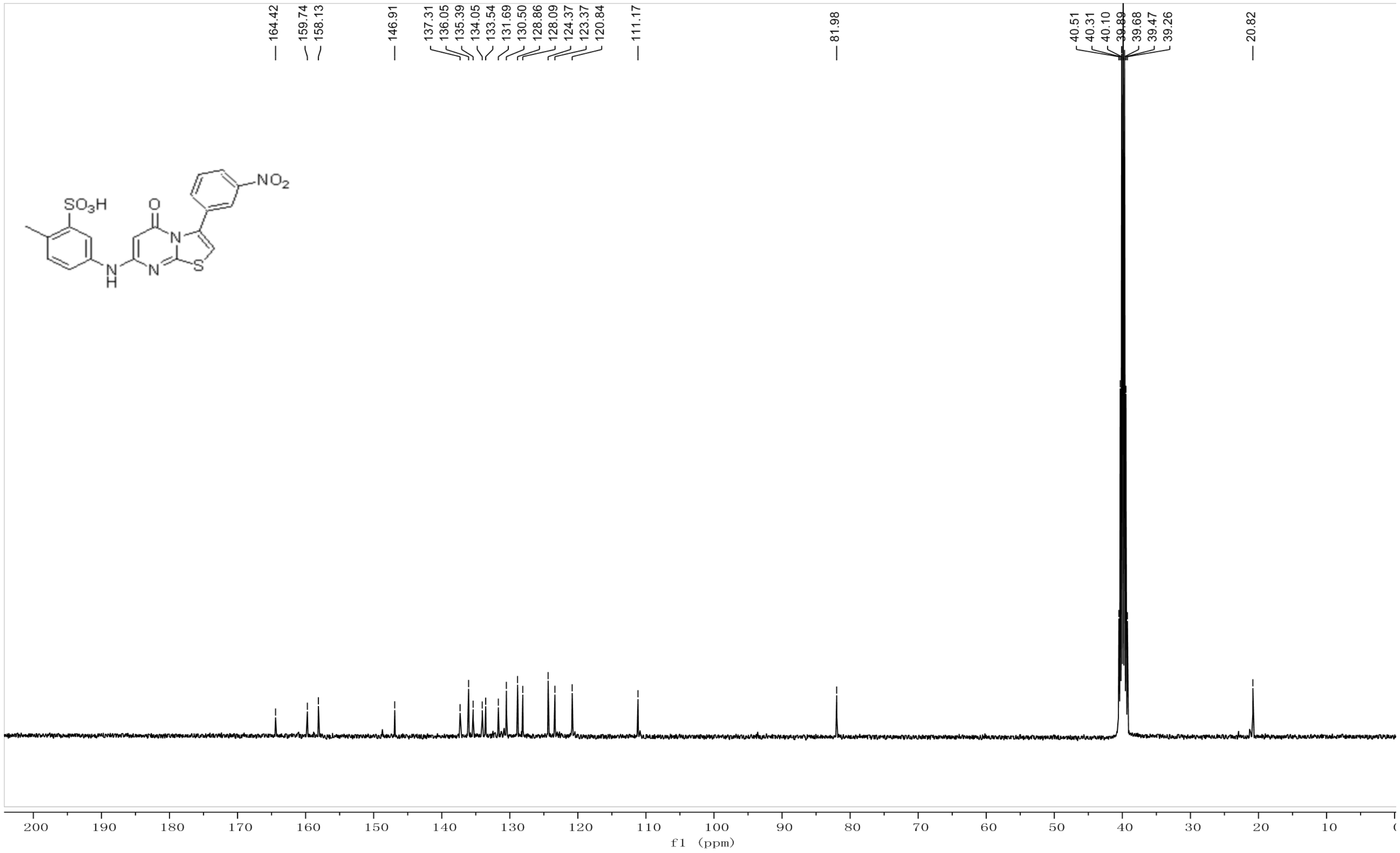

Figure S12. The ^{13}C-NMR spectra of **3c**: 2-Methyl-5-((3-(3-nitrophenyl)-5-oxo-5*H*-thiazolo[3,2-*a*]pyrimidin-7-yl)amino)benzenesulfonic acid.

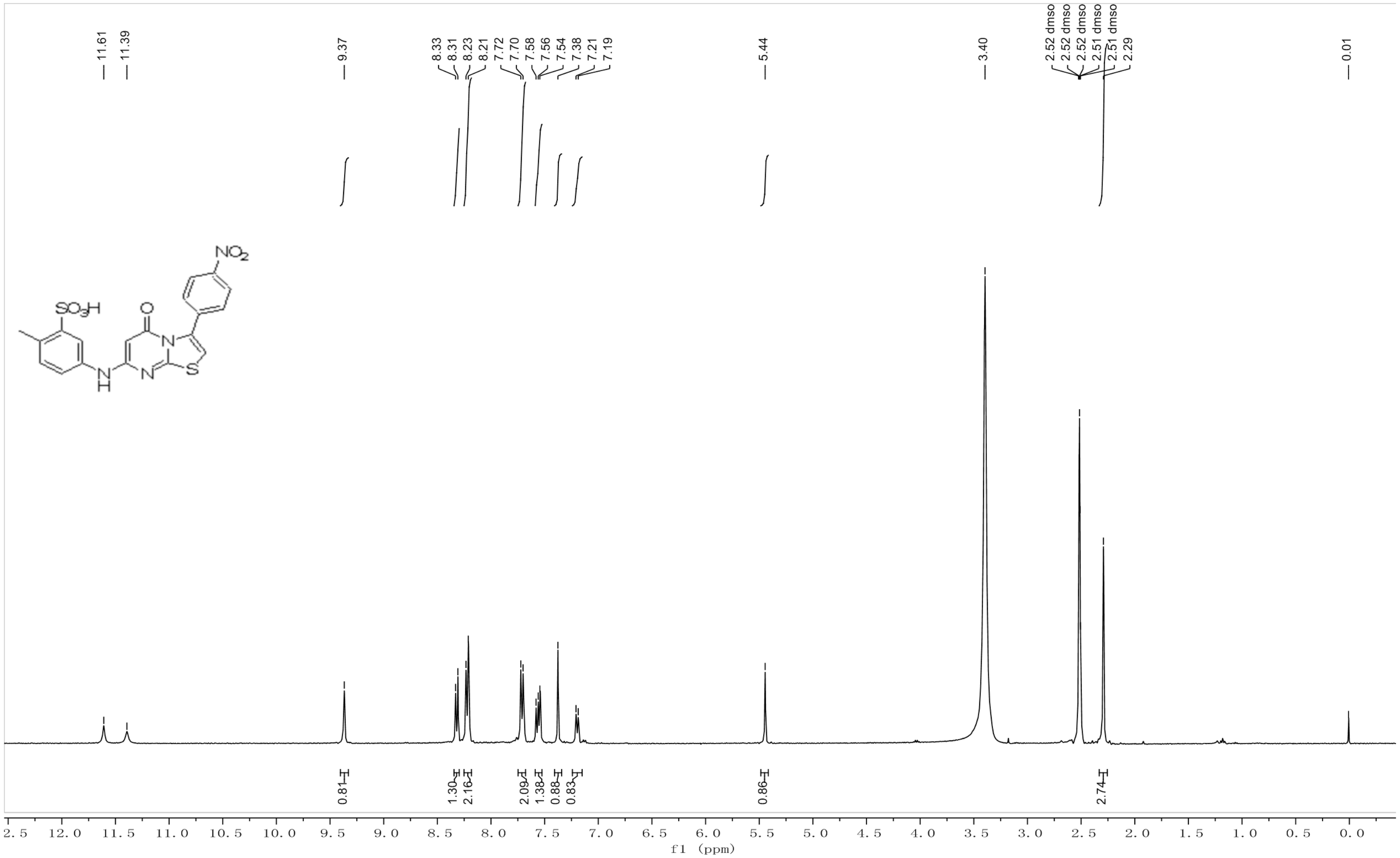

Figure S13. The ^{1}H-NMR spectra of **3d**: 2-Methyl-5-((3-(4-nitrophenyl)-5-oxo-5*H*-thiazolo[3,2-*a*]pyrimidin-7-yl)amino)benzenesulfonic acid.

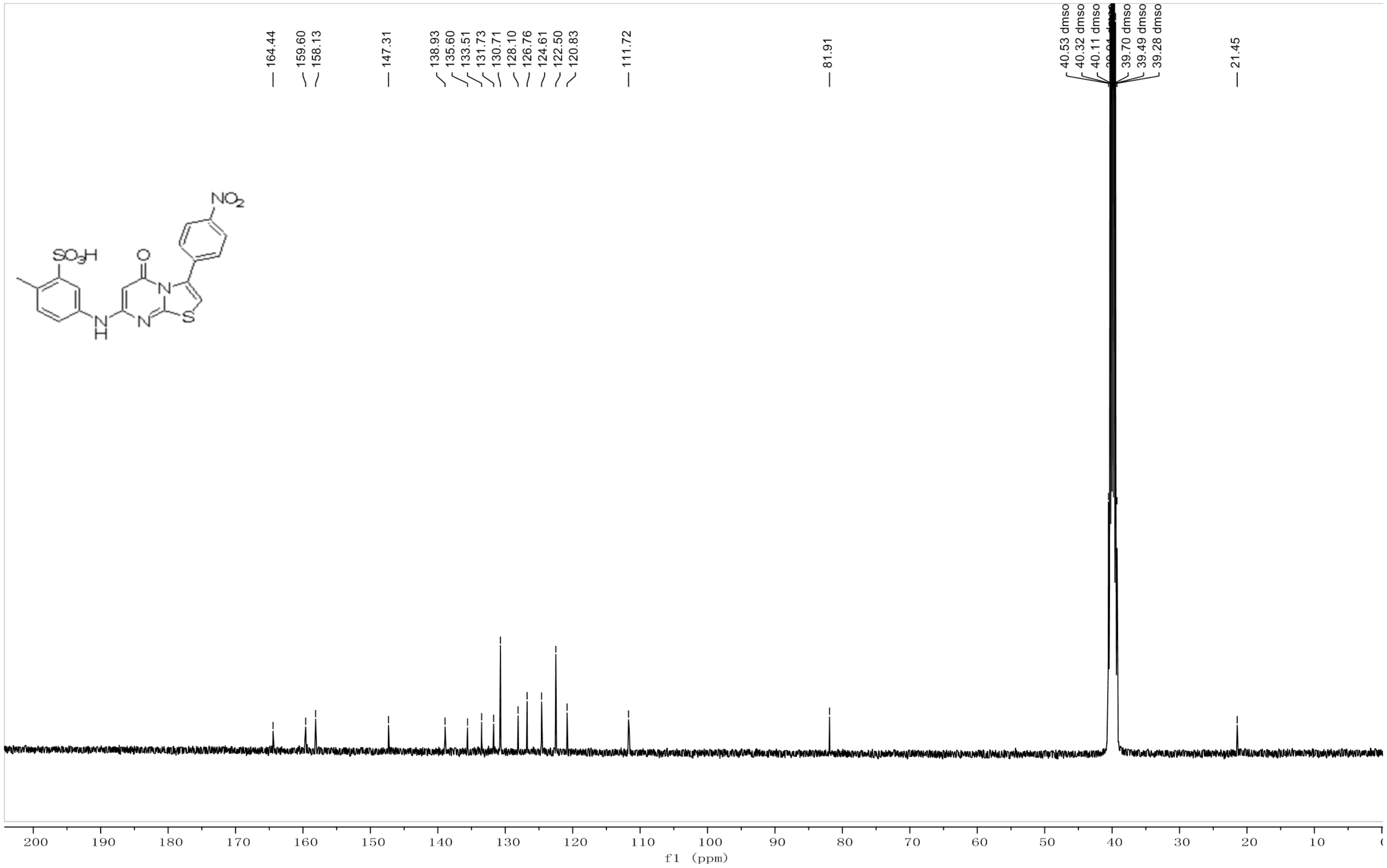

Figure S14. The ^{13}C-NMR spectra of **3d**: 2-Methyl-5-((3-(4-nitrophenyl)-5-oxo-5*H*-thiazolo[3,2-*a*]pyrimidin-7-yl)amino)benzenesulfonic acid.

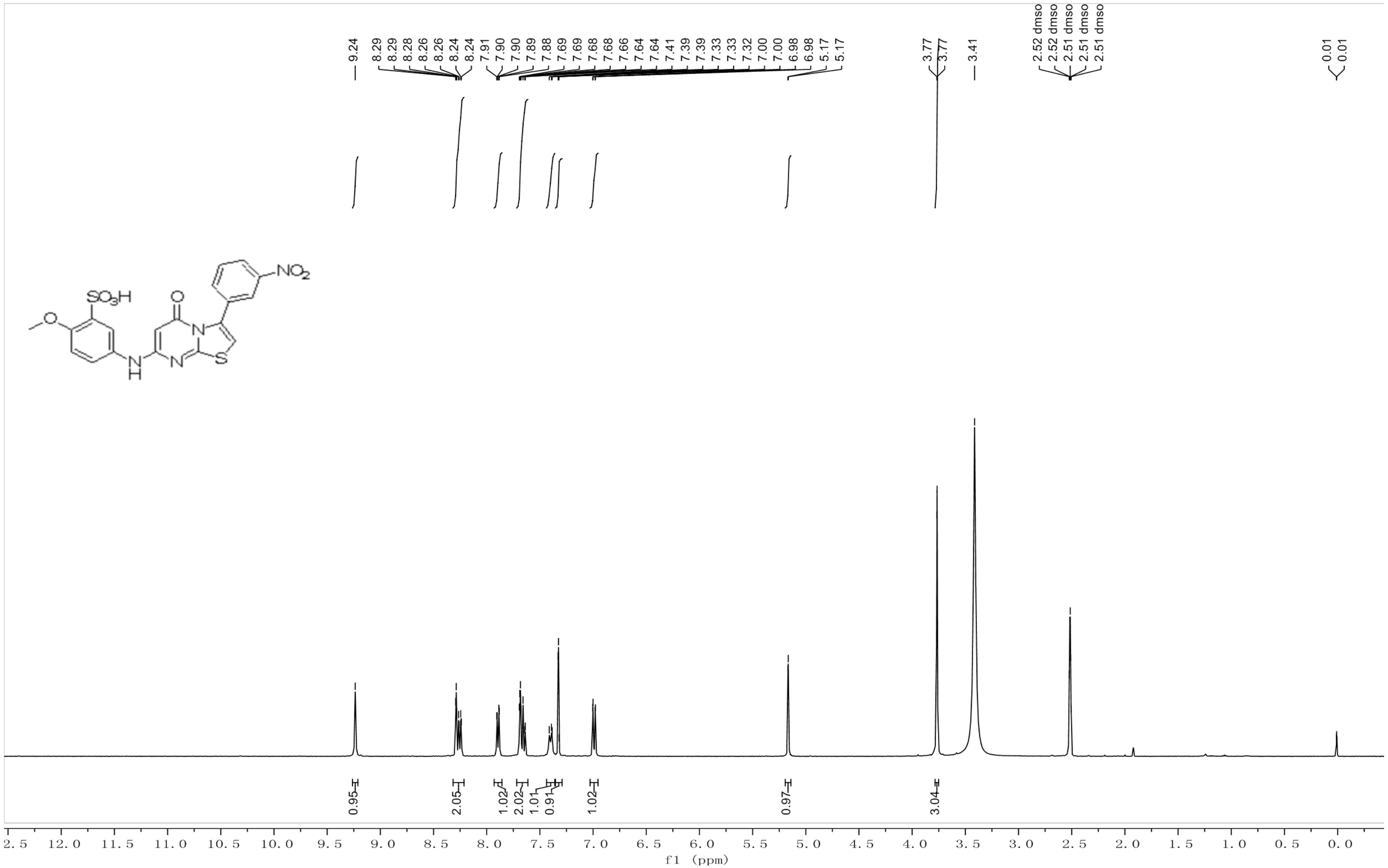

Figure S15. The ^{1}H-NMR spectra of **3e**: 2-Methoxy-5-((3-(3-nitrophenyl)-5-oxo-5*H*-thiazolo[3,2-*a*]pyrimidin-7-yl)amino)benzenesulfonic acid.

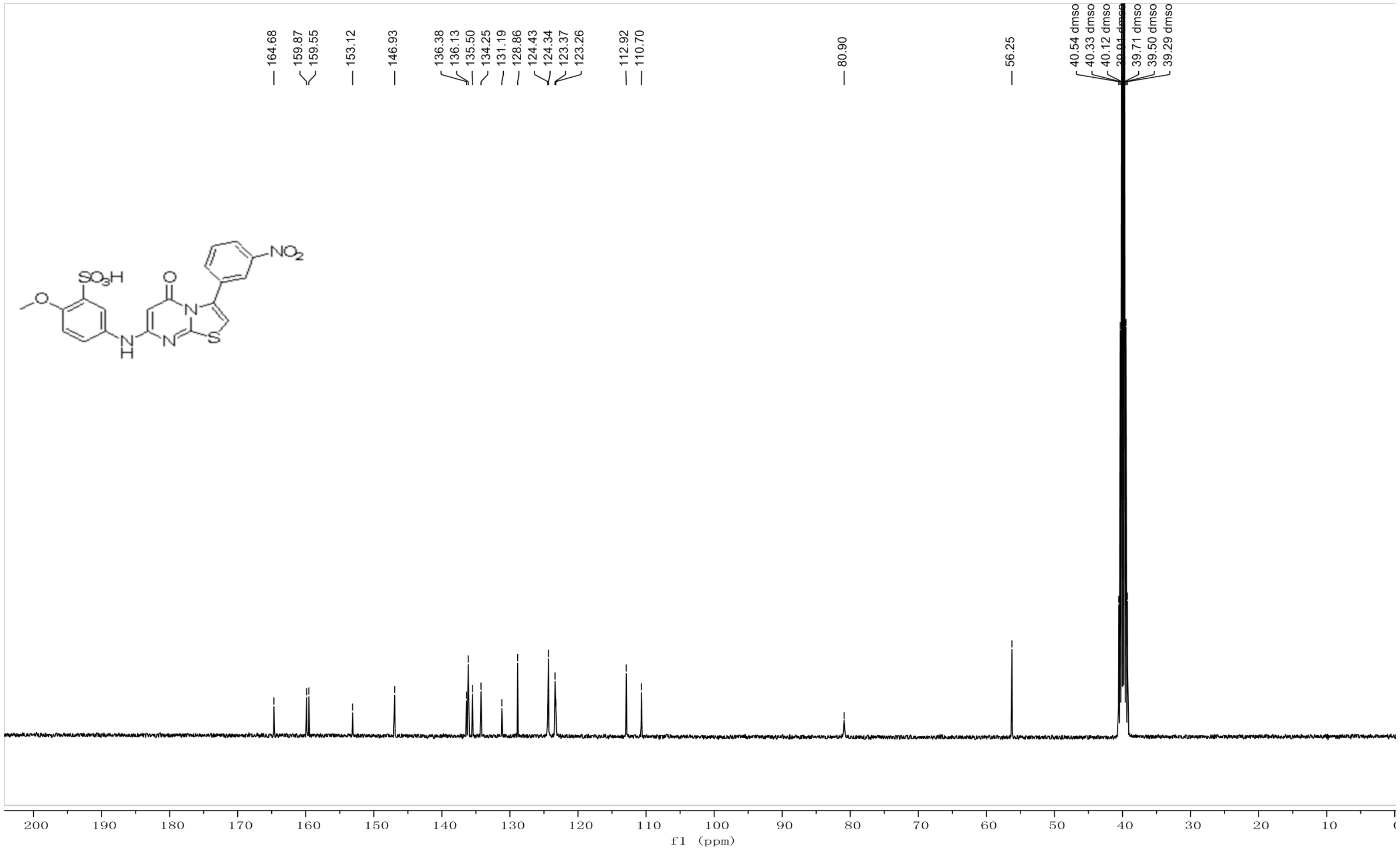

Figure S16. The ^{13}C-NMR spectra of **3e**: 2-Methoxy-5-((3-(3-nitrophenyl)-5-oxo-5*H*-thiazolo[3,2-*a*]pyrimidin-7-yl)amino)benzenesulfonic acid.

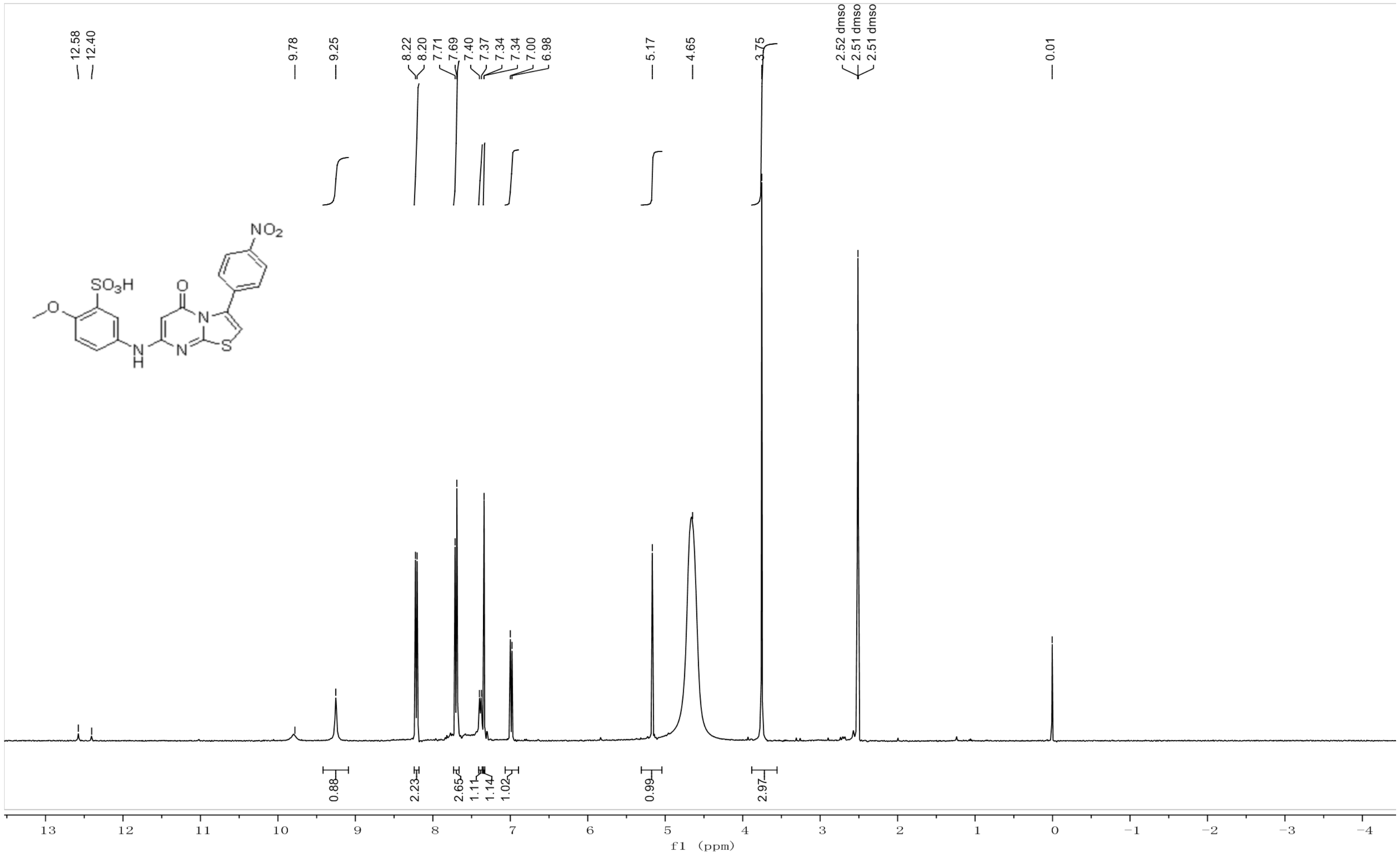

Figure S17. The ^{1}H-NMR spectra of **3f**: 2-Methoxy-5-((3-(4-nitrophenyl)-5-oxo-5*H*-thiazolo[3,2-*a*]pyrimidin-7-yl)amino)benzenesulfonic acid.

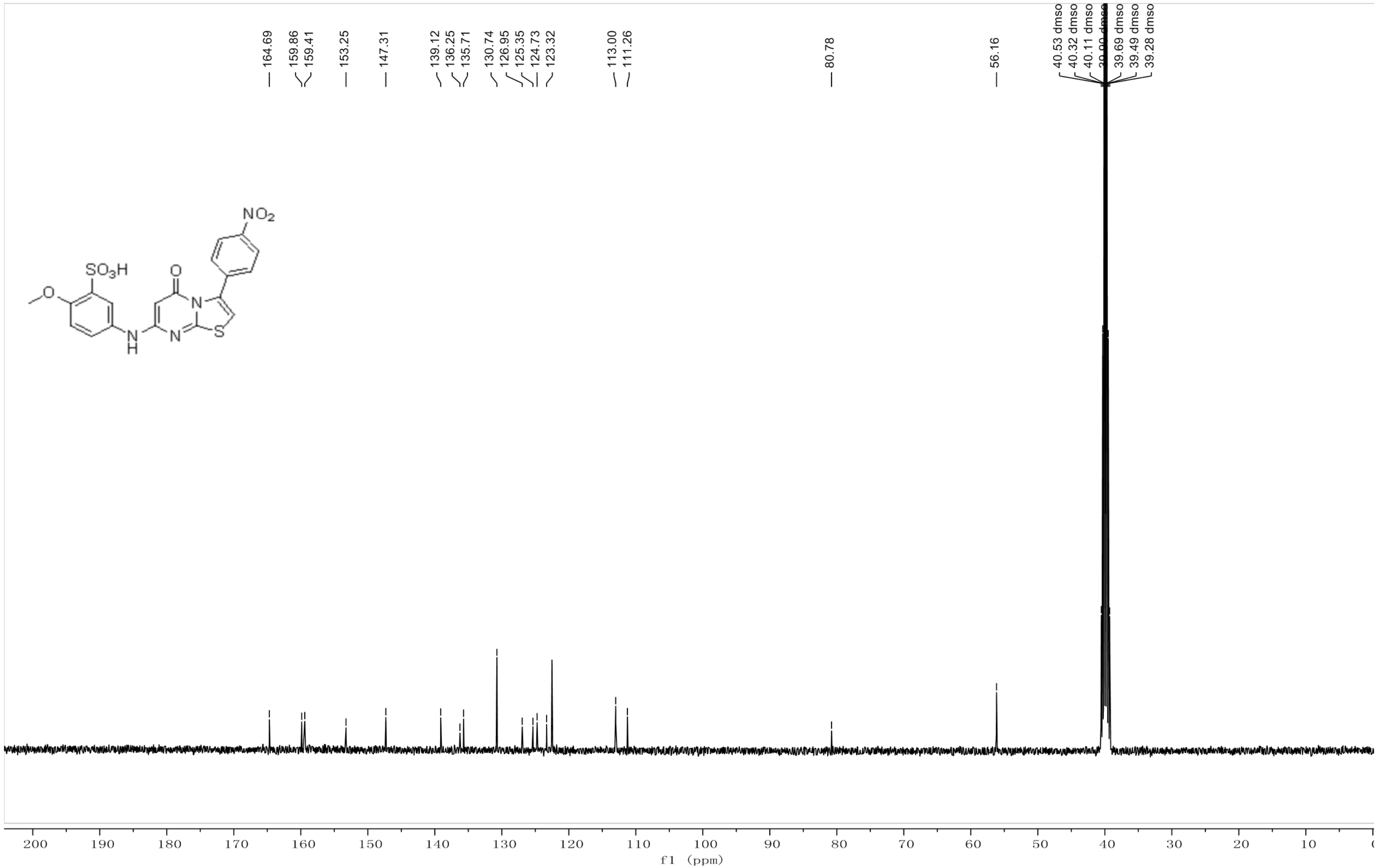

Figure S18. The ^{13}C-NMR spectra of **3f**: 2-Methoxy-5-((3-(4-nitrophenyl)-5-oxo-5*H*-thiazolo[3,2-*a*]pyrimidin-7-yl)amino)benzenesulfonic acid.

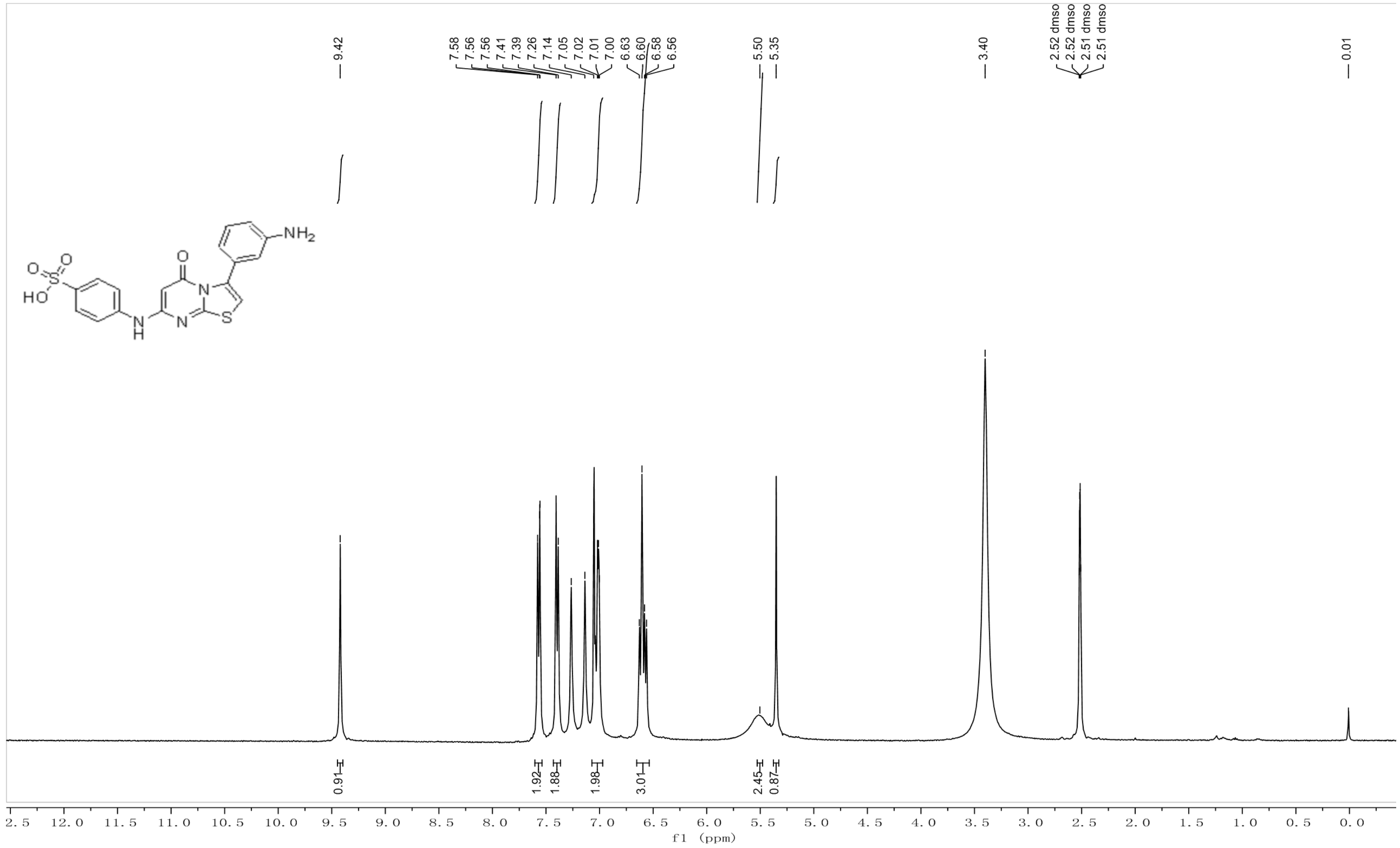

Figure S19. The ^{1}H-NMR spectra of **4a**: 4-((3-(3-Aminophenyl)-5-oxo-5*H*-thiazolo[3,2-*a*]pyrimidin-7-yl)amino)benzenesulfonic acid.

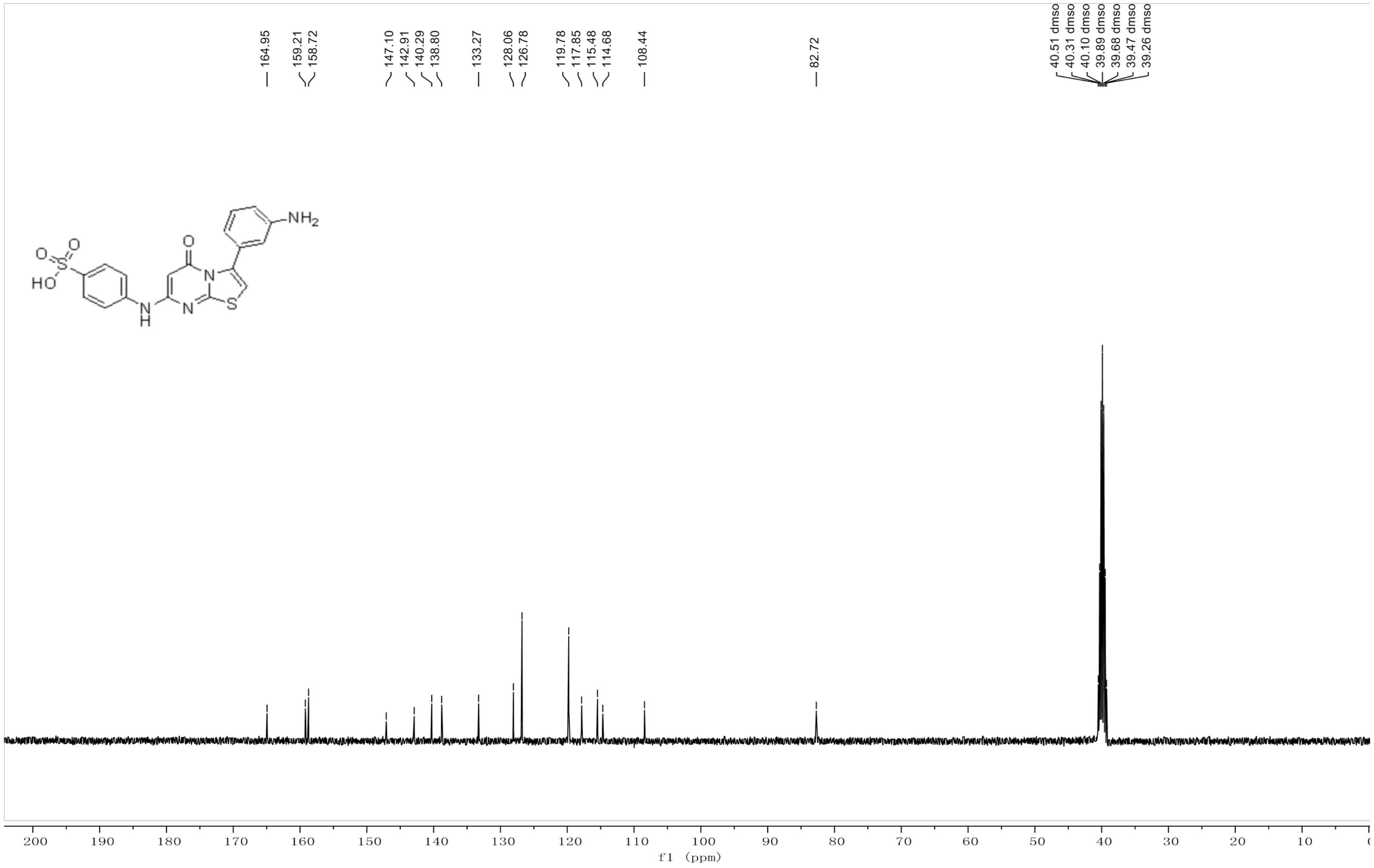

Figure S20. The ^{13}C-NMR spectra of **4a**: 4-((3-(3-Aminophenyl)-5-oxo-5*H*-thiazolo[3,2-*a*]pyrimidin-7-yl)amino)benzenesulfonic acid.

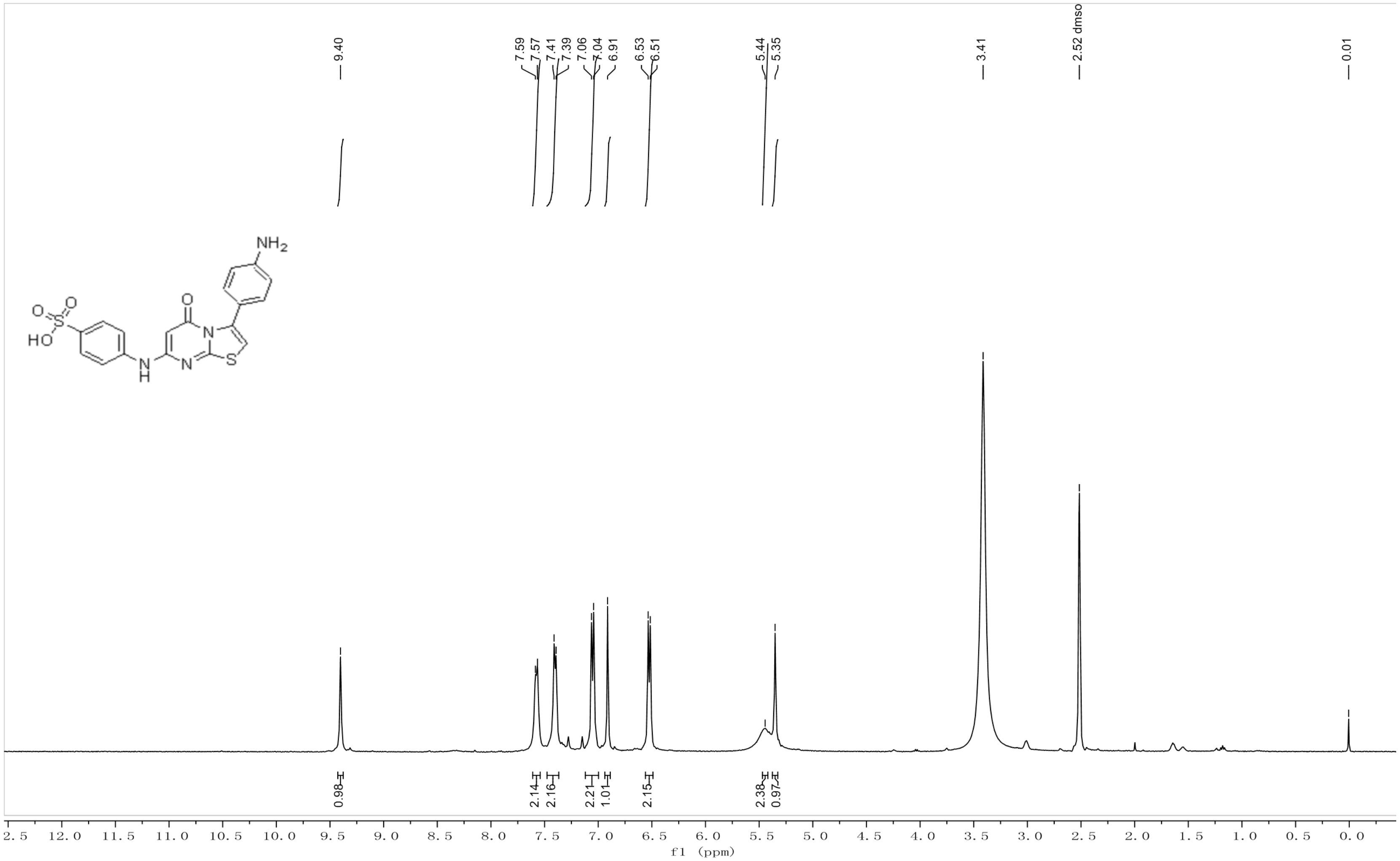

Figure S21. The ^{1}H-NMR spectra of **4b**: 4-((3-(4-Aminophenyl)-5-oxo-5*H*-thiazolo[3,2-*a*]pyrimidin-7-yl)amino)benzenesulfonic acid.

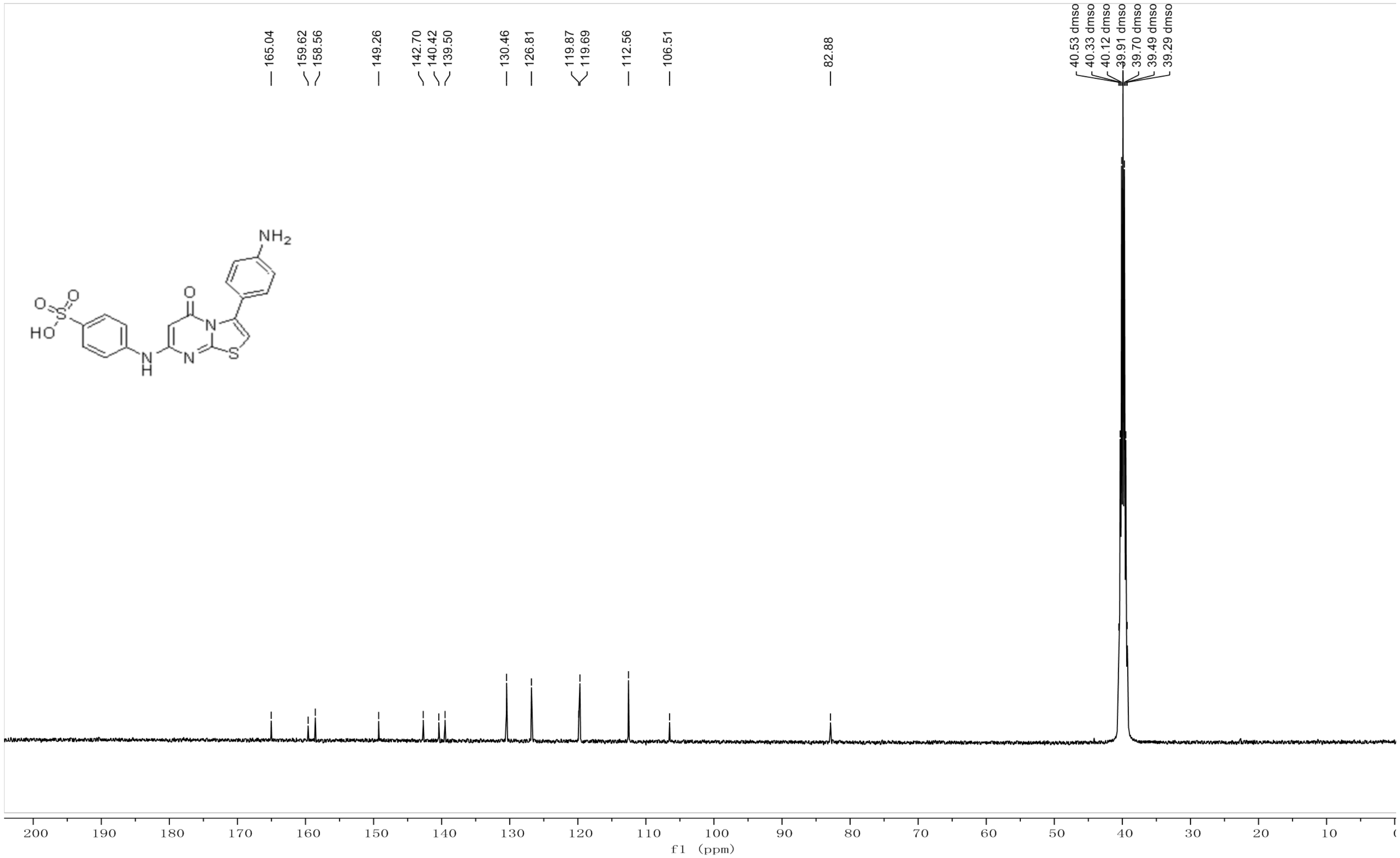

Figure S22. The ^{13}C-NMR spectra of **4b**: 4-((3-(4-Aminophenyl)-5-oxo-5*H*-thiazolo[3,2-*a*]pyrimidin-7-yl)amino)benzenesulfonic acid.

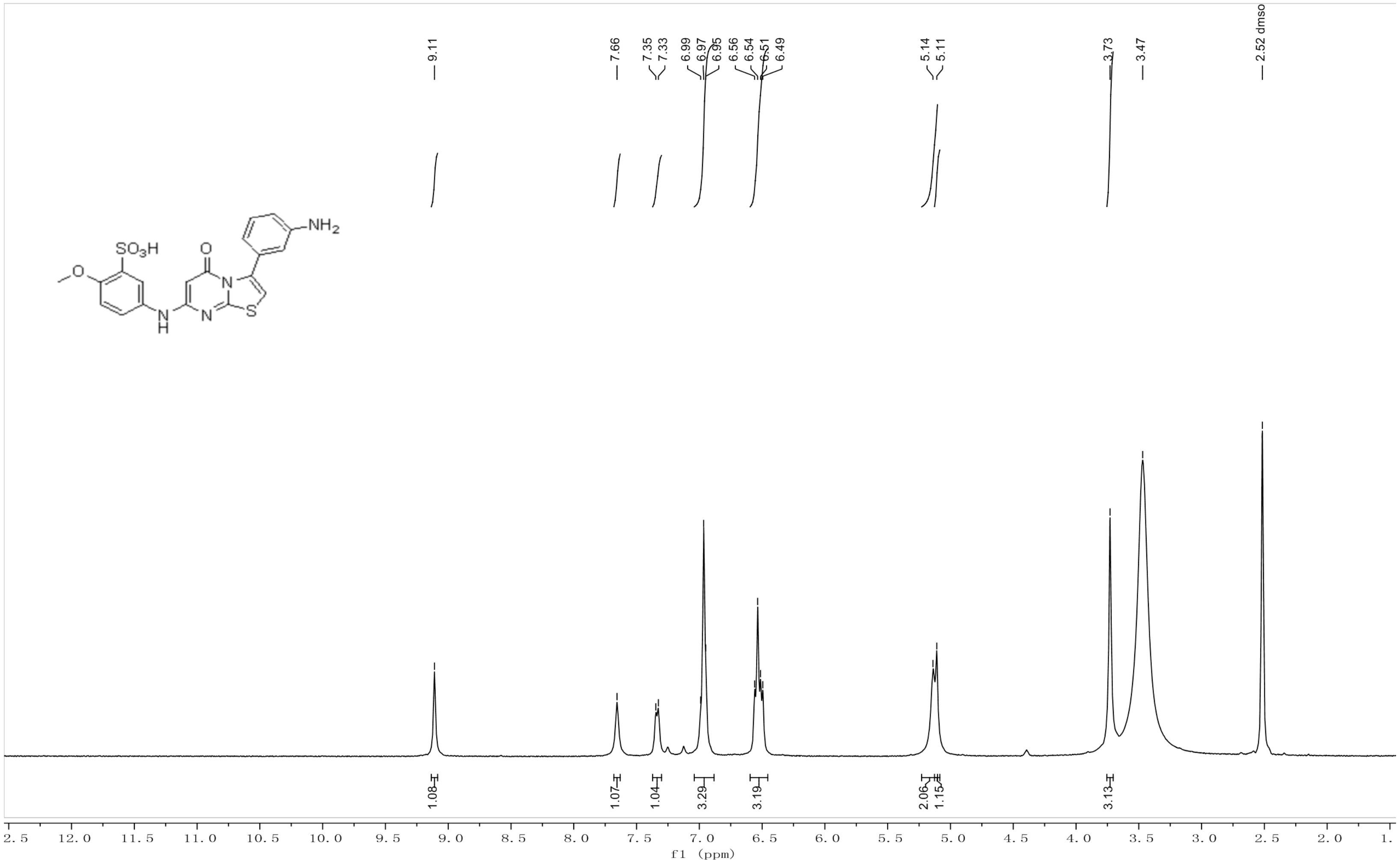

Figure S23. The ^{1}H-NMR spectra of **4c**: 5-((3-(3-Aminophenyl)-5-oxo-5*H*-thiazolo[3,2-*a*]pyrimidin-7-yl)amino)-2-methoxybenzenesulfonic acid.

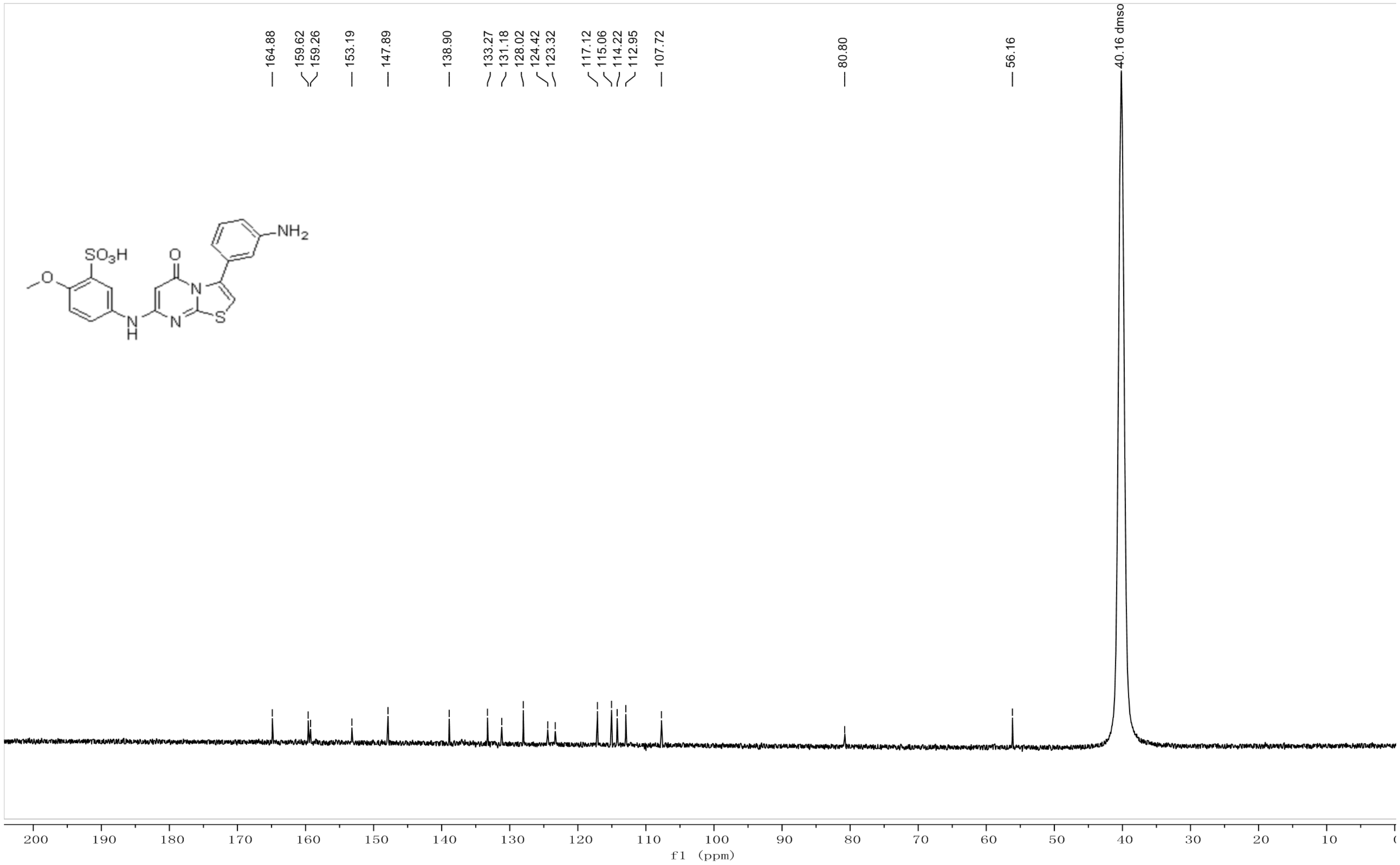

Figure S24. The ^{13}C-NMR spectra of **4c**: 5-((3-(3-Aminophenyl)-5-oxo-5*H*-thiazolo[3,2-*a*]pyrimidin-7-yl)amino)-2-methoxybenzenesulfonic acid.

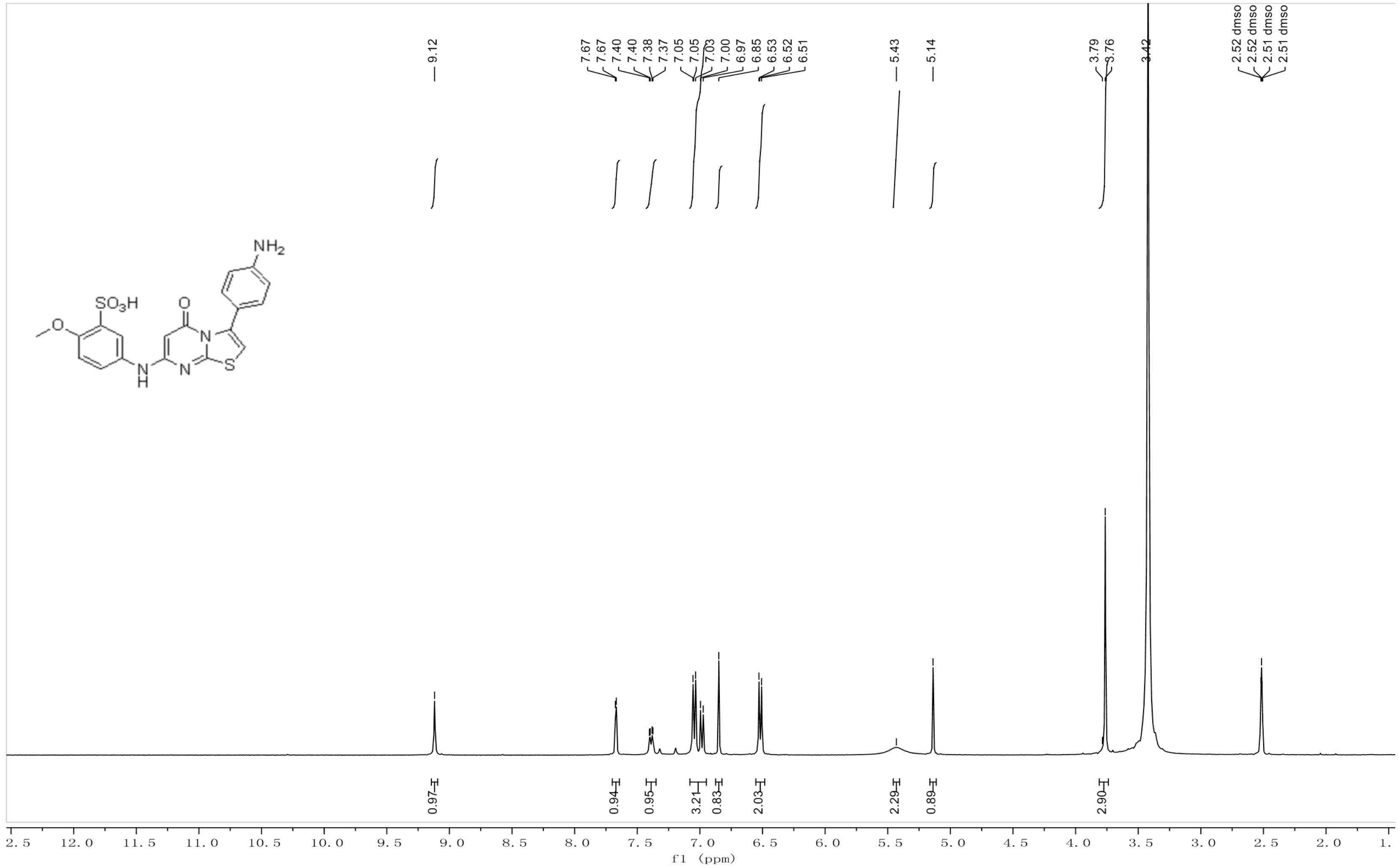

Figure S25. The ^{1}H-NMR spectra of **4d**: 5-((3-(4-Aminophenyl)-5-oxo-5*H*-thiazolo[3,2-*a*]pyrimidin-7-yl)amino)-2-methoxybenzenesulfonic acid.

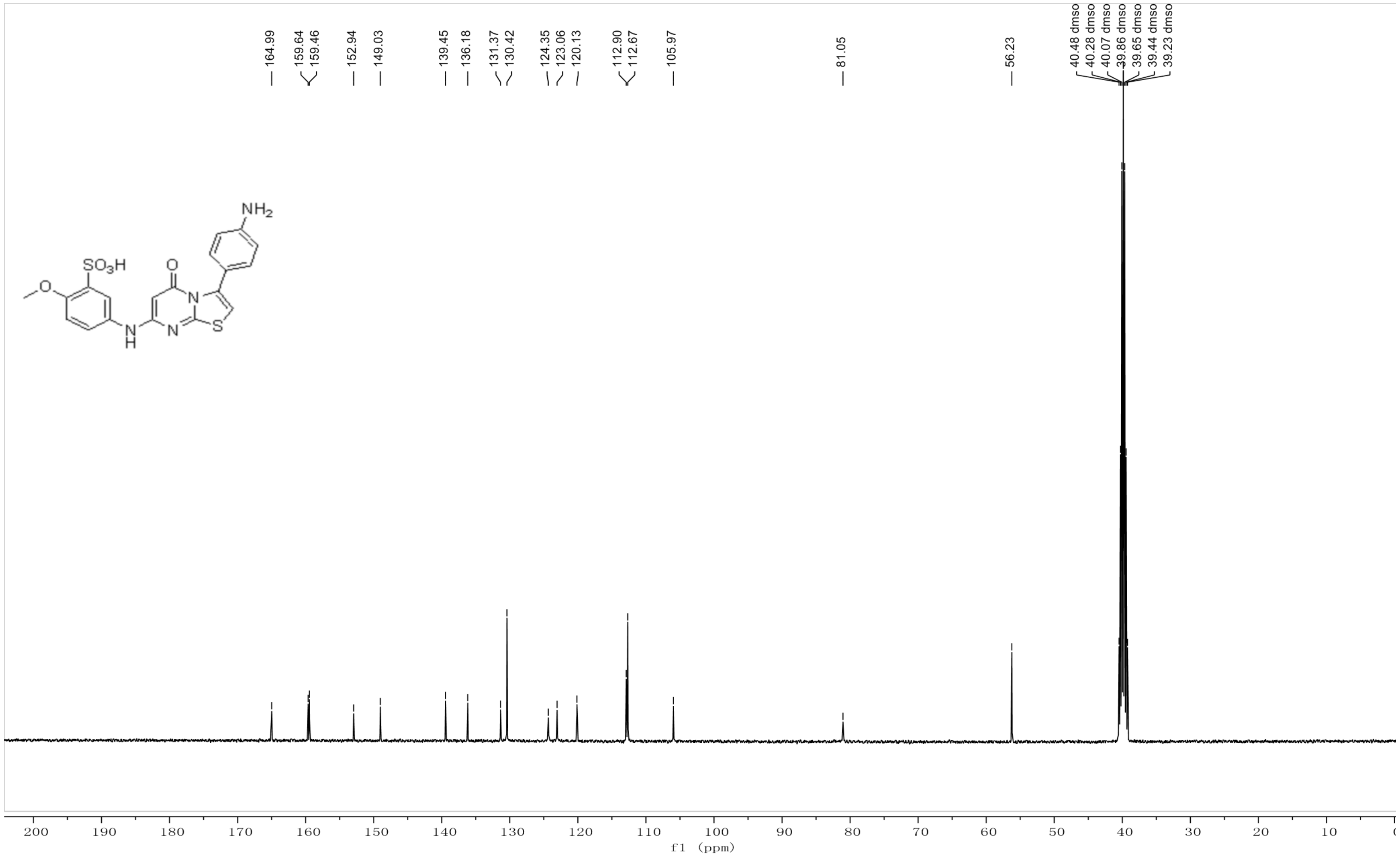

Figure S26. The ^{13}C-NMR spectra of **4d**: 5-((3-(4-Aminophenyl)-5-oxo-5*H*-thiazolo[3,2-*a*]pyrimidin-7-yl)amino)-2-methoxybenzenesulfonic acid.

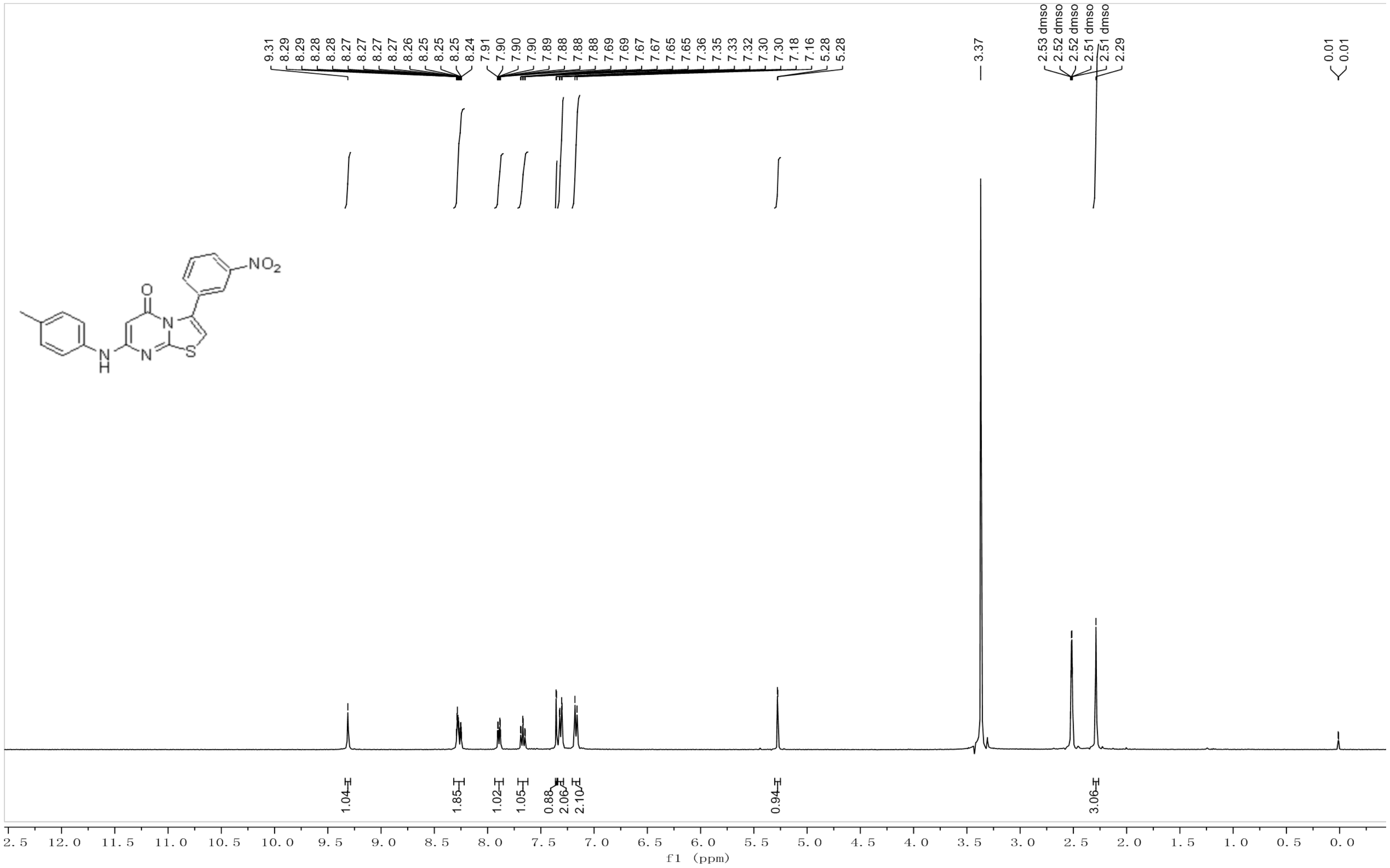

Figure S27. The ^{1}H-NMR spectra of **5a**: 3-(4-Nitrophenyl)-7-(*p*-tolylamino)-5*H*-thiazolo[3,2-*a*]pyrimidin-5-one.

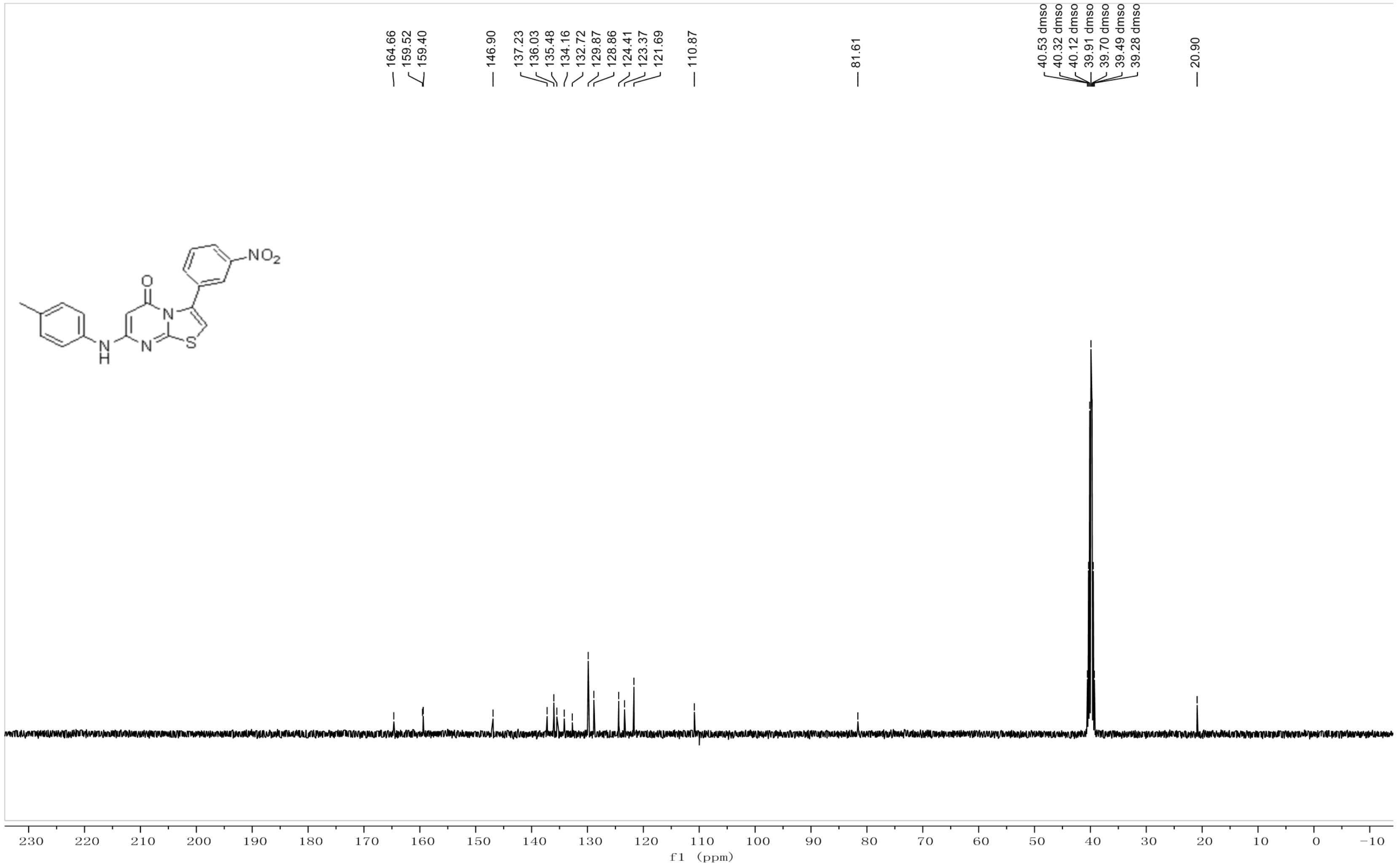

Figure S28. The ^{13}C-NMR spectra of **5a**: 3-(4-Nitrophenyl)-7-(*p*-tolylamino)-5*H*-thiazolo[3,2-*a*]pyrimidin-5-one.

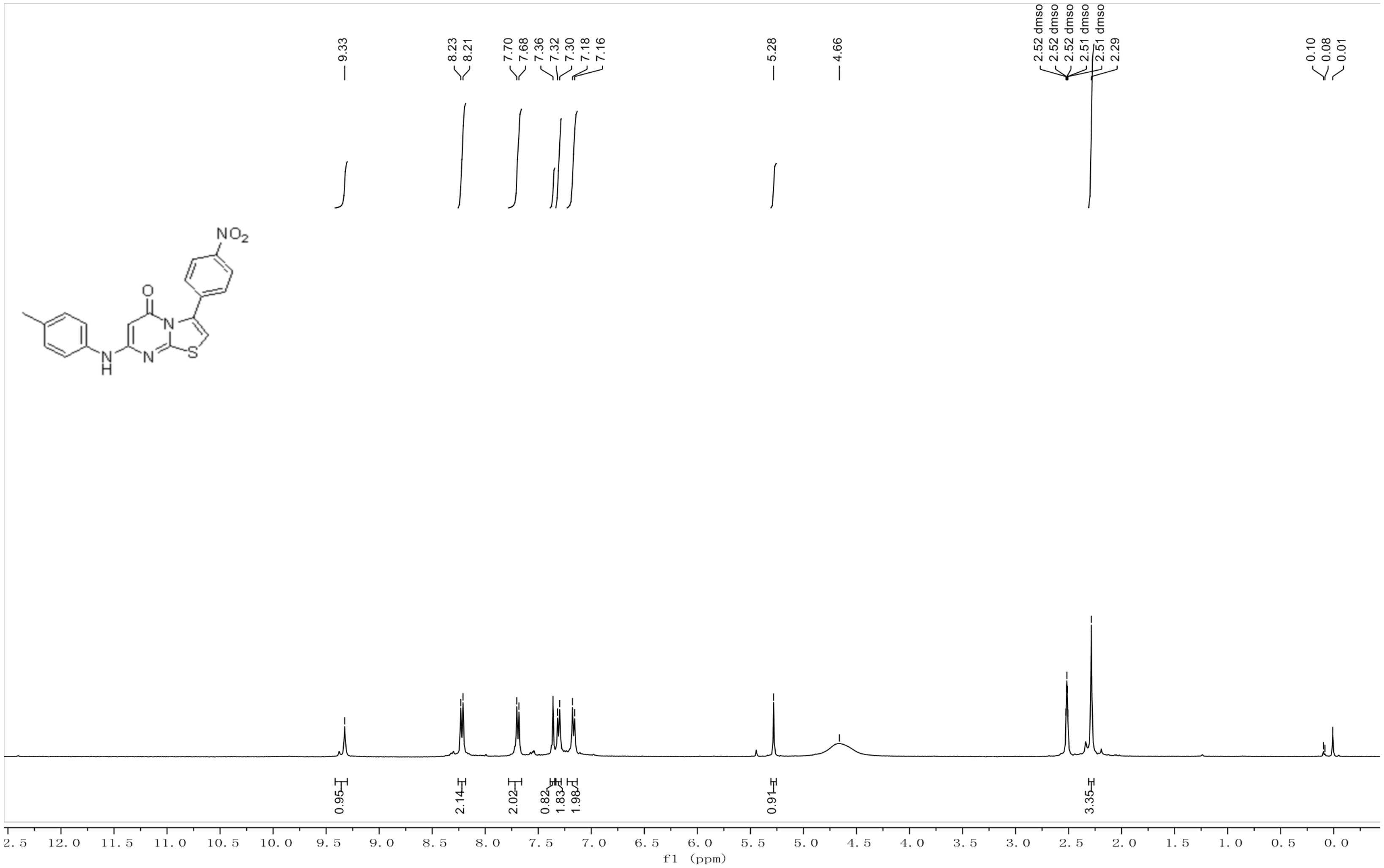

Figure S29. The ^{1}H-NMR spectra of **5b**: 3-(4-Nitrophenyl)-7-(*p*-tolylamino)-5*H*-thiazolo[3,2-*a*]pyrimidin-5-one.

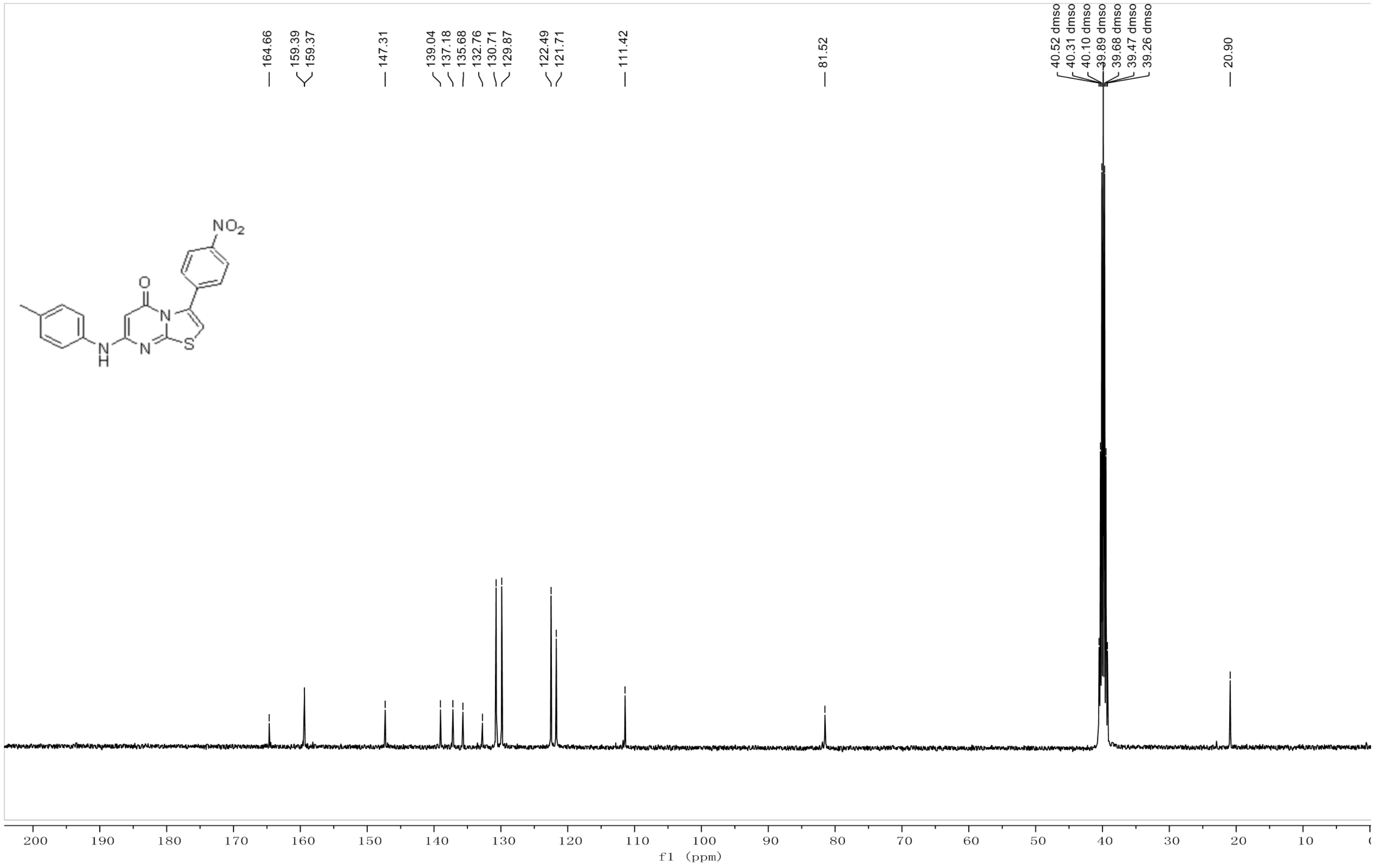

Figure S30. The ^{13}C-NMR spectra of **5b**: 3-(4-Nitrophenyl)-7-(*p*-tolylamino)-5*H*-thiazolo[3,2-*a*]pyrimidin-5-one.

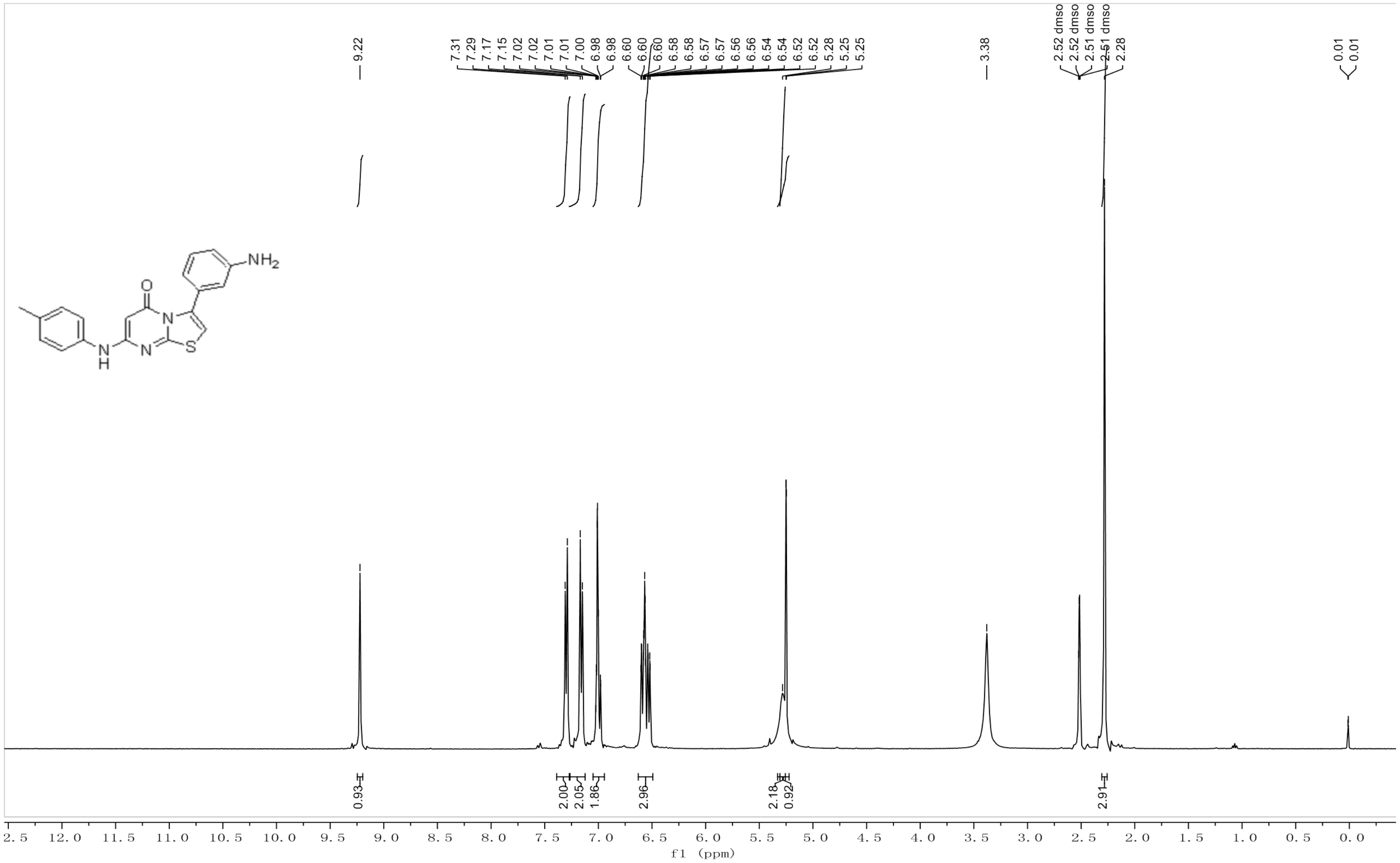

Figure S31. The ^{1}H-NMR spectra of **6a**: 3-(3-Aminophenyl)-7-(*p*-tolylamino)-5*H*-thiazolo[3,2-*a*]pyrimidin-5-one.

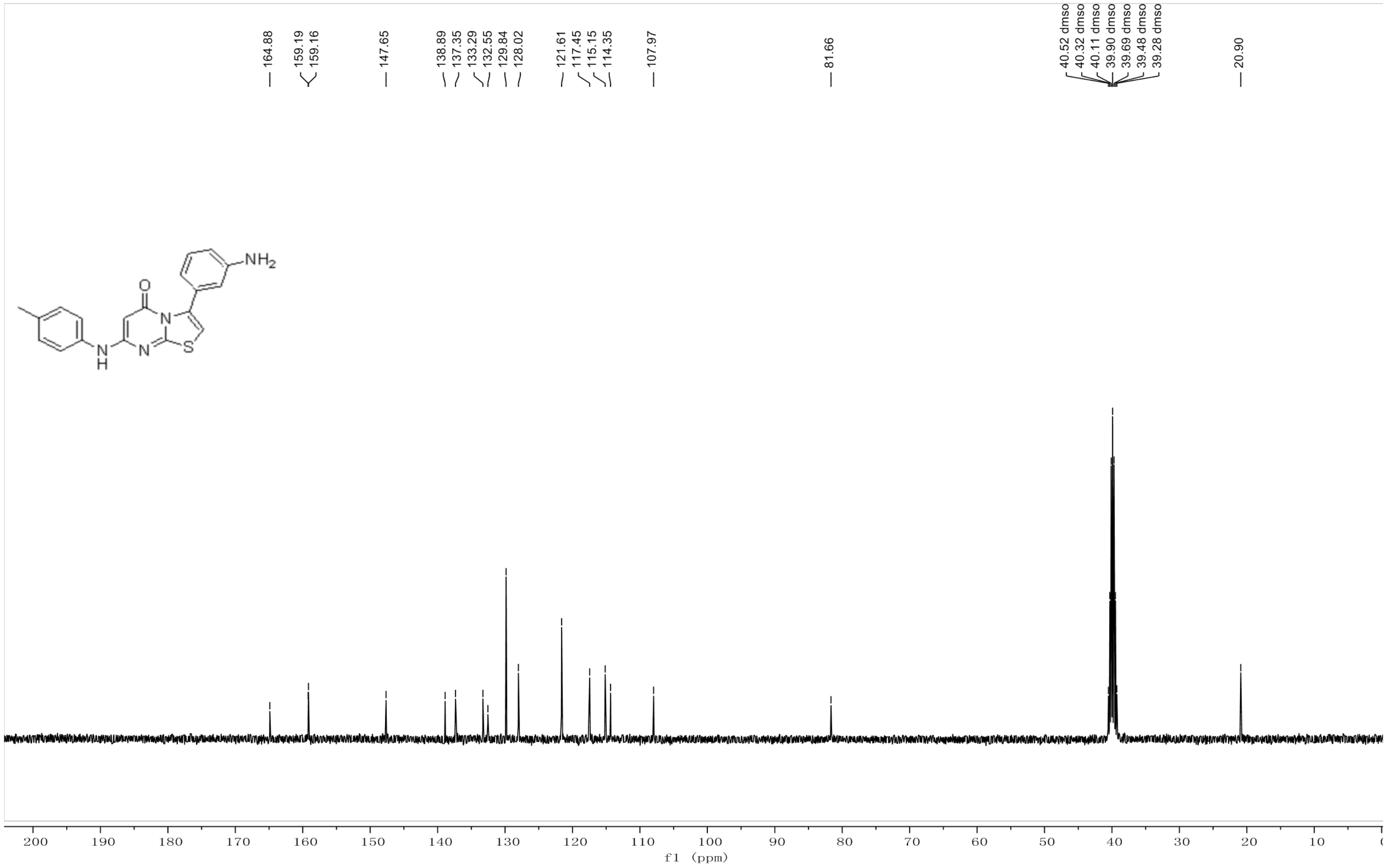

Figure S32. The ^{13}C-NMR spectra of **6a**: 3-(3-Aminophenyl)-7-(*p*-tolylamino)-5*H*-thiazolo[3,2-*a*]pyrimidin-5-one.

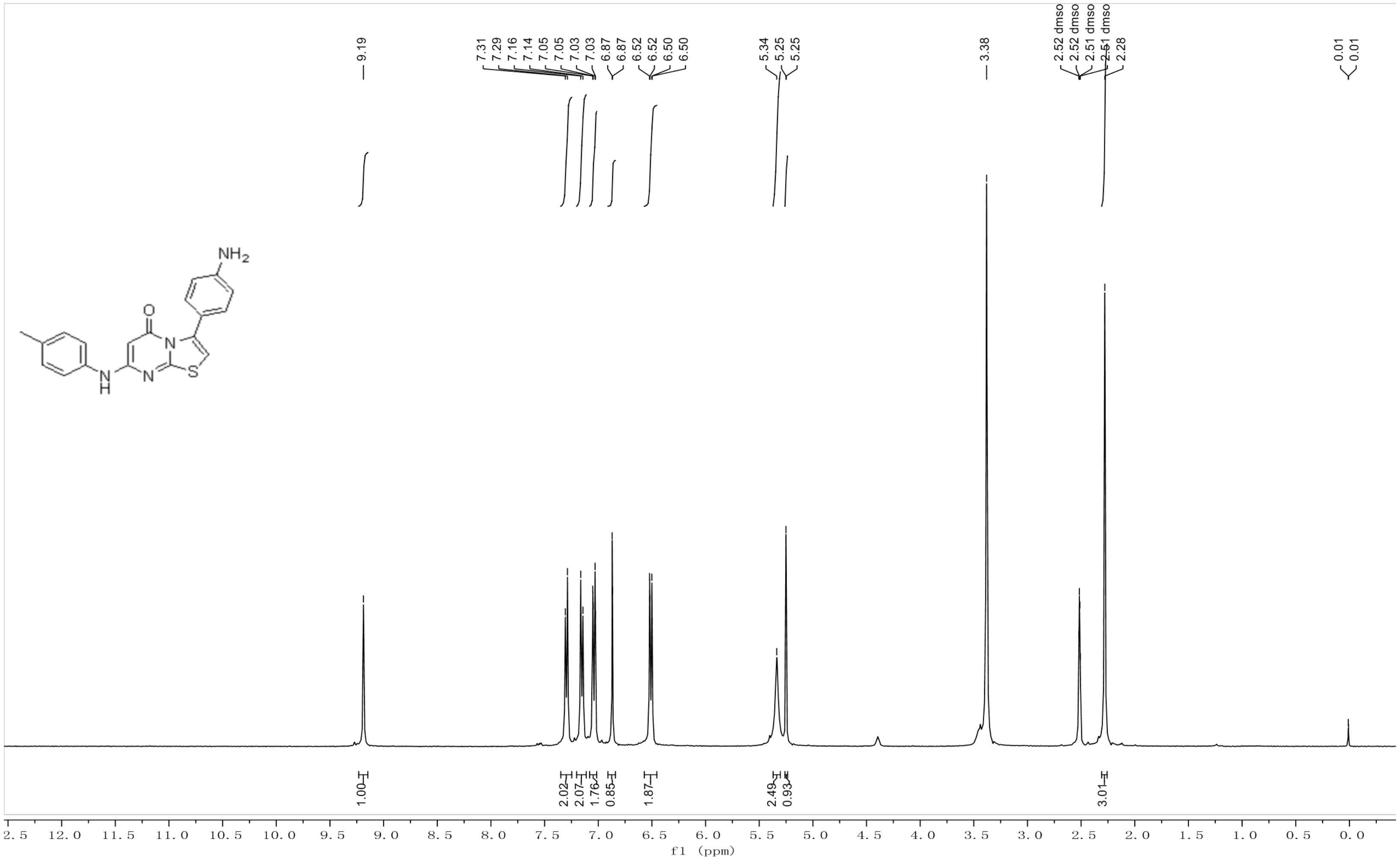

Figure S33. The ^{1}H-NMR spectra of **6b**: 3-(4-Aminophenyl)-7-(*p*-tolylamino)-5*H*-thiazolo[3,2-*a*]pyrimidin-5-one.

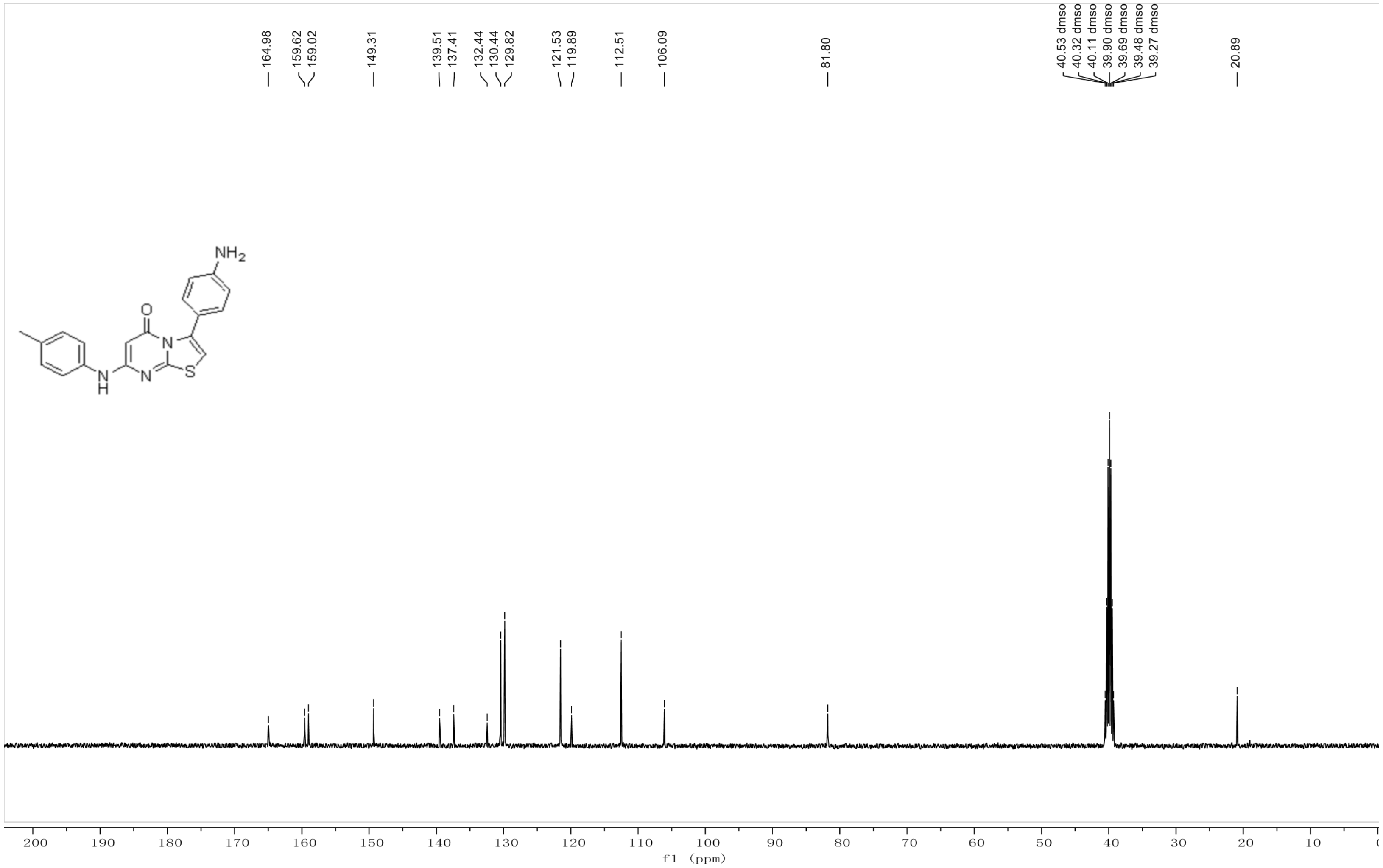

Figure S34. The ^{13}C-NMR spectra of **6b**: 3-(4-Aminophenyl)-7-(*p*-tolylamino)-5*H*-thiazolo[3,2-*a*]pyrimidin-5-one.

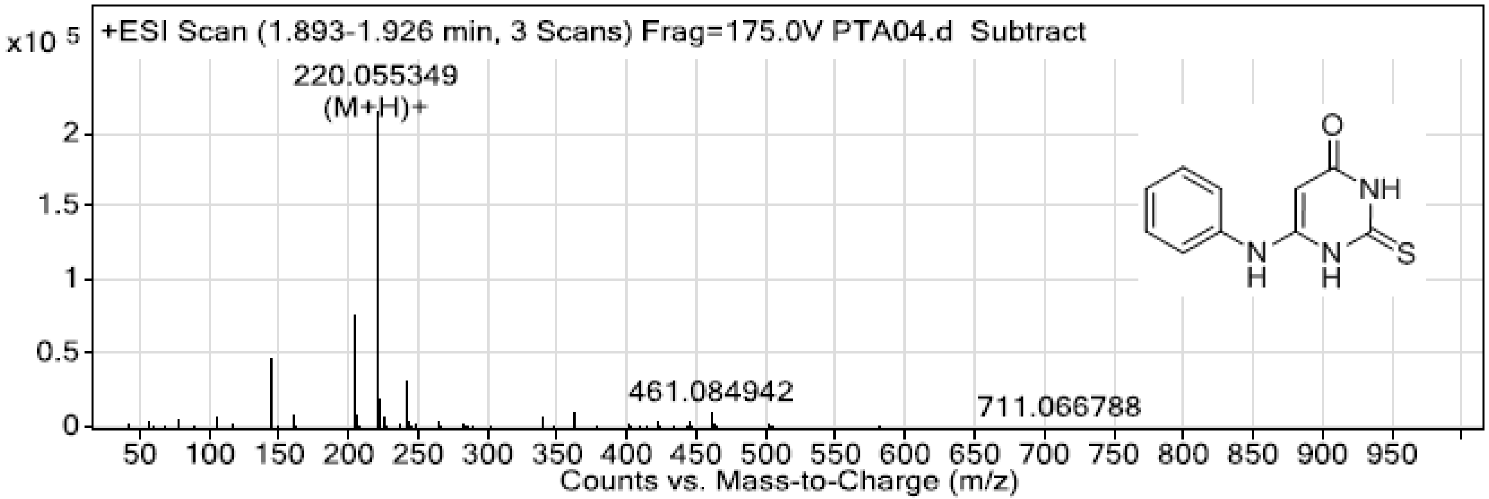

Figure S35. The HRMS spectra of **1a**: 6-(Phenylamino)-2-thioxo-2,3-dihydropyrimidin-4(1*H*)-one.

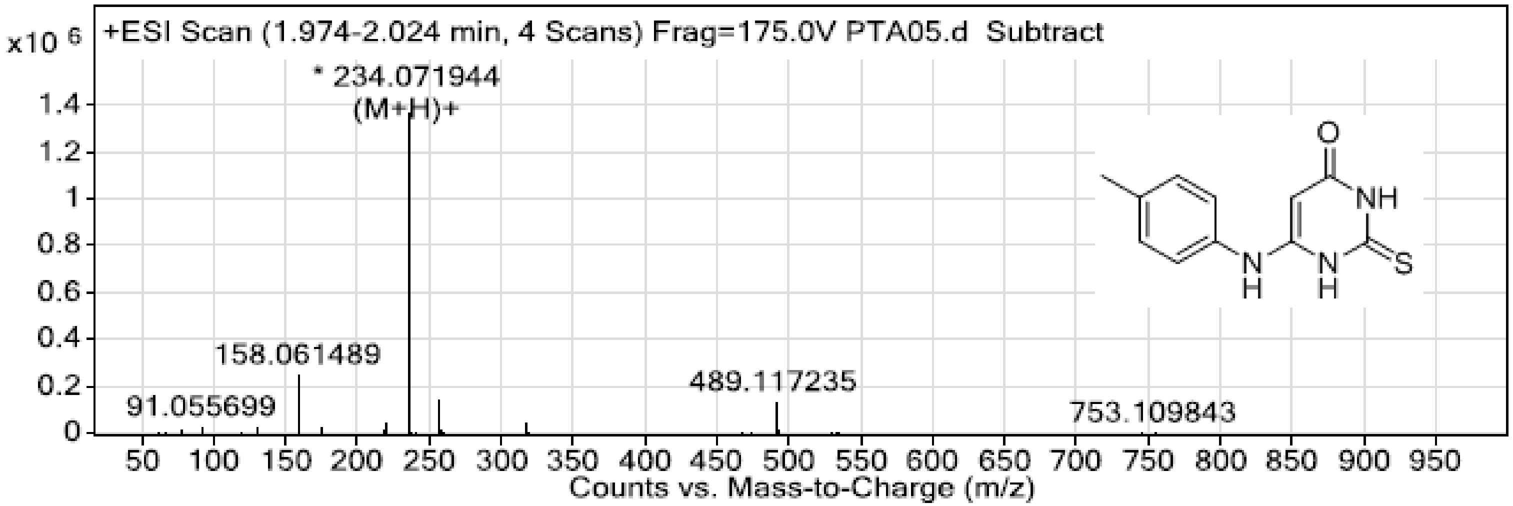

Figure S36. The HRMS spectra of **1b**: 2-Thioxo-6-(*p*-tolylamino)-2,3-dihydropyrimidin-4(1*H*)-one.

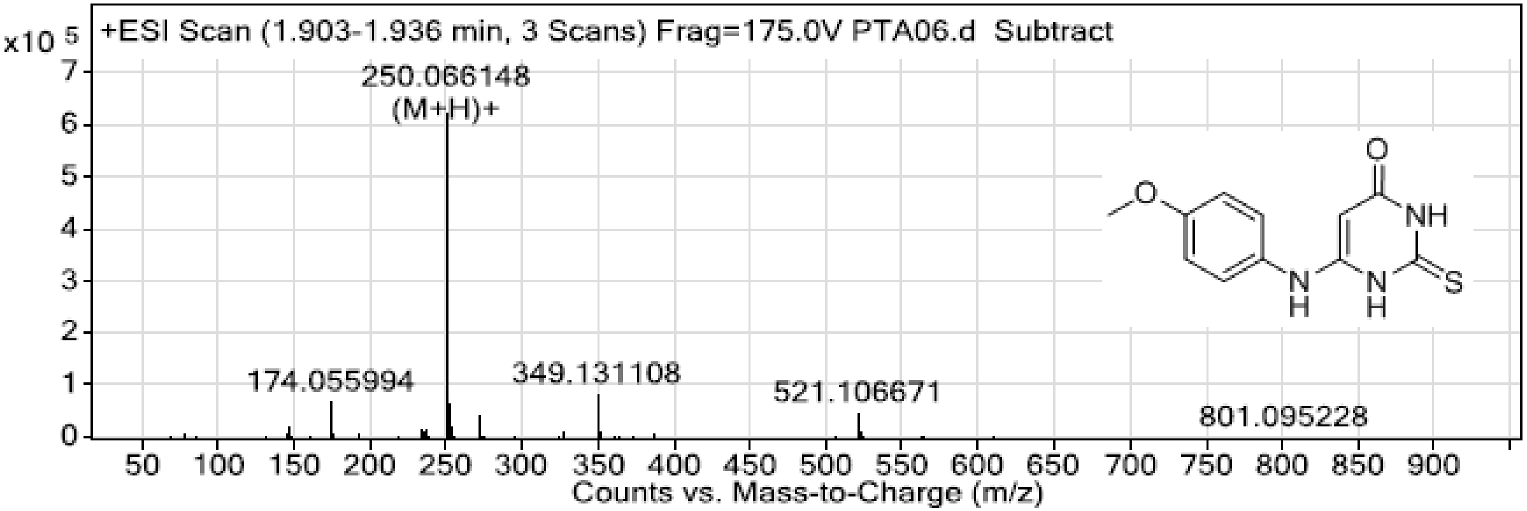

Figure S37. The HRMS spectra of **1c**: 6-((4-Methoxyphenyl)amino)-2-thioxo-2,3-dihydropyrimidin-4(1*H*)-one.

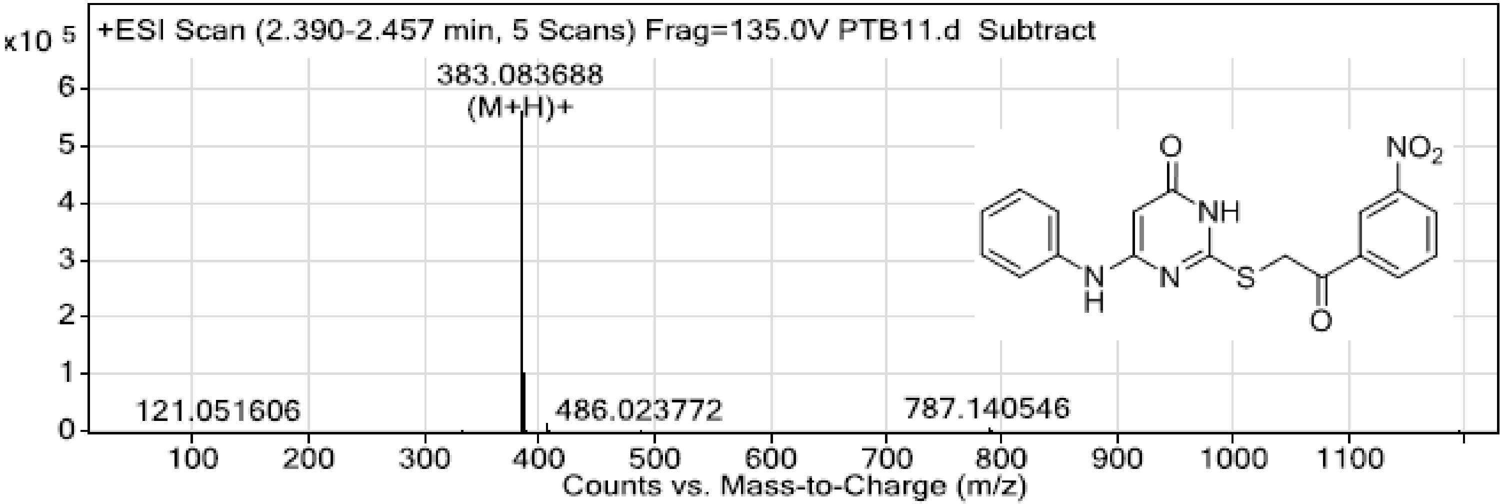

Figure S38. The HRMS spectra of **2a**: 2-((2-(3-Nitrophenyl)-2-oxoethyl)thio)-6-(phenylamino)pyrimidin-4(3*H*)-one.

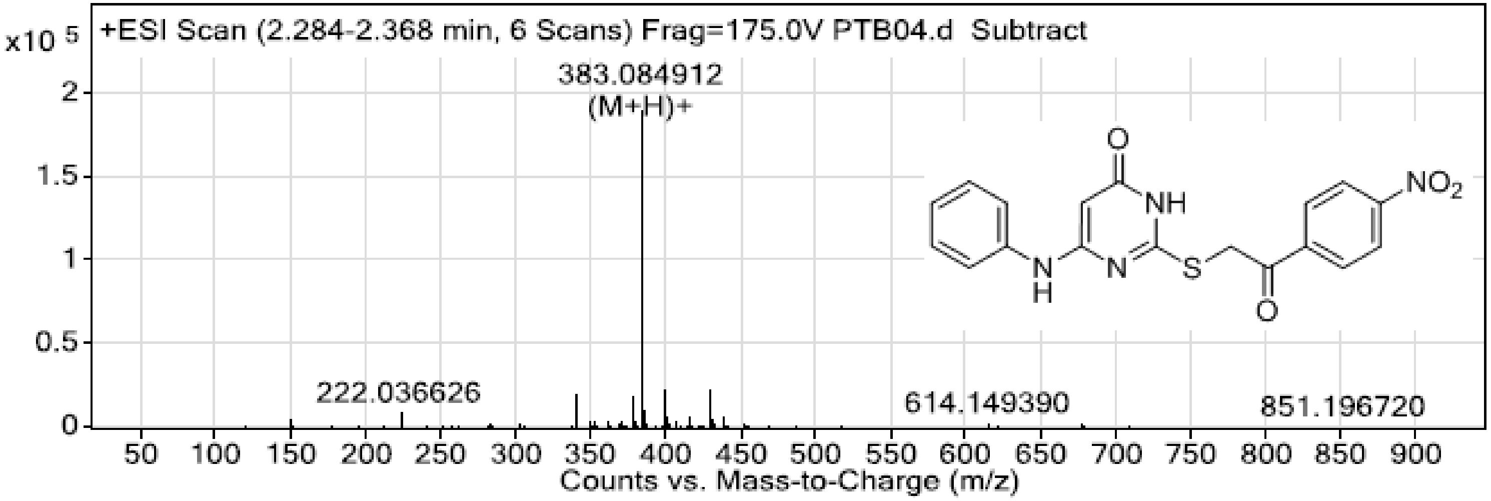

Figure S39. The HRMS spectra of **2b**: 2-((2-(4-Nitrophenyl)-2-oxoethyl)thio)-6-(phenylamino)pyrimidin-4(3*H*)-one.

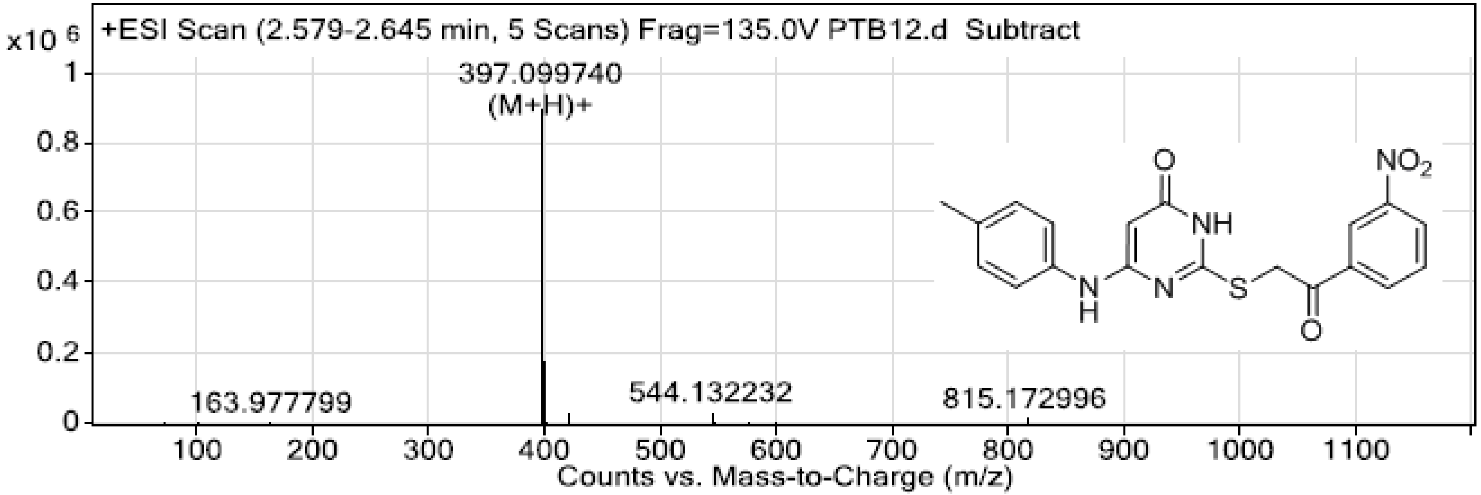

Figure S40. The HRMS spectra of **2c**: 2-((2-(3-Nitrophenyl)-2-oxoethyl)thio)-6-(*p*-tolylamino)pyrimidin-4(3*H*)-one.

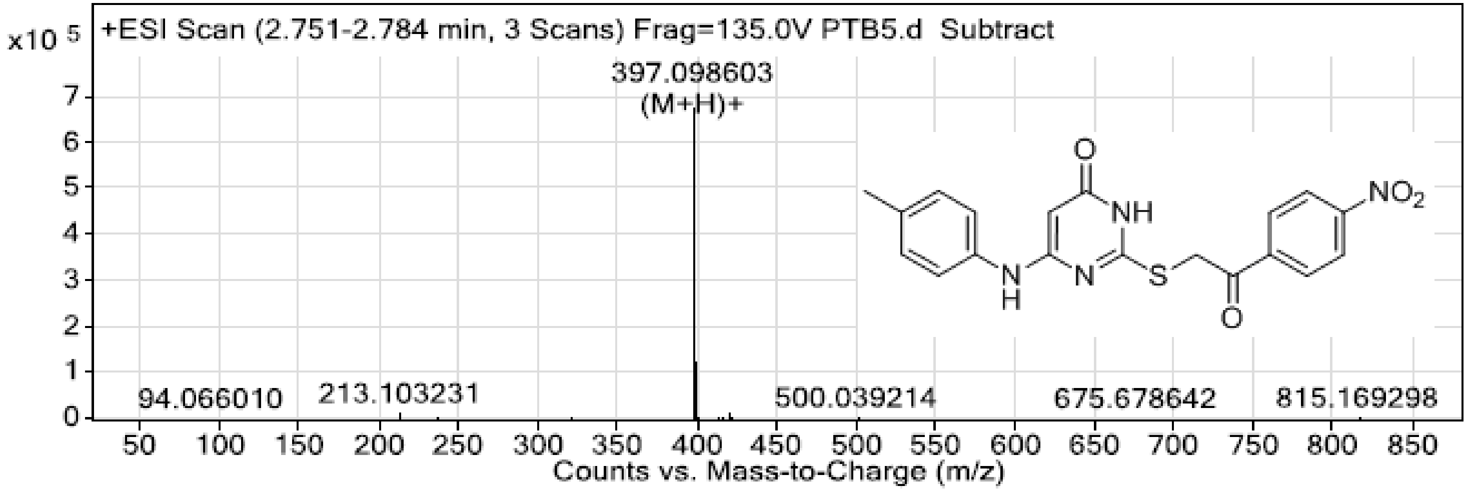

Figure 41S. The HRMS spectra of **2d**: 2-((2-(4-Nitrophenyl)-2-oxoethyl)thio)-6-(*p*-tolylamino)pyrimidin-4(3*H*)-one.

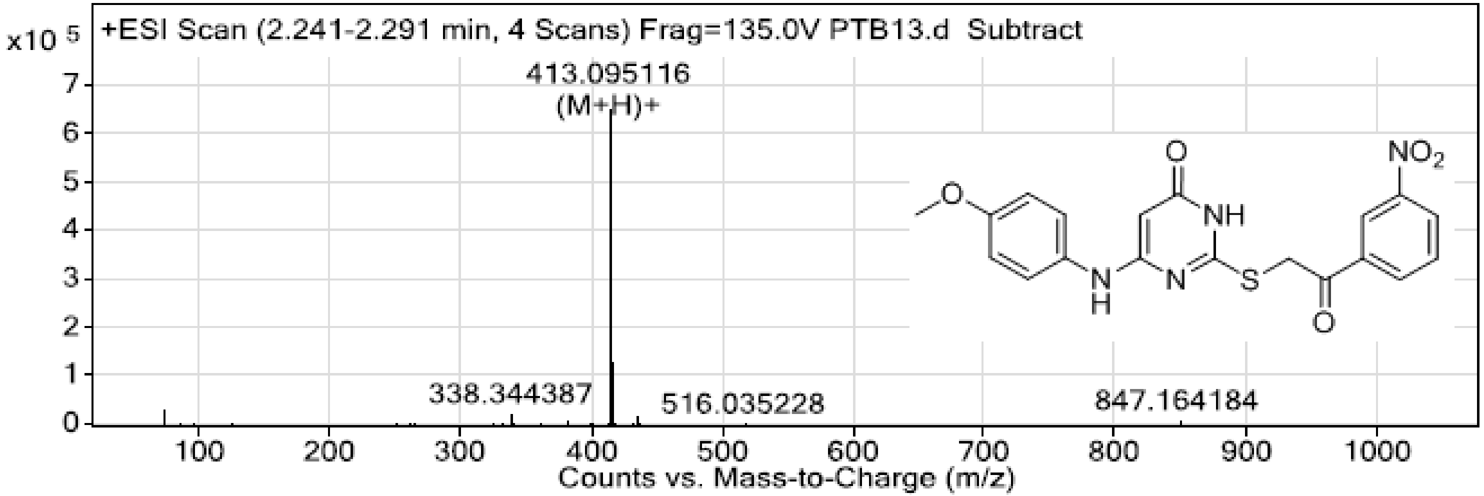

Figure S42. The HRMS spectra of **2e**: 6-((4-Methoxyphenyl)amino)-2-((2-(3-nitrophenyl)-2-oxoethyl)thio)pyrimidin-4(3*H*)-one.

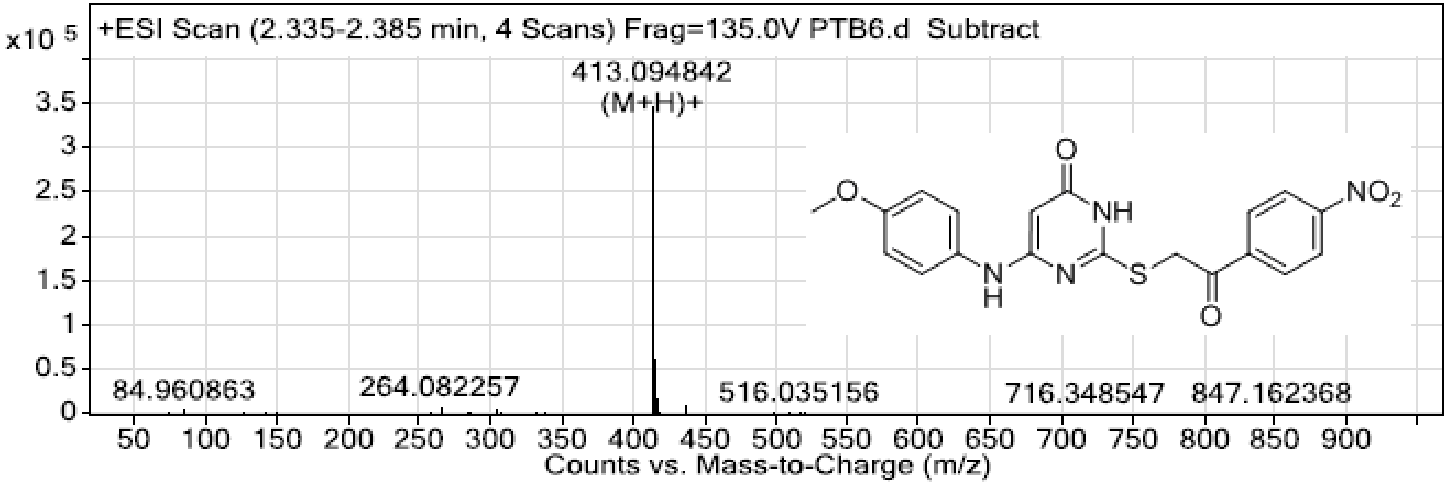

Figure S43. The HRMS spectra of **2f**: 6-((4-Methoxyphenyl)amino)-2-((2-(4-nitrophenyl)-2-oxoethyl)thio)pyrimidin-4(3*H*)-one.

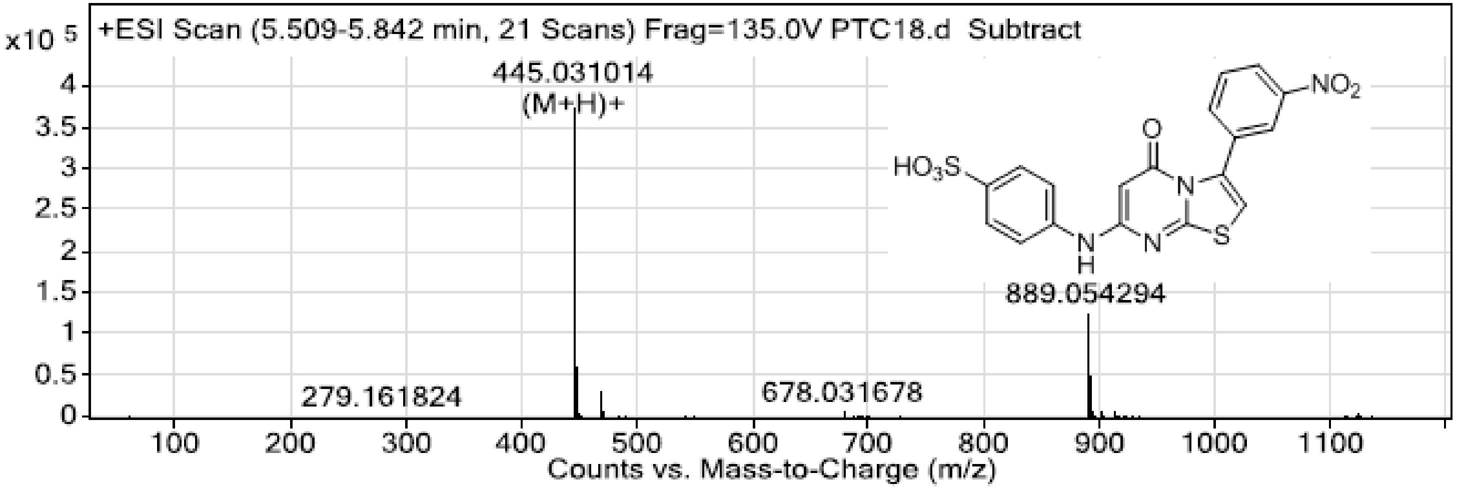

Figure S44. The HRMS spectra of **3a**: 4-((3-(3-Nitrophenyl)-5-oxo-5*H*-thiazolo[3,2-*a*]pyrimidin-7-yl)amino)benzenesulfonic acid.

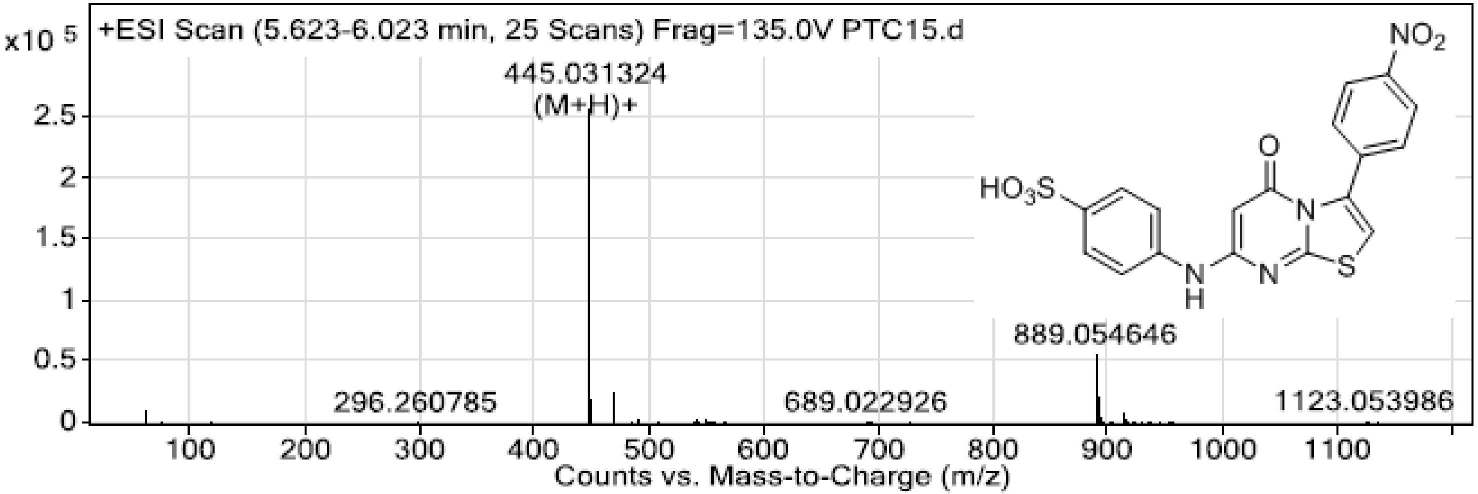

Figure S45. The HRMS spectra of **3b**: 4-((3-(4-Nitrophenyl)-5-oxo-5*H*-thiazolo[3,2-*a*]pyrimidin-7-yl)amino)benzenesulfonic acid.

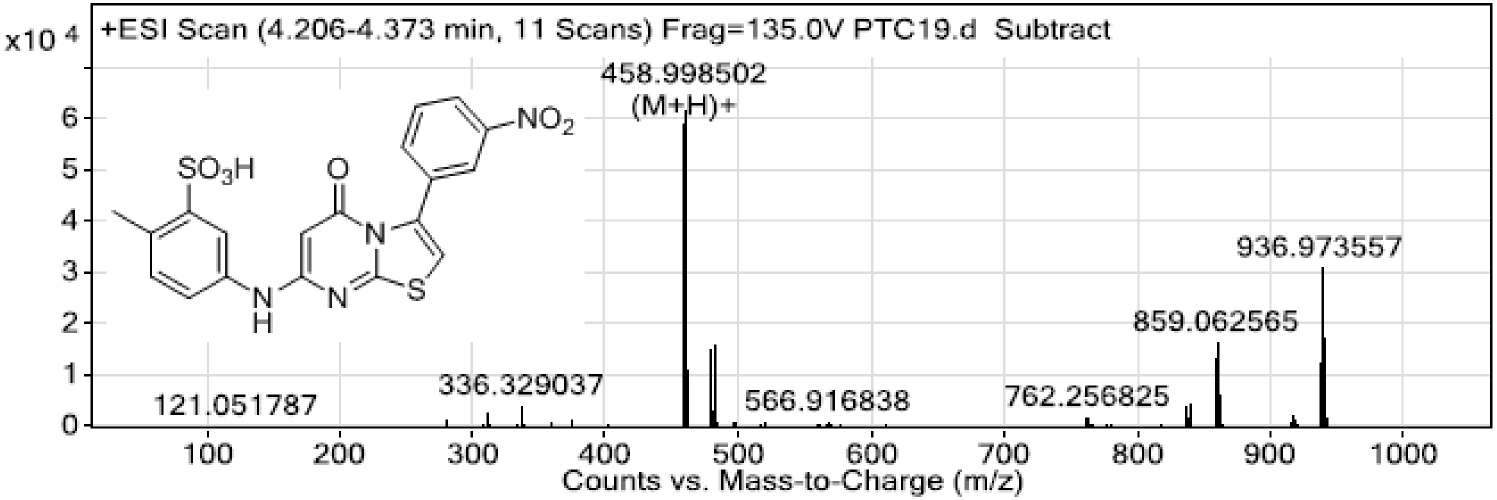

Figure S46. The HRMS spectra of **3c**: 2-Methyl-5-((3-(3-nitrophenyl)-5-oxo-5*H*-thiazolo[3,2-*a*]pyrimidin-7-yl)amino)benzenesulfonic acid.

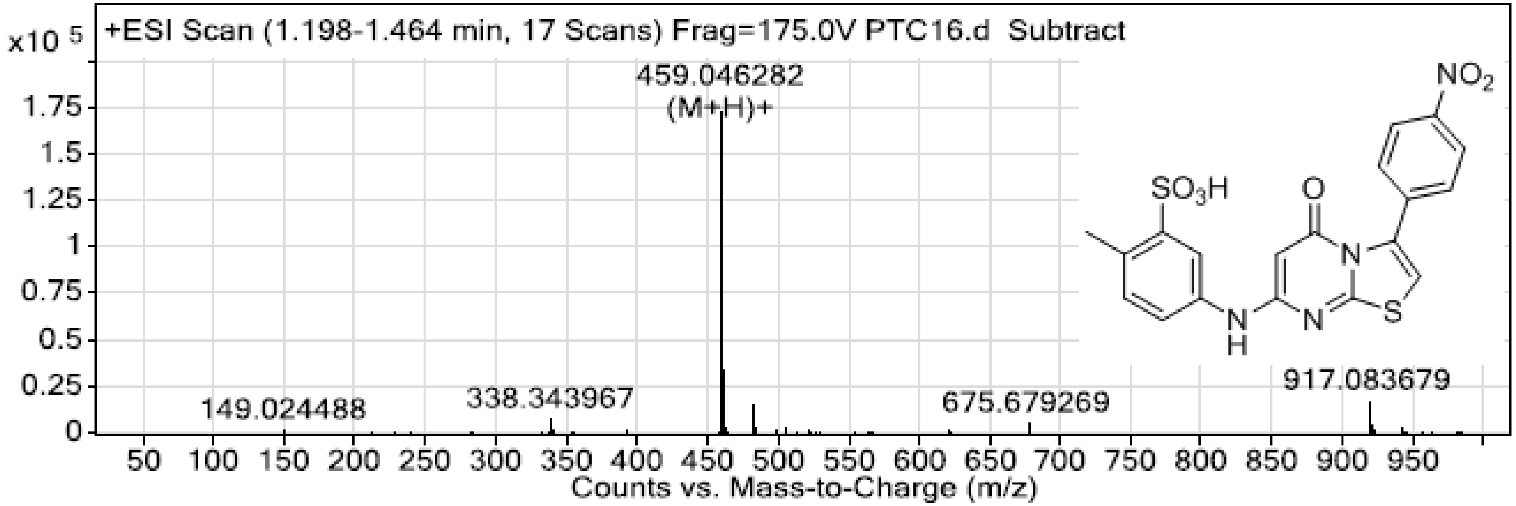

Figure S46. The HRMS spectra of **3d**: 2-Methyl-5-((3-(4-nitrophenyl)-5-oxo-5*H*-thiazolo[3,2-*a*]pyrimidin-7-yl)amino)benzenesulfonic acid.

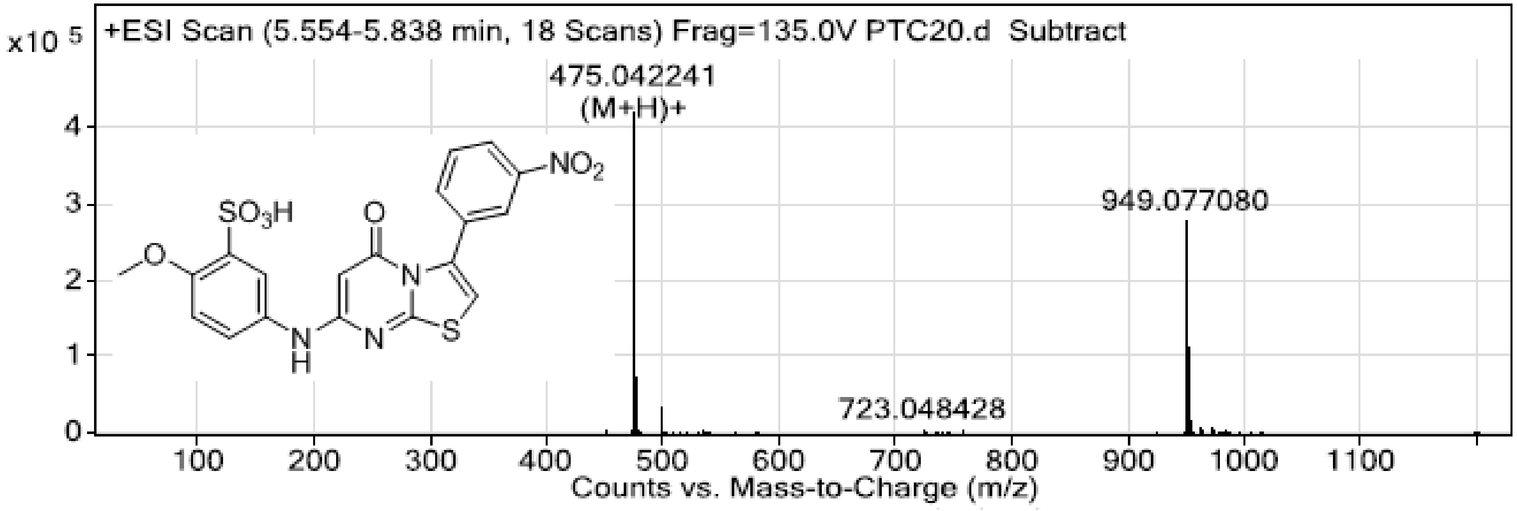

Figure S47. The HRMS spectra of **3e**: 2-Methoxy-5-((3-(3-nitrophenyl)-5-oxo-5*H*-thiazolo[3,2-*a*]pyrimidin-7-yl)amino)benzenesulfonic acid.

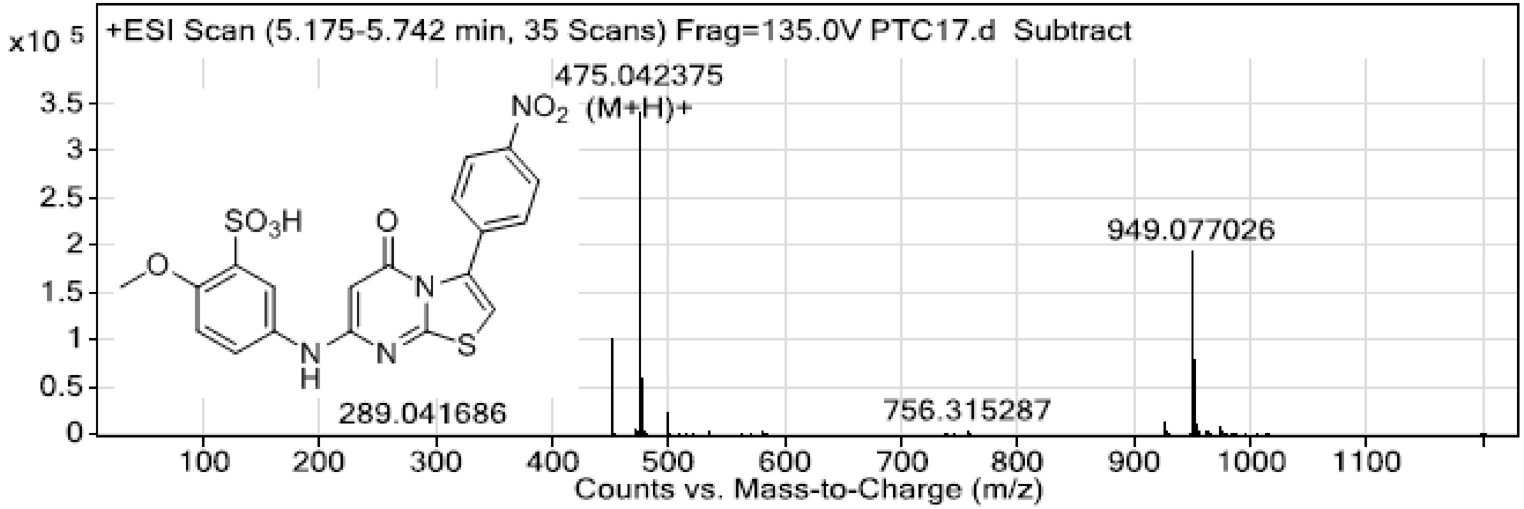

Figure S48. The HRMS spectra of **3f**: 2-Methoxy-5-((3-(4-nitrophenyl)-5-oxo-5*H*-thiazolo[3,2-*a*]pyrimidin-7-yl)amino)benzenesulfonic acid.

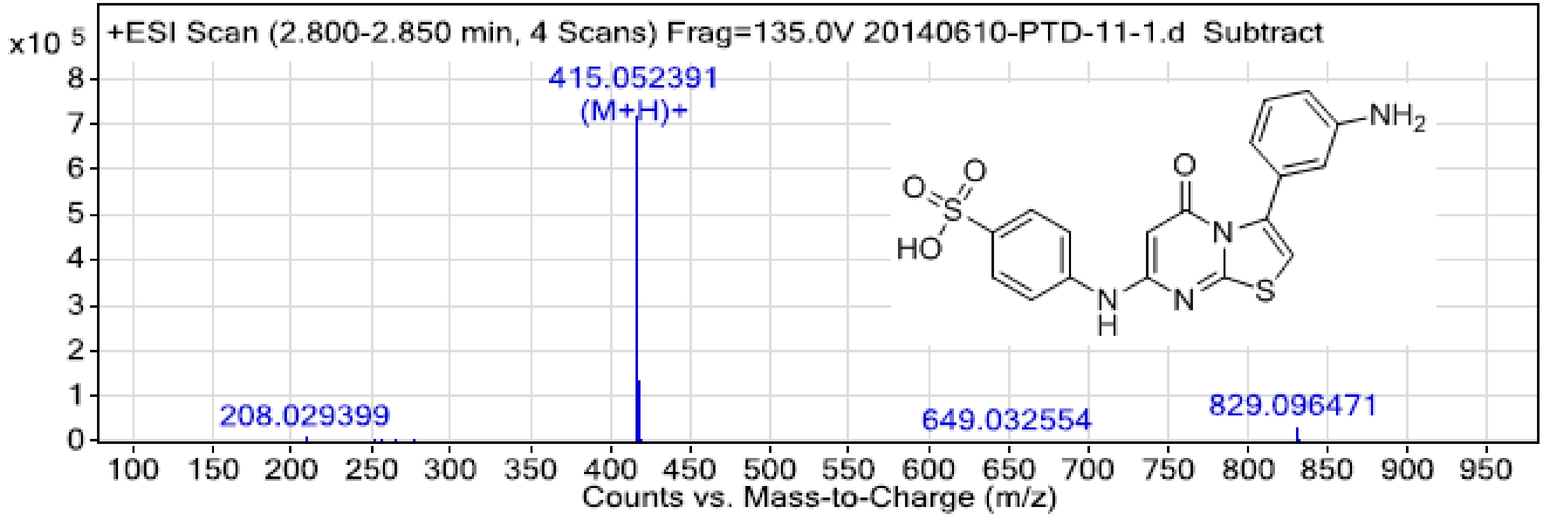

Figure S49. The HRMS spectra of **4a**: 4-((3-(3-Aminophenyl)-5-oxo-5*H*-thiazolo[3,2-*a*]pyrimidin-7-yl)amino)benzenesulfonic acid.

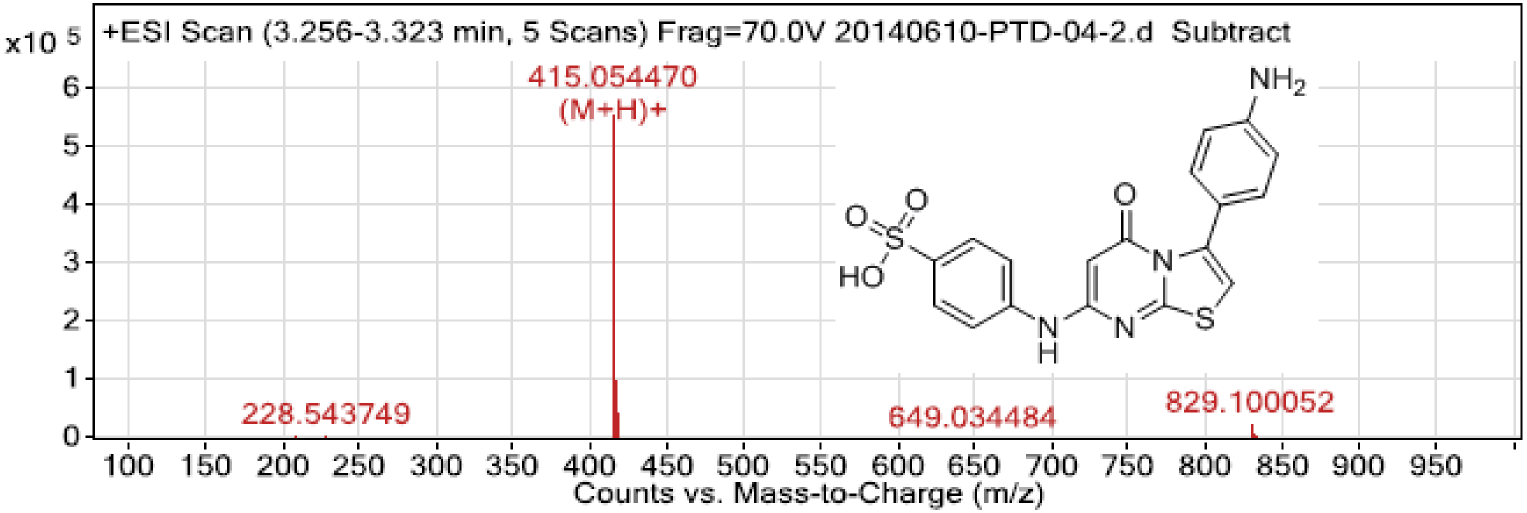

Figure S50. The HRMS spectra of **4b**: 4-((3-(4-Aminophenyl)-5-oxo-5*H*-thiazolo[3,2-*a*]pyrimidin-7-yl)amino)benzenesulfonic acid.

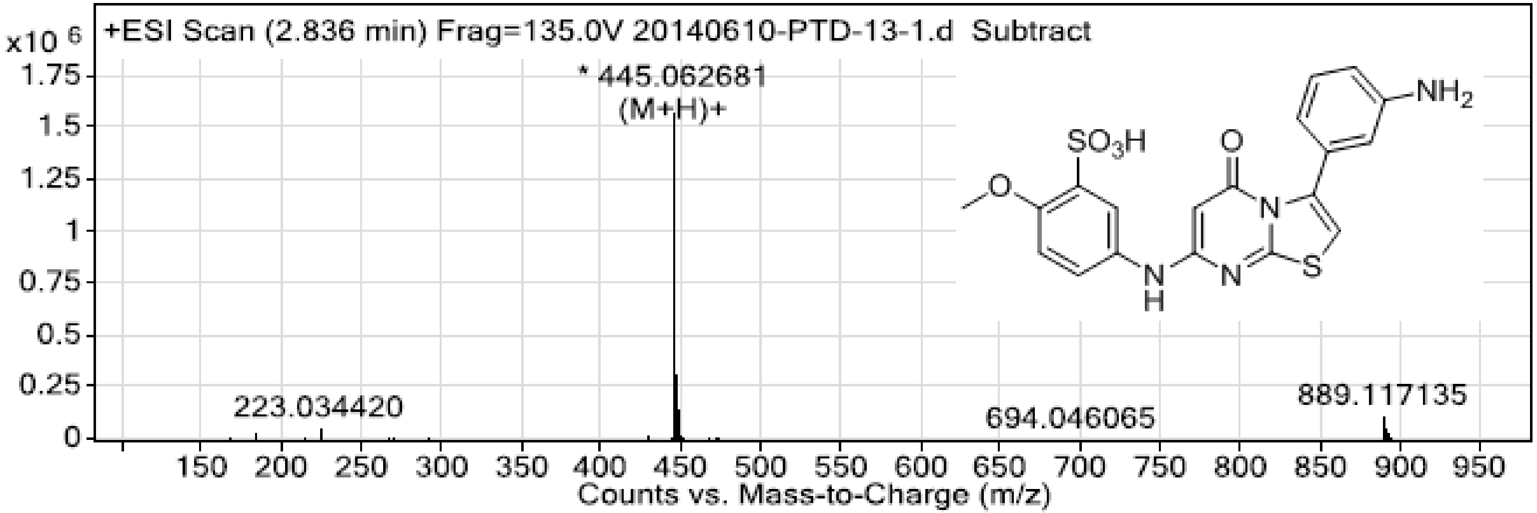

Figure S51. The HRMS spectra of **4c**: 5-((3-(3-Aminophenyl)-5-oxo-5*H*-thiazolo[3,2-*a*]pyrimidin-7-yl)amino)-2-methoxybenzenesulfonic acid.

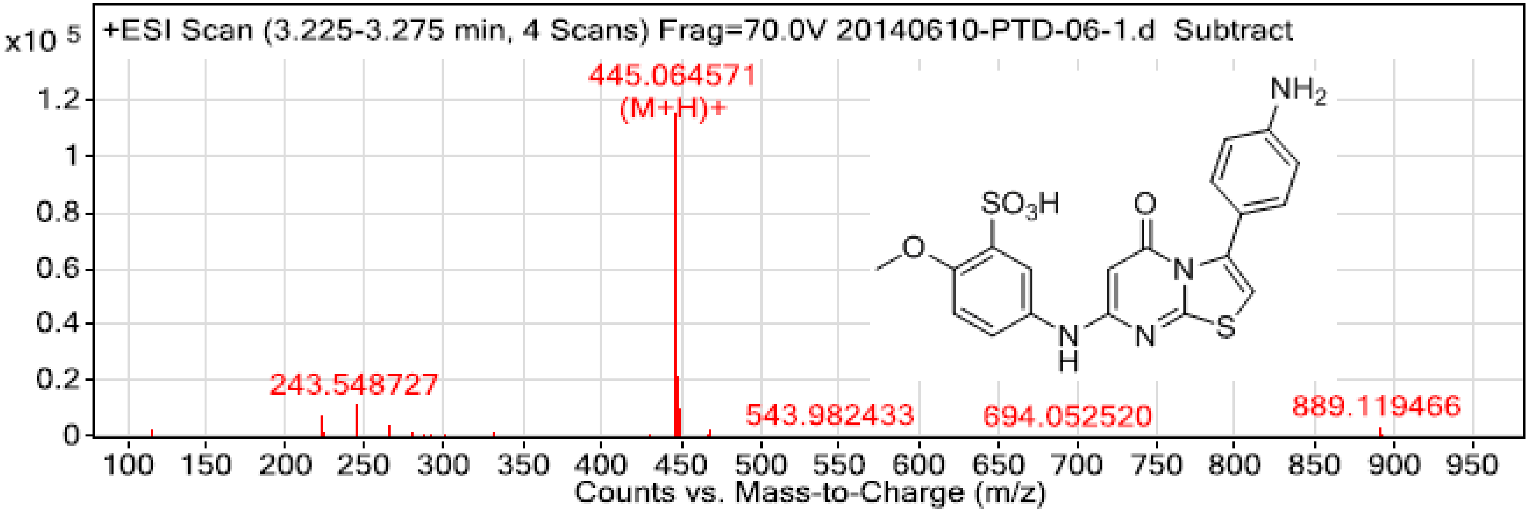

Figure S52. The HRMS spectra of **4d**: 5-((3-(4-Aminophenyl)-5-oxo-5*H*-thiazolo[3,2-*a*]pyrimidin-7-yl)amino)-2-methoxybenzenesulfonic acid.

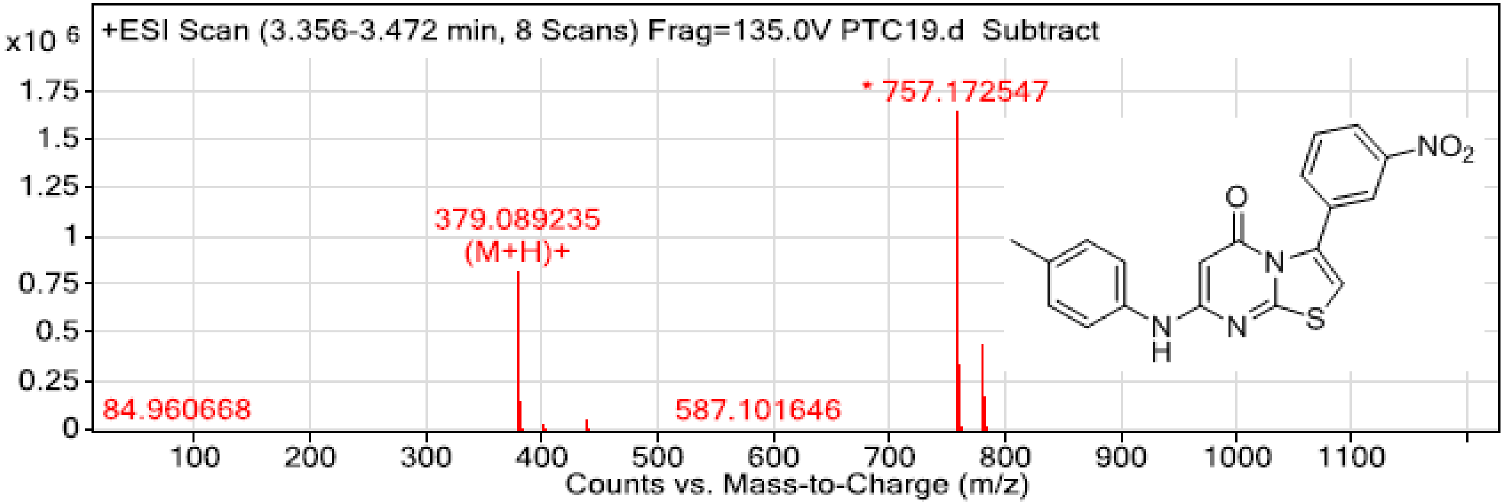

Figure S53. The HRMS spectra of **5a**: 3-(4-Nitrophenyl)-7-(*p*-tolylamino)-5H-thiazolo[3,2-*a*]pyrimidin-5-one.

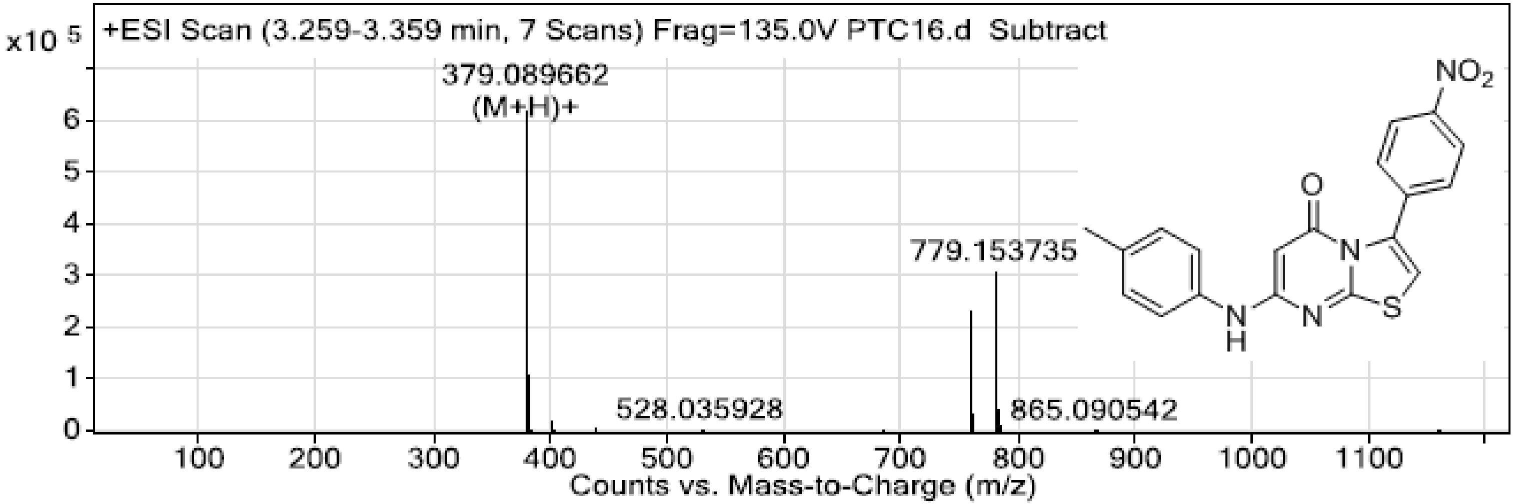

Figure S54. The HRMS spectra of **5b**: 3-(4-Nitrophenyl)-7-(*p*-tolylamino)-5*H*-thiazolo[3,2-*a*]pyrimidin-5-one.

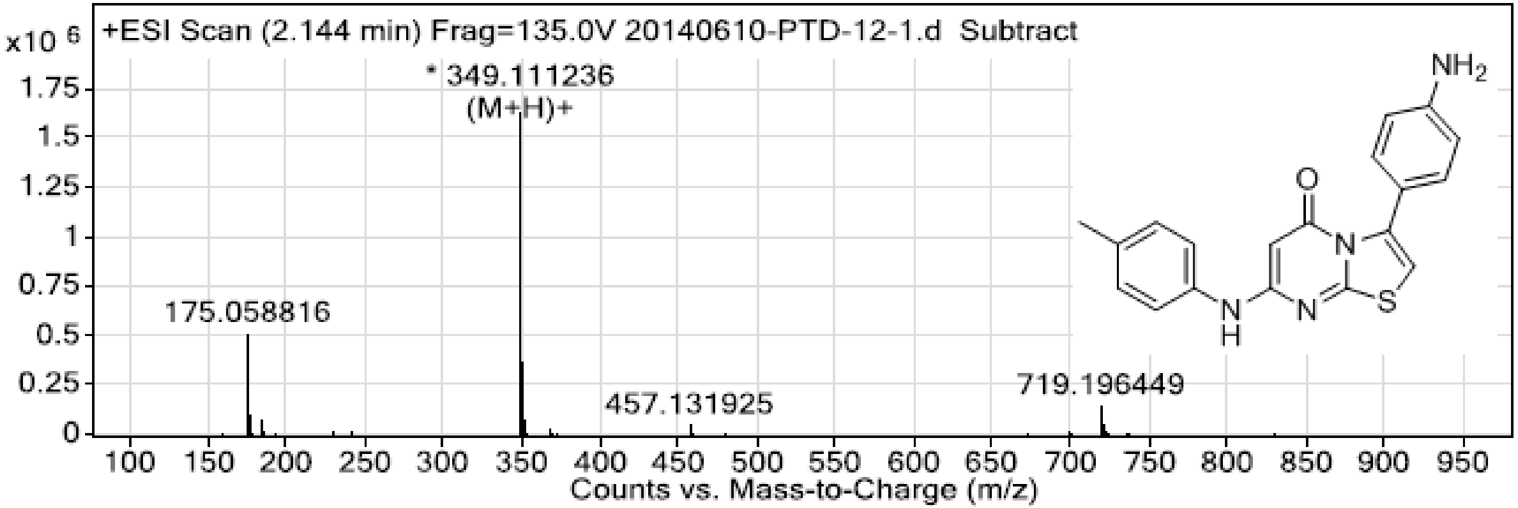

Figure S55. The HRMS spectra of **6a**: 3-(3-Aminophenyl)-7-(*p*-tolylamino)-5*H*-thiazolo[3,2-*a*]pyrimidin-5-one.

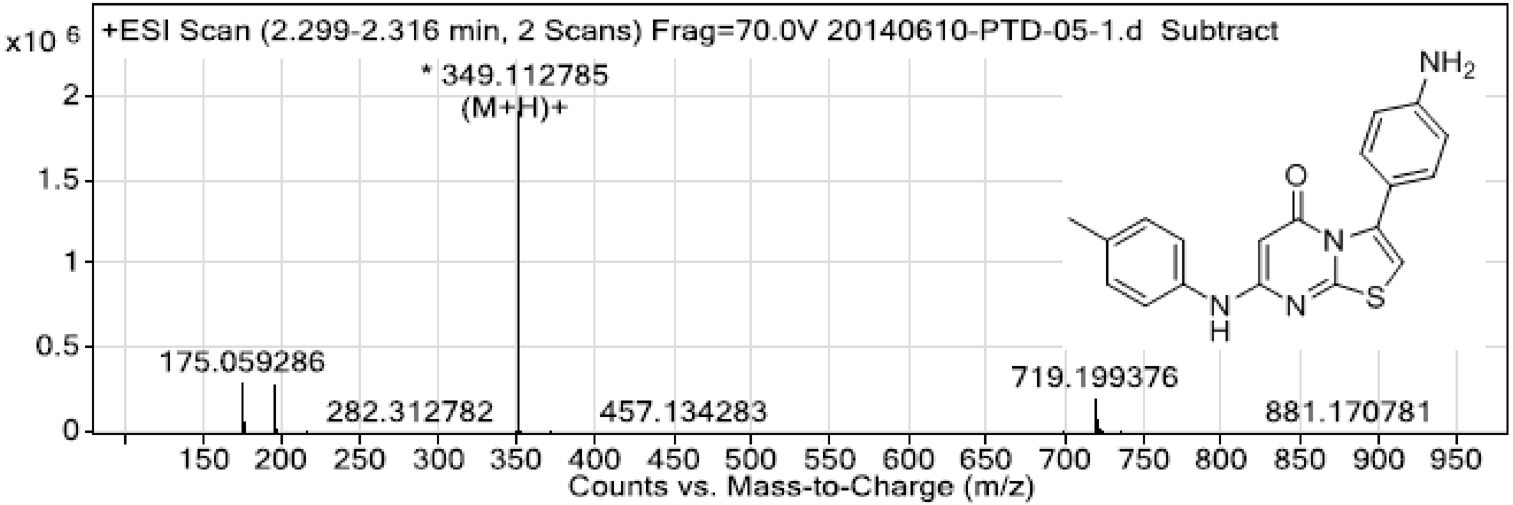

Figure S56. The HRMS spectra of **6b**: 3-(4-Aminophenyl)-7-(*p*-tolylamino)-5*H*-thiazolo[3,2-*a*]pyrimidin-5-one.
